# Mitochondrial DNA release via the mitochondrial permeability transition pore activates the cGAS-STING pathway, exacerbating inflammation in acute Kawasaki disease

**DOI:** 10.1186/s12964-024-01677-9

**Published:** 2024-06-13

**Authors:** Ke Wei, Tao Chen, Hao Fang, Xianjuan Shen, Zhiyuan Tang, Jianmei Zhao

**Affiliations:** 1 Department of Pediatrics, Affiliated Hospital of Nantong University, Nantong, 226001 Jiangsu Province China; 2 Research Institute of Comparative Medicine, Nantong University, Nantong, 226001 Jiangsu Province China; 3 Department of Clinical Laboratory, Affiliated Hospital of Nantong University, Nantong, 226001 Jiangsu Province China; 4 Department of Pharmacy, Affiliated Hospital of Nantong University, Nantong, 226001 Jiangsu Province China

**Keywords:** Kawasaki disease, mtDNA, cGAS-STING pathway, mPTP

## Abstract

**Background:**

Kawasaki disease (KD) is an immune vasculitis of unknown origin, characterized by transient inflammation. The activation of the cGAS-STING pathway, triggered by mitochondrial DNA (mtDNA) release, has been implicated in the onset of KD. However, its specific role in the progression of inflammation during KD's acute phase remains unclear.

**Methods:**

We measured mtDNA and 2’3’-cGAMP expression in KD patient serum using RT-qPCR and ELISA. A murine model of KD was induced by injecting Lactobacillus casei cell wall extract (LCWE), after which cGAS-STING pathway activation and inflammatory markers were assessed via immunohistochemistry, western blot, and RT-qPCR. Human umbilical vein endothelial cells (HUVECs) were treated with KD serum and modulators of the cGAS-STING pathway for comparative analysis. Mitochondrial function was evaluated using Mitosox staining, mPTP opening was quantified by fluorescence microscopy, and mitochondrial membrane potential (MMP) was determined with JC-1 staining.

**Results:**

KD patient serum exhibited increased mtDNA and 2’3’-cGAMP expression, with elevated levels of pathway-related proteins and inflammatory markers observed in both in vivo and in vitro models. TEM confirmed mitochondrial damage, and further studies demonstrated that inhibition of mPTP opening reduced mtDNA release, abrogated cGAS-STING pathway activation, and mitigated inflammation.

**Conclusion:**

These findings indicate that mtDNA released through the mPTP is a critical activator of the cGAS-STING pathway, contributing significantly to KD-associated inflammation. Targeting mtDNA release or the cGAS-STING pathway may offer novel therapeutic approaches for KD management.

**Supplementary Information:**

The online version contains supplementary material available at 10.1186/s12964-024-01677-9.

## Background

Kawasaki Disease (KD), a systemic vasculitis that predominantly impacts children between six months and five years of age, is characterized by systemic inflammation with the potential to cause coronary artery damage if left untreated. The disease's etiology remains enigmatic, yet its impact on long-term cardiovascular health is significant, emphasizing the necessity for a comprehensive understanding of KD pathogenesis to inform therapeutic strategies and prevent adverse outcomes [1]. Early and accurate diagnosis, coupled with prompt treatment, are paramount for optimizing clinical outcomes and exploring innovative treatment modalities.

Mitochondria, as essential cellular organelles, play a critical role in life-sustaining processes such as energy production and metabolism [2]. They harbor mitochondrial DNA (mtDNA), an innate immune agonist that, upon release, functions as a damage-associated molecular pattern (DAMP) [3]. The liberation of mtDNA can initiate a cascade of innate immune responses, particularly through the cGAS-STING signaling pathway, which is increasingly recognized for its role in sensing cytoplasmic dsDNA [4, 5].

Under conditions of cellular stress, such as those encountered in KD, mitochondria may undergo electron transport chain (ETC) dysfunction, leading to heightened reactive oxygen species (ROS) production, ATP depletion, and cellular damage [6]. This pathophysiological state can precipitate the release of mtDNA into the cytoplasm, where it is subject to oxidative modification [7]. Prior research has documented elevated levels of circulating free mtDNA in KD patients, suggesting a potential link between mitochondrial dysfunction and KD pathogenesis [8]. The mitochondrial permeability transition pore (mPTP) is a critical regulator of mtDNA release, and its modulation presents a promising therapeutic target for KD management [9].

The cGAS-STING pathway, integral to systemic inflammatory responses and autoimmune activation, is activated upon cGAS recognition of dsDNA, culminating in the production of the secondary messenger cGAMP and subsequent STING activation. This interaction leads to the nuclear translocation of P65 and IRF3, which orchestrate the expression of inflammatory mediators and type I interferons [10]. The significance of the cGAS-STING pathway in autoimmune diseases is increasingly acknowledged, with recent studies underscoring its role in diseases such as systemic lupus erythematosus and rheumatoid arthritis [11–13].

This study aims to delineate the role of mtDNA and the cGAS-STING axis in KD pathogenesis, with a focus on the potential of mPTP modulation to attenuate inflammation and disease progression. By examining the interplay between mtDNA release and the cGAS-STING pathway, we aim to contribute to the identification of novel therapeutic targets for KD.

## Materials and methods

### Patient blood samples and ethical considerations

We collected blood samples and clinical data from patients diagnosed with Kawasaki disease (KD), febrile patients, and healthy pediatric controls at the Affiliated Hospital of Nantong University from 2022 to 2023. The age ranges of the selected samples did not show statistical significance (*p* < 0.05). Healthy children ranged from two months to 10 years and six months old, children with KD ranged from one month and two days to eight years and one month old, and febrile children ranged from three months to nine years and three months old. KD diagnosis adhered to the criteria set forth by the American Heart Association in 2017 [14]. KD patients in the acute phase, untreated with intravenous immunoglobulin (IVIG; 2 g/kg) or aspirin (30–50 mg/kg/day), were included. We also included age-matched febrile patients without KD, who had not received antibiotics and were free from other illnesses. All samples were promptly stored at -80 °C post-collection. Informed consent was obtained from participants or their guardians, and the study was conducted with the approval of the Nantong University Hospital Ethics Committee (Approval No. 2022-L072), in accordance with the Declaration of Helsinki.

### Cell culture

Human umbilical vein endothelial cells (HUVECs) were generously provided by the laboratory of the Department of Obstetrics and Gynaecology, Affiliated Hospital of Nantong University, and confirmed to be free of mycoplasma contamination. HUVECs cells were seeded in cell culture dishes and cultured routinely in DMEM-F12 (F-12; Gibco) medium supplemented with 10% fetal bovine serum (FBS; Gibco) and 1% penicillin–streptomycin. The cultures were maintained at 37 °C in a humidified atmosphere with 5% CO_2_. The serum we collected was inactivated by a 56 °C water bath for 30 min, followed by 30 min of UV irradiation after inactivation. Subsequently, the solution was filtered through a 0.22-micron filter membrane for use in cell experiments. Random selection of patient serum with no differences in baseline characteristics for cell experiments. For experimental groups: HUVECs in the KD group were treated with inactivated serum obtained from KD patients. HUVECs in the control group were cultured with inactivated serum from healthy children. HUVECs in theH-151/ CsA /G150 group were cultured with inactivated serum from KD patients who had been pre-treated with H-151 (H-151, MCE) / cyclosporine (CsA, MCE) /G150 (G150, MCE) for 1 h. All cells were maintained under standard culture conditions.

### Preparation of *lactobacillus* casei cell wall extract (LCWE)

LCWE was prepared as per established protocols [15, 16]. Lactobacillus casei, preserved at -80 °C, was cultured in broth medium at a constant temperature of 37 °C for 48 h with shaking. Post-incubation, the bacterial cells were treated with DNase, RNase, and trypsin for 4 h, followed by washing with phosphate-buffered saline (PBS). The cell wall components were then lysed using ultrasonication, and the supernatant obtained post-centrifugation was defined as LCWE. The concentration of LCWE was ascertained based on rhamnose content, determined using a colorimetric assay with phenol–sulfuric acid. The prepared LCWE was subsequently administered via intraperitoneal injection.

### KD animal model and experimental design

The animal model for KD was induced as described by Lehman et al. [15]. Male C57BL/6 mice, aged three to four weeks, were housed under specific pathogen-free conditions at the Nantong University Laboratory Animal Centre. Following a one-week acclimation period, mice were randomly allocated into three groups: the PBS Group (Control), the LCWE Group (KD Model), and the LCWE + H-151 Group (H-151 Treatment), with 20 mice per group. The KD and H-151 groups received a single intraperitoneal injection of 1 mL LCWE, while the control group was injected with an equivalent volume of PBS. The H-151 group was further administered H-151 (7 mg/kg) intraperitoneally on alternate days post-LCWE injection. Ethical approval for these procedures was granted by the Animal Ethics Committee (Approval No. S20231209-004).

### Enzyme-linked immunosorbent assay (ELISA)

Enzyme-linked immunosorbent assays were utilized to quantify serum 2'3’cGAMP levels, employing commercially available ELISA kits from Preferred Bioscience & Technology Co. (Shanghai). The assays were performed in strict accordance with the manufacturer's guidelines.

### Real-time quantitative polymerase chain reaction (RT-qPCR)

For RT-qPCR, cells were cultured in 6-well plates as per the experimental design, and tissues from animal groups were homogenized after weighing. Total RNA was extracted from cellular and tissue samples using TRIzol reagent. The RNA was then reverse transcribed to complementary DNA (cDNA), followed by amplification using ChamQ SYBR qPCR Master Mix (Vazyme) under the following conditions: 40 cycles of amplification with three replicates per sample. Plasma from blood samples was isolated by centrifugation at 3500 rpm for 5 min. The relative expression levels of mitochondrial genes ND1 and COX1, normalized to nuclear DNA (18S), were determined using SYBR Green-based qPCR. The comparative cycle threshold (2^-ΔΔCt) method was employed to calculate relative gene expression. Primer sequences for RT-qPCR are detailed in Tables 1 and 2 of the supplementary materials. The mRNA expression levels of ND1 and COX1 were normalized to 18S RNA, expressed as ND1/18S and COX1/18S ratios. Other target genes were normalized to GAPDH for relative expression analysis.

**Table 1:** Primer sequences of the study

**Genes **(**Human**)	**Forward primer 5' → 3'**	**Reverse primer 5' → 3'**
GAPDH	CACGGCAAATTCCACGGCACAGT	GGGGGCATCAGCAGAAGGAGCAG
18S	GTAACCCGTTGAACCCCATT	CCATCCAATCGGTAGTAGCG
ND1	CCCTAAAACCCGCCACATCT	GAGCGATGGTGAGAGCTAAGGT
COX1	TCATCTGTAGGCTCATTC	GGCATCCATATAGTCACT
IL-6	GTCAACTCCATCTGCCCTTCAG	GGTCTGTTGTGGGTGGTATCCT
IP10	AGAACTGTACGCTGTACCTG	GTAGCAATGATCTCAACACG
IFNα	GGAGGAGTTTGATGGCAACC	ATCCCAAGCAGCAGATGAAT
IFNβ	CTTGGATTCCTACAAAGAAGCAGC	TCCTCCTTCTGGAACTGCTGCA

**Table 2:** Primer sequences of the study

**Genes **(**Mouse**)	**Forward primer 5' → 3'**	**Reverse primer 5' → 3'**
GAPDH	GGTTGTCTCCTGCGACTTCA	TGGTCCAGGTTTCTTACTCC
18S	TCCCCATGAACGAGGAATTC	CCGAGGGCCTCACTAAACC
ND1	TTGCACCTACCCTATCACTCA	CGGCTCGTAAAGCTCCGAAT
COX1	GACCGCAACCTAAACACAAC	GGTGCCCAAAGAATCAGAA
IL-6	ACAGAAGGAGTGGCTAAGGACC	TAGGCATAACGCACTAGGTTT
IP10	GTCATTTTCTGCCTCATCCT	GCCCTTTTAGACCTTTTTTG
IFNα	GCTAGGCTCTGTGCTTTCCT	CCTGCGGGAATCCAAAGTC
IFNβ	AGCACTGGGTGGAATGAGAC	GAGTCCGCCTCTGATGCTTA

### Transmission *electron* microscopy (TEM)

Transmission electron microscopy (TEM) was performed to observe cellular and tissue ultrastructure after treatment. After treatment, cultured cells or tissues were fixed sequentially with the following solutions: 2.5% glutaraldehyde (Servicebio) in the dark for 0.5 h. 2% osmium tetroxide for 2 h. Following fixation, samples were washed with double-distilled water. For cell samples: They were stained with 0.5% uranyl acetate for 12 h. After staining, samples underwent the following steps: Dehydration, Polymerization, Cutting into ultrathin sections of 70–90 nm. The prepared samples were then observed using a Tecnai G2 TWIN TEM (FEI).

### Immunohistochemical analysis

Heart tissues were embedded in paraffin, sectioned at 5-µm thickness, and subjected to hematoxylin and eosin (H&E) staining for histological assessment. For immunohistochemical detection of cGAS and STING, endogenous peroxidase activity was quenched with H2O2, and nonspecific binding was blocked with bovine serum albumin (BSA). Primary antibodies against cGAS and STING were applied, followed by horseradish peroxidase (HRP)-conjugated secondary antibodies. The immune complexes were visualized using diaminobenzidine (DAB), and slides were counterstained with hematoxylin. Integrated optical density (IOD) was measured using ImageJ software to quantify positive staining.

### Immunofluorescence assay

HUVECs were seeded on glass slides and fixed with ice-cold methanol. After blocking with a rapid blocking solution, the cells were incubated overnight at 4 °C in a humid environment with primary antibodies against STING (1:100, protein) and the Golgi apparatus (1:50, Sanu). Following incubation, they were incubated with Alexa Fluor 488-conjugated (Beyotime) and Alexa Fluor 568-conjugated (Beyotime) secondary antibodies for 2 h in the dark to detect the signals. Then stained with Hoechst-containing anti-fluorescence quenching blocking solution (Beyotime). Finally, invert the stained cell slides onto glass slides for observation. Labeled HUVECs were visualized under a confocal microscope (ZEISS LSM 900). Performing immunofluorescence colocalization analysis using ImageJ software.

### CCK8 assay

HUVECs were seeded in 96-well plates and treated with various concentrations of H-151 and CsA. After 24 h, the Cell Counting Kit-8 (CCK8) reagent was added, and absorbance was measured at 450 nm using a microplate reader to assess cell viability.

### Wound healing assay

HUVECs were cultured to confluence in 6-well plates and subjected to a wound by scratching with a pipette tip. Cells were then incubated in serum-free medium, and wound closure was monitored over 24 h using an inverted microscope. The healing rate was calculated as the percentage reduction in wound width. The healing rate of the scratches was calculated using the formula: Healing rate of the scratches = [(0 h scratch width—24 h scratch width) / 0 h scratch width] × 100%.

### Protein extraction and western blot analysis

Proteins were extracted from HUVECs using RIPA buffer containing protease and phosphatase inhibitors. For animal tissues, 100 mg of mouse heart tissue was taken and homogenized with RIPA lysis buffer containing protease inhibitors, phosphatase inhibitors, and PMSF, along with magnetic beads, for protein extraction. The homogenate was then centrifuged at 4000 rpm for 10 min, and the supernatant was collected as total protein. Protein concentrations were determined using a BCA Protein Assay Kit. Equal amounts of protein were separated by SDS-PAGE and transferred onto PVDF membranes. Membranes were probed with primary antibodies against cGAS, STING, P-TBK1, TBK1, P-P65, P65, P-IRF3, IRF3, TFAM, VDAC, TOM20, and β-actin, followed by HRP-conjugated secondary antibodies. Bands were visualized using a chemiluminescent substrate, and band intensities were quantified with ImageJ software.

### Measurement of mitochondrial reactive oxygen species (ROS)

To measure mitochondrial reactive oxygen species (mtROS) levels, MitoSOX Red dye (Invitrogen) was utilized following the manufacturer’s protocol. The experiment hole is seeded with 1.6 × 10^4^ cells per slide, and after seeding, the cells are left to adhere to the wall. Mitochondrial superoxide levels were assessed using MitoSOX Red dye. HUVECs were seeded in 24-well plates and incubated with 5 μM MitoSOX Red working solution. Following staining, cells were fixed and analyzed using a fluorescence microscope. Fluorescence intensity was quantified using ImageJ software. For each experimental condition, at least three random fields of view were selected. Mean fluorescence intensity was measured in each selected field.

### Assessment of mitochondrial permeability transition pore (mPTP) opening

The degree of mPTP opening was assessed using the mPTP Assay Kit (Beyotime) according to the manufacturer's instructions. Cells were seeded in 24-well plates and treated according to the experimental design. The experiment hole is seeded with 8 × 10^3^ cells per slide, and after seeding, the cells are left to adhere to the wall. An appropriate volume of Calcein AM staining solution, fluorescence quenching working solution, or Ionomycin control was added to the wells. The plate was then incubated at 37 °C in the dark for 30 min. After the incubation, the medium was replaced with fresh prewarmed culture medium. The cells were further incubated at 37 °C in the dark for another 30 min to ensure sufficient hydrolysis of Calcein AM by intracellular esterases to produce Calcein. The stained cells were observed and imaged under a fluorescence microscope for analysis. ImageJ software was used for fluorescence intensity quantification. For each experimental condition, at least three random fields of view were selected. Mean fluorescence intensity was measured in each selected field.

### Assessment of mitochondrial membrane potential (MMP)

The strength of the mitochondrial membrane potential (MMP) was evaluated using the JC-1 Assay Kit (Beyotime) according to the manufacturer’s instructions. The experiment hole is seeded with 1 × 10^4^ cells per slide, and after seeding, the cells are left to adhere to the wall. JC-1 staining working solution (1 mL) was added to each well, and the solution was mixed thoroughly. The cells were incubated at 37 °C for 20 min in a cell incubator. Then the cells were washed twice with JC-1 staining buffer. Next, 2 mL of cell culture medium was added to each well. The cells were then observed under a fluorescence microscope or laser confocal microscope. Depolarized mitochondria (JC-1 monomers) emit green fluorescence (excitation at 490 nm, emission at 530 nm). Polarized mitochondria (JC-1 aggregates) emit red fluorescence (excitation at 525 nm, emission at 590 nm). The ratio of red to green fluorescence intensity indicates the status of MMP in the cells, with a decrease in the red/green ratio indicating mitochondrial depolarization. ImageJ software was used for fluorescence intensity quantification. For each experimental condition, at least three random fields of view were selected. Mean fluorescence intensity was measured in each selected field.

### Detection of mtDNA and cytosolic DNA by staining

Immunofluorescence double staining was employed to assess the release of mitochondrial DNA (mtDNA) into the cytoplasm. The experiment hole is seeded with 1 × 10^4^ cells per slide, and after seeding, the cells are left to adhere to the wall. The prewarmed (37 °C) staining solution containing MitoTracker Red was added to the cells in a 24-well plate at a final concentration of 400 nM. The cells were then incubated for 15–45 min in the dark to allow for mitochondrial staining with MitoTracker Red. Then, an equal volume (1.0 mL) of Quant-iT™ PicoGreen dsDNA Reagent Working Water Solution was added to each cell sample. The samples were incubated for 10 min at 37 °C in the dark to allow for DNA staining with PicoGreen Reagent. Following the incubation, the PicoGreen Reagent was discarded, and the samples were sealed with Hoechst-containing Protest Fluorescence Quenching Blocking Solution (Beyotime). The stained cells were observed using a Fluorescence confocal microscope (ZEISS LSM 900) to visualize the fluorescence signals. Excitation/emission peaks of approximately 579/599 nm were used for MitoTracker Red. Excitation/emission peaks of approximately 502/523 nm were used for the Quant-iT™ PicoGreen dsDNA Reagent.

### DNA isolation and mtDNA copy number analysis

The cytoplasm of treated HUVECs was extracted using a mitochondrial isolation kit (Beyotime). The cell pellet was gently resuspended in ice-cold PBS. Cells were then precipitated by centrifugation at 4 °C for 5 min. The supernatant was carefully discarded. Next, 1–2.5 mL of mitochondrial isolation reagent was added to the cell pellet. The cells were gently suspended and left in an ice bath for 10–15 min. Afterward, the cells were homogenized with 10–30 strokes. The homogenized mixture was then centrifuged at 600 × g for 10 min at 4 °C. The resulting supernatants were carefully transferred to another centrifuge tube. This supernatant was then centrifuged at 11,000 g at 4 °C for 10 min. The collected supernatants were subjected to a final centrifugation step at 12,000 × g for 10 min at 4 °C. The supernatants obtained from the final centrifugation were collected and considered as the cytosolic fraction, from which mitochondria had been removed. DNA was isolated from the collected cytoplasmic fractions using a Genomic DNA Micro Extraction Kit (Axygen) following the manufacturer’s instructions. Mitochondrial DNA (mtDNA) and nuclear DNA (nDNA) were separately analyzed. For mtDNA copy number analysis: ND1 or COX1 and 18S genes were amplified using previously described methods [17]. 10 ng of DNA was used for real-time PCR on a CFX96 system (Bio-Rad) using ChamQ SYBR qPCR Master Mix (Vazyme). The mtDNA/nDNA ratio was calculated to determine the mtDNA copy number. All the primers used for real-time PCR are listed in Tables 1 and 2.

### Statistical analysis

Data are presented as the mean ± standard error of the mean (SEM) or mean ± standard deviation (SD) from at least three independent experiments. Statistical analyses were performed using Prism 9.0 software (GraphPad, USA). A t-test was used for two-group comparisons, and one-way analysis of variance (ANOVA) with post-hoc tests was applied for multiple-group comparisons. A p-value of less than 0.05 (*p* < 0.05) was considered statistically significant.

## Results

### Enhanced mtDNA release and elevated cGAS/STING pathway activation in KD patients

**Fig. 1:**
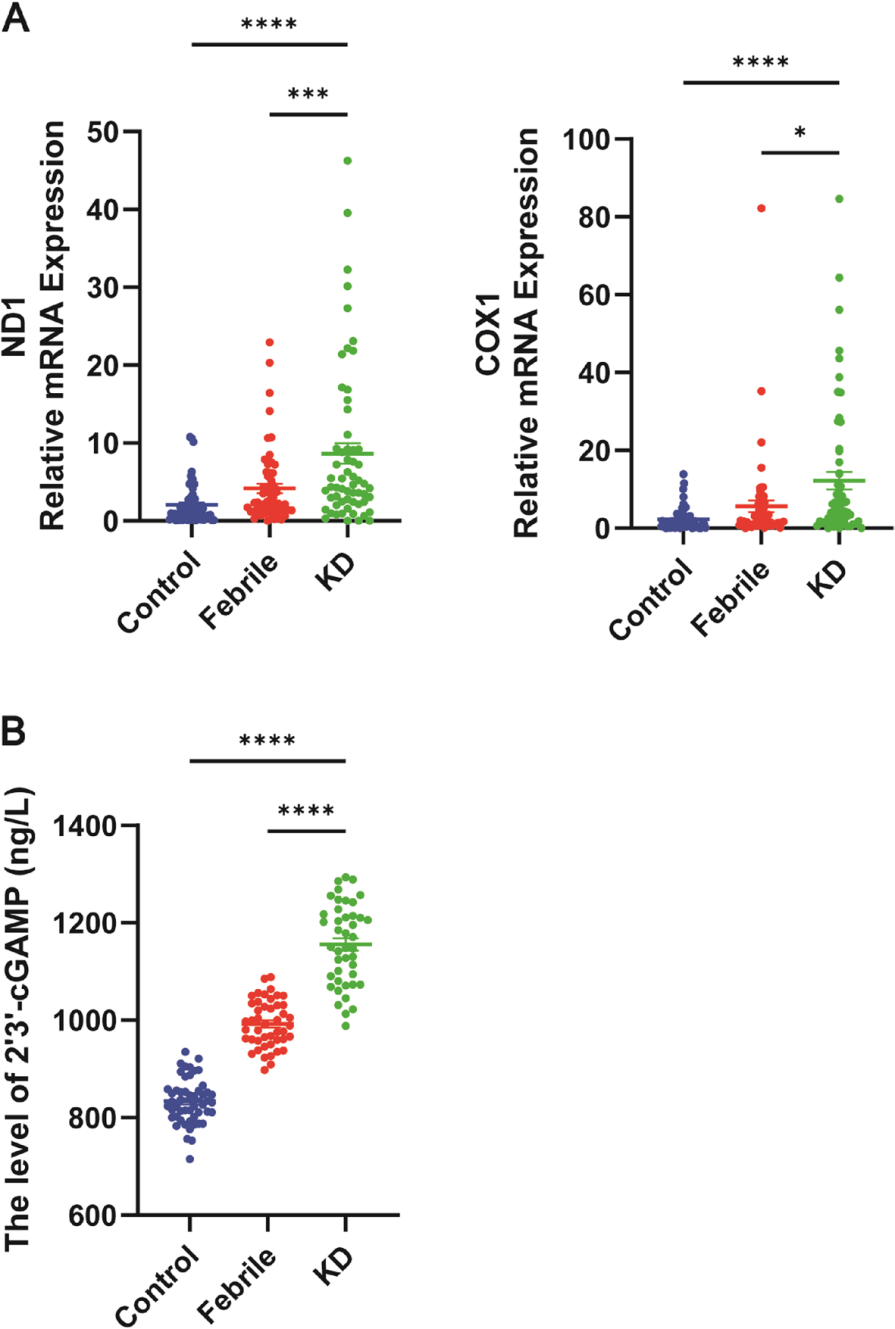
Enhanced mtDNA Release and Elevated cGAS/STING Pathway Activation in KD Patients. **A** Quantification of peripheral blood free mitochondrial DNA was performed using qRT-PCR in the control, febrile, and Kawasaki disease (KD) groups (*n* = 60). **B** Expression levels of peripheral blood serum 2’3’-cGAMP were analyzed by ELISA among the different groups (*n* = 51). The data, shown as mean ± SEM from at least three independent experiments. Statistical analysis was performed using a t-test between two groups. Asterisks denote statistical significance: *P < 0.05, **P < 0.01, ***P < 0.001, ****P < 0.0001

Peripheral blood samples from KD patients demonstrated a significant increase in mtDNA release, a key mediator of inflammatory responses [18]. Quantitative RT-PCR analysis revealed elevated levels of mtDNA-specific genes, mt-ND1 and COX1, in KD patients compared to both control and febrile groups (Fig. 1A). This finding is consistent with previous reports highlighting mitochondrial dysfunction in KD [8]. Concurrently, ELISA measurements indicated a marked increase in the levels of 2′3'-cGAMP, a signaling molecule downstream of cGAS activation [19], in KD patient serum (Fig. 1B). These results suggest a potential link between mitochondrial damage, mtDNA release, and the activation of the cGAS-STING signaling pathway in KD pathogenesis.

### Mitochondrial damage and mtDNA release in KD animal models

**Fig. 2:**
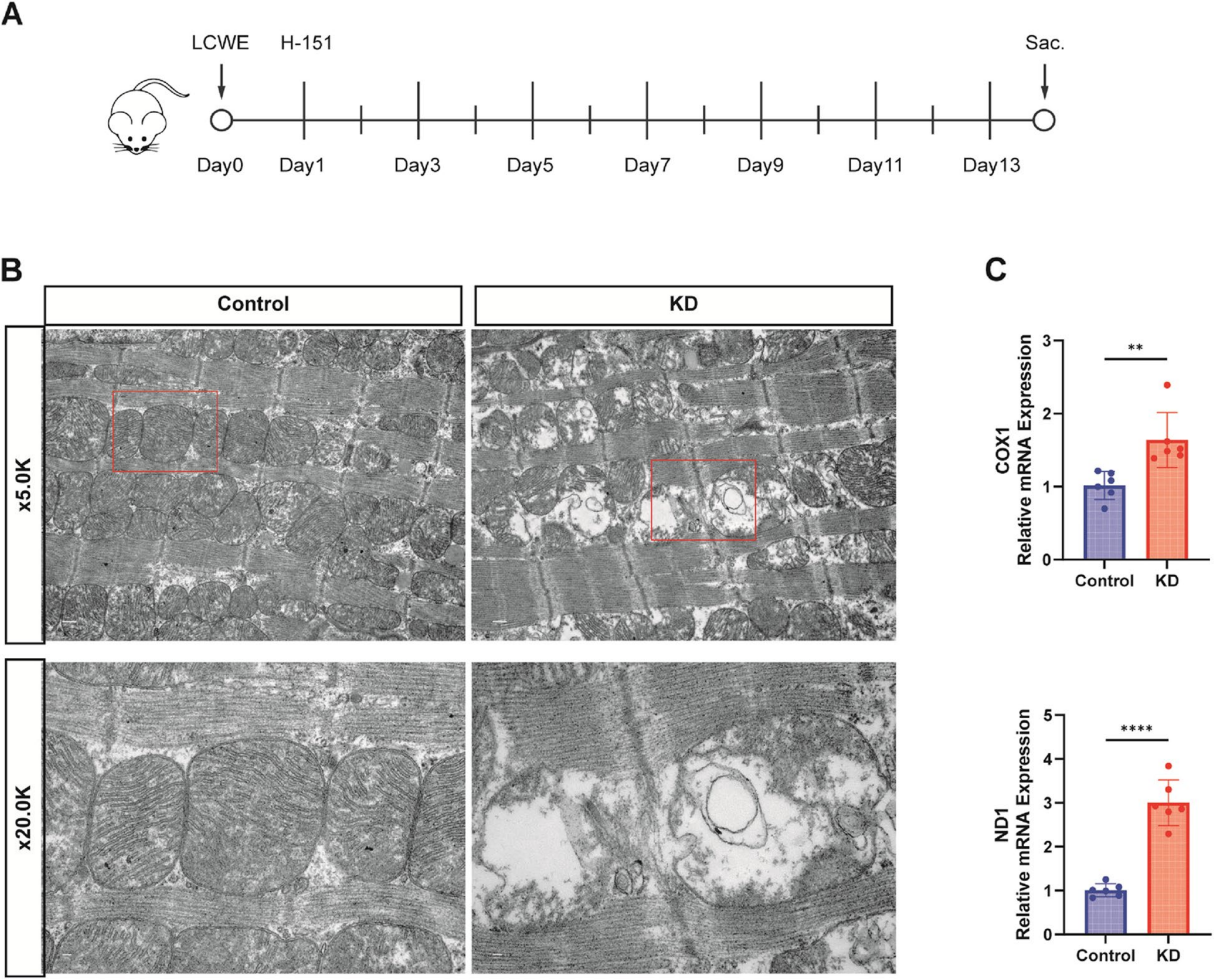
Mitochondrial Damage and mtDNA Release in KD Animal Models. Control group: injected with equal volume of PBS, KD group: injected with LCWE, H-151 group: injected with H-151 (7 mg/kg) every other day after LCWE injection according to the modeling protocol. **A** Example of the modeling timeline of the mouse model simulating Kawasaki disease immune vasculitis. **B** Representative images of cardiac mitochondrial structure and morphology in control and KD mice observed under transmission electron microscopy (*n* = 3). Scale bar = 2.0 µm/500 nm. **C** RT-qPCR to detect the expression of peripheral blood plasma free mitochondrial DNA specific genes ND1 and COX1 mRNA in mice of the ccontrol and KD groups (*n* = 6)

To explore mitochondrial damage and mtDNA release in vivo, we employed a murine model of KD induced by Lactobacillus casei cell wall extract (LCWE) [20]. TEM analysis of cardiac tissues from KD mice exhibited pronounced mitochondrial alterations, including vacuolation and cristae disarray (Fig. 2B). Quantitative RT-qPCR confirmed increased levels of circulating mtDNA, as indicated by the elevated expression of ND1 and COX1 genes in the peripheral blood of KD mice (Fig. 2C).

### Activation of the cGAS-STING pathway in KD animal models

**Fig. 3:**
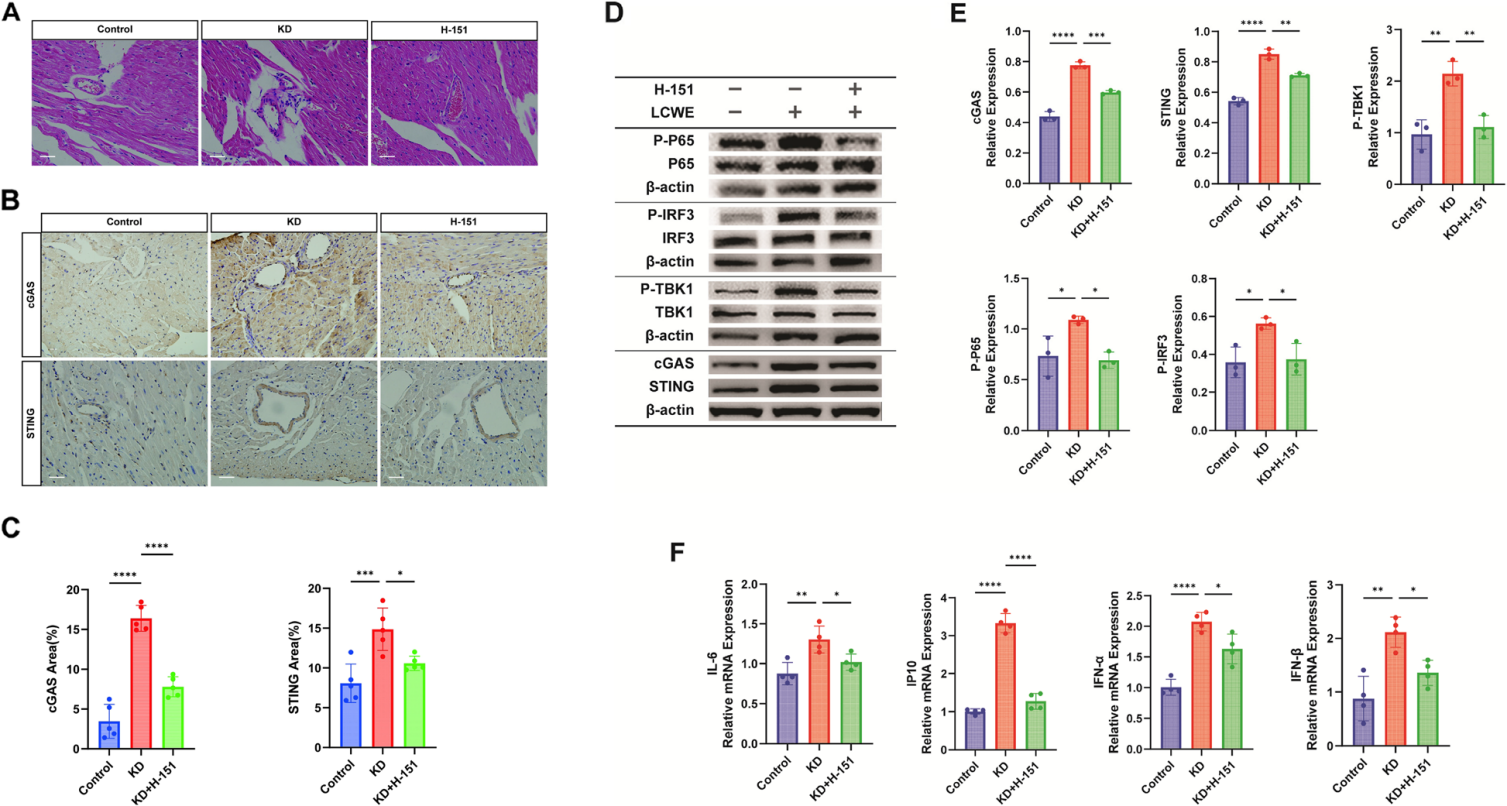
Activation of the cGAS-STING Pathway in KD Animal Models. **A** HE staining to detect inflammatory infiltration of coronary arteries in mice of the Control, KD, and H-151 treatment groups (*n* = 5). Scale bar = 10 µm. **B** Representative images of immunohistochemistry showing expression of cGAS and STING in mice heart tissues (*n* = 5). Scale bar = 10 µm. **C** Relative quantitative statistics of the immunohistochemistry-positive area of cGAS and STING (*n* = 5). **D** Western Blot to detect the expression of key proteins of the cGAS-STING signaling pathway: cGAS, STING, P-TBK1, P-P65, P-IRF3 (*n* = 3) in mice heart tissues. **E** Quantitative statistical plots of relevant proteins detected by Western Blot (*n* = 3). **F** RT-qPCR to detect the expression of inflammatory factors downstream of the pathway: IL-6, IP10, IFNα, IFNβ mRNA (*n* = 4). T-test was used for data analysis between two groups, and one-way analysis of variance was used for data analysis of more than two groups. Data are shown as mean ± SD of at least three independent experiments. *P* values are indicated by asterisks: *P < 0.05, **P < 0.01, ***P < 0.001, ****P < 0.0001

The impact of cGAS-STING signaling on KD-associated inflammation was assessed using H-151, a specific STING antagonist. KD mice treated with H-151 showed reduced coronary vascular inflammation and decreased expression of cGAS and STING in heart tissues (Fig. 3A-C). Western blot analysis further confirmed increased expression of cGAS, STING, and phosphorylated forms of TBK1, P65, and IRF3 in KD mice, along with elevated levels of inflammatory cytokines IL-6, IP10, IFNα, and IFNβ (Fig. 3D-F). H-151 treatment mitigated these effects, suggesting that mitochondrial damage triggers mtDNA release and may subsequent activation of the cGAS-STING pathway, leading to inflammation in KD.

### Mitochondrial dysfunction and mtDNA release in KD cell models

The activation of the cGAS-STING pathway in clinical samples and Kawasaki Disease (KD) animal models has provided significant insights. To delve into the specific mechanisms underlying this pathway's activation, we focused on its relationship with mitochondrial dysfunction, the primary cause of potential mitochondrial DNA (mtDNA) release into the cytoplasm. We established a KD cell model using Human Umbilical Vein Endothelial Cells (HUVECs) cocultured with serum from children with KD [21].

**Fig. 4:**
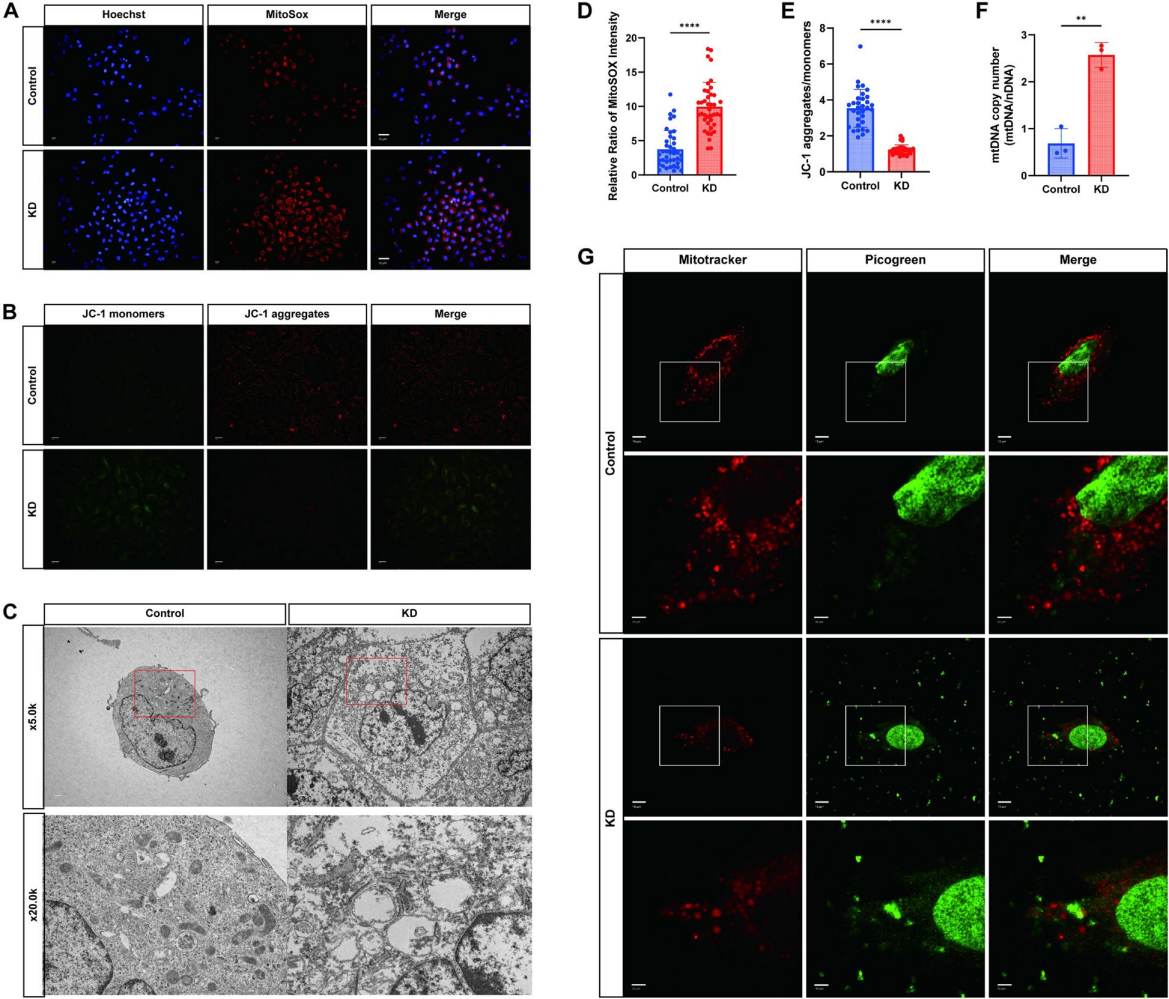
Mitochondrial Dysfunction and mtDNA Release in KD Cell Models. **A** Representative fluorescence images showing mitochondrial superoxide detected by MitoSOX Red staining among different groups (*n* = 40). Scale bar = 10 µm. **B** Representative fluorescence images illustrating mitochondrial membrane potential stained with the JC-1 kit and observed via fluorescence microscopy for mitochondrial monomers and multimers (*n* = 32). Scale bar = 10 µm. **C** Representative images displaying the morphology and structure of mitochondria in cells from different groups observed under a transmission electron microscope (*n* = 3). Scale bar = 1.0/5.0 µm. **D** Bar graph depicting the quantification of MitoSOX signal intensity fluorescence (*n* = 40). **E** Bar graph representing the ratio of JC-1 monomer to multimer (*n* = 32). **F** Cytoplasmic DNA extracts without mitochondria were used to calculate the mitochondrial copy number of the various treatment groups via RT-qPCR (*n* = 3). **G** Mitotracker labeling of mitochondria and PicoGreen labeling of DNA were performed and observed under confocal microscopy in different treatment groups. Mitotracker labeled mitochondria, PicoGreen labeled DNA, and focusing microscopy were used to observe DNA not colocalizing with nuclei and mitochondria (*n* = 3). (Red: mitotracker, Green: mtDNA). Scale bar = 10/30 µm. T-test was utilized for data analysis between two groups. Data are presented as mean ± SD of at least three independent experiments. *P*-values are denoted by asterisks: *P < 0.05, **P < 0.01, ***P < 0.001, ****P < 0.0001

Given that numerous studies highlight oxidative stress (OS) as a pivotal mechanism leading to mitochondrial damage [22], we used MitoSOX Red staining to assess mitochondrial Reactive Oxygen Species (mtROS) levels in KD cell models to investigate mitochondrial function. Following coculture of HUVECs with serum from KD patients, mtROS production increased (Figs. 4A and 3D), indicating enhanced mitochondrial stress. Moreover, a reduction in the Mitochondrial Membrane Potential (MMP) is a key event in early apoptosis [23]. The decline in MMP is evident through the shift in the JC-1 signal from red to green fluorescence; after JC-1 staining, the KD group showed an increase in JC-1 monomers and a decrease in MMP (Fig. 4B, E), indirectly indicating impaired mitochondrial function.

For a more intuitive observation of mitochondrial structural abnormalities, we examined differences in mitochondrial morphology and structure among the groups using Transmission Electron Microscopy (TEM). In the KD group, mitochondrial cristae were absent, appearing swollen with noticeable vacuole-like changes (Fig. 4C), indicating conditions conducive to mtDNA release into the cytoplasm.

To further observe mtDNA release into the cytoplasm, we labeled DNA structures with PicoGreen staining and mitochondria with MitoTracker. This revealed that some double-stranded DNA (dsDNA) colocalized with the nucleus, some with mitochondria, and some translocated into the cytoplasm (Fig. 4G). To confirm cytoplasmic mtDNA release, we quantified cytoplasmic mtDNA in HUVECs cocultured with or without KD patient serum, following the methods of Zhou L et al. [24]. The results demonstrated a significant enrichment of cytoplasmic mtDNA in HUVECs after coculture with KD patient serum (Fig. 4F).

### Activation of the cGAS-STING *axis* in KD cell models

To further delineate the role of the cGAS-STING pathway in KD cell models, we established these models as described previously [21]. Additionally, a KD treatment model was created by pretreating cells with the pathway inhibitor H-151. Human Umbilical Vein Endothelial Cells (HUVECs) were stimulated with various concentrations of H-151, and optimal cell viability was observed at 1.5 μM H-151. Henceforth, we maintained the use of 1.5 μM H-151 as the optimal concentration for our experiments.

**Fig. 5:**
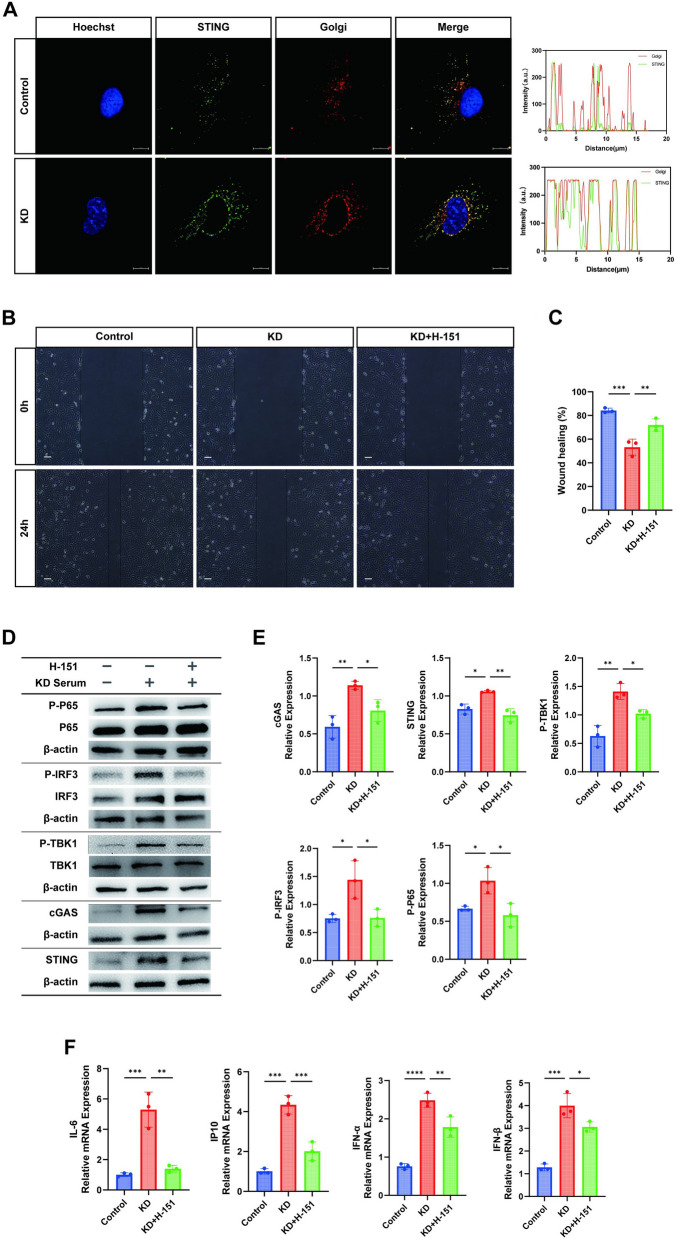
Activation of the cGAS-STING Axis in KD Cell Models. Control group: No special treatment, KD group: HUVECs co-cultured with KD inactivated serum for 24 h, H-151 group: HUVECs pre-treated with H-151 (1.5 µM) for 1 h, then co-cultured with KD inactivated serum for 24 h. **A** Confocal microscopy observation representative images depicting the colocalization of STING and Golgi apparatus with quantitative analysis of visualized co-localization. Scale bar = 10 µm. **B**-**C** Cell scratch assay for statistical comparison of cell migration ability and mobility among different groups (*n* = 3). Scale bar = 10 µm. **D**-**E** Western blotting for detection of cGAS-STING pathway-associated proteins (cGAS, STING, P-TBK1, P-IRF3, P-P65) in the control, KD, and H-151 groups (*n* = 3), with quantification of protein expression levels and grey values. **F** RT-qPCR to detect the expression of downstream inflammatory factors IL-6, IP10, IFNα and IFNβ (*n* = 3) mRNAs among the three groups. One-way analysis of variance was used for data analysis of more than two groups. Data are presented as mean ± SD from at least three independent experiments. *p*-values are denoted by asterisks: *P < 0.05, **P < 0.01, ***P < 0.001, ****P < 0.0001

Activation of the cGAS-STING pathway is often accompanied by changes in subcellular organelles. Under normal conditions, STING localizes to the Endoplasmic Reticulum (ER) and upon activation, translocates to the Golgi, where it binds to TANK-Binding Kinase 1 (TBK1) to form a signaling body crucial for downstream responses [25]. Thus, we employed GM130 to label the Golgi apparatus in co-immunofluorescence assays with STING to further visualize its activation. Our results revealed that in the control group, STING weakly colocalized with the Golgi apparatus, whereas colocalization was significantly enhanced in the KD group (Fig. 5A). The graphical representation of colocalization is displayed on the right. Having confirmed the activation status in the STING disease model group, we assessed cell function through a wound healing assay, where cell migration was notably restored following H-151 treatment compared to the KD group (Fig. 5B-C).

Western blot analysis demonstrated increased expression of cGAS, STING, and downstream signaling proteins in KD cells, which was attenuated with H-151 treatment (Fig. 5D-E). Correspondingly, RT-qPCR analysis showed decreased expression of inflammatory factors (Fig. 5F).

### Inhibition of cGAS suppresses inflammatory responses in KD cell models

**Fig. 6:**
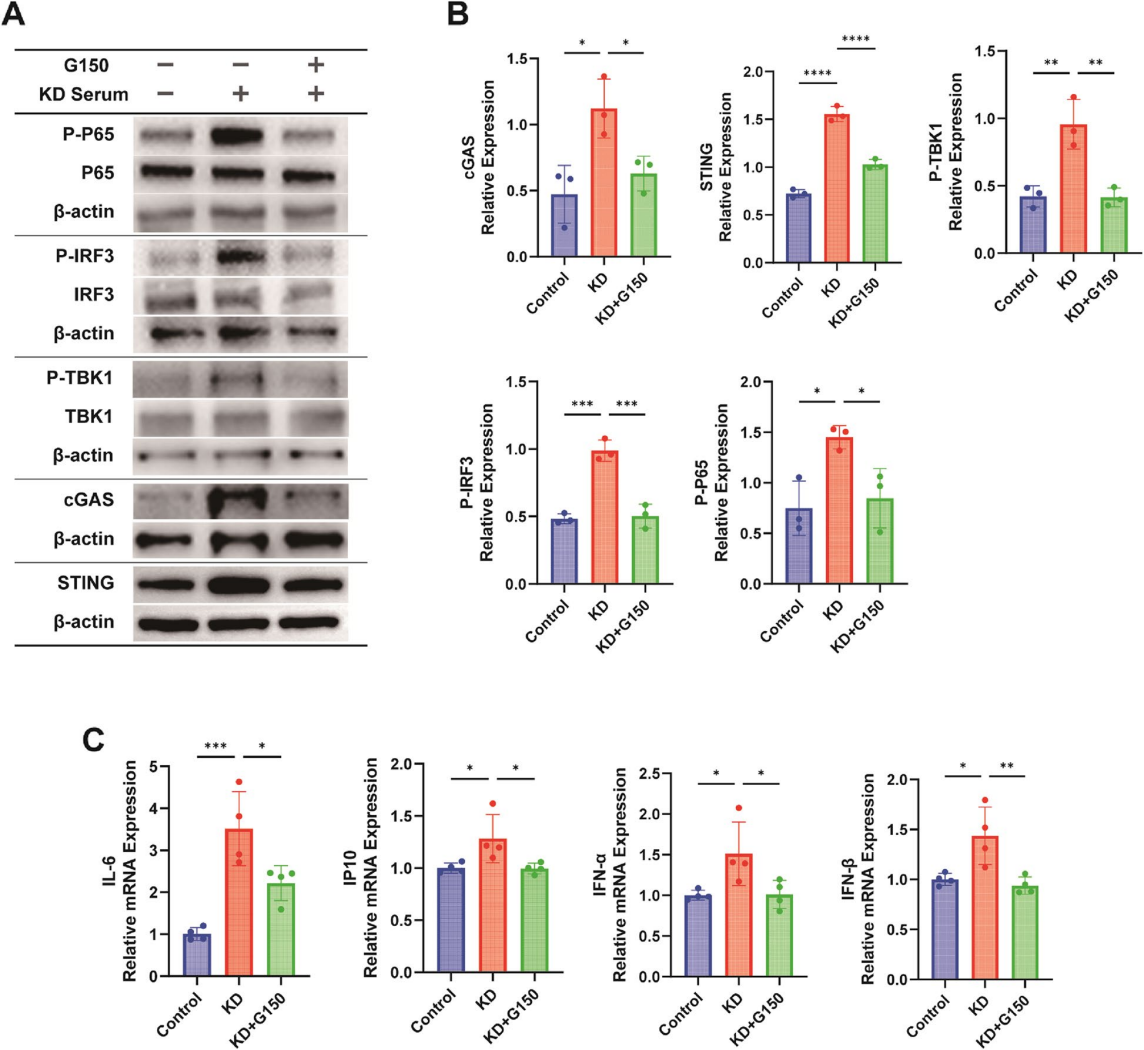
Inhibition of cGAS Suppresses Inflammatory Responses in KD Cell Models. Control group: No special treatment, KD group: HUVECs co-cultured with KD inactivated serum for 24 h, G150 group: HUVECs pre-treated with G150 (5 µM) for 1 h, then co-cultured with KD inactivated serum for 24 h. **A**-**B** Western blot (WB) was used to detect the protein molecules and grayscale quantification of the cGAS-STING signaling pathway after the addition of G150 (*n* = 3). **C** RT-qPCR was used to detect the relative expression levels of inflammatory factor mRNA downstream of the cGAS-STING signaling pathway after the addition of G150 (*n* = 4). One-way analysis of variance was used for data analysis of more than two groups. Data are presented as mean ± SD from at least three independent experiments. *p*-values are denoted by asterisks: *P < 0.05, **P < 0.01, ***P < 0.001, ****P < 0.0001

After applying the STING inhibitor, we further used a cGAS inhibitor to repeat the validation of inhibiting this pathway in a Kawasaki disease cell model. We used 5 µM G150 intervention on cells according to the literature to achieve inhibition of the cGAS-STING signaling pathway [26]. G150 is an effective and highly selective cGAS inhibitor. After adding G150 pretreatment, the cGAS-STING pathway was inhibited at the protein level and the mRNA level of inflammatory factors (Fig. 6A-C).

### Contribution of mPTP opening and TFAM downregulation to cytoplasmic mtDNA accumulation

**Fig. 7:**
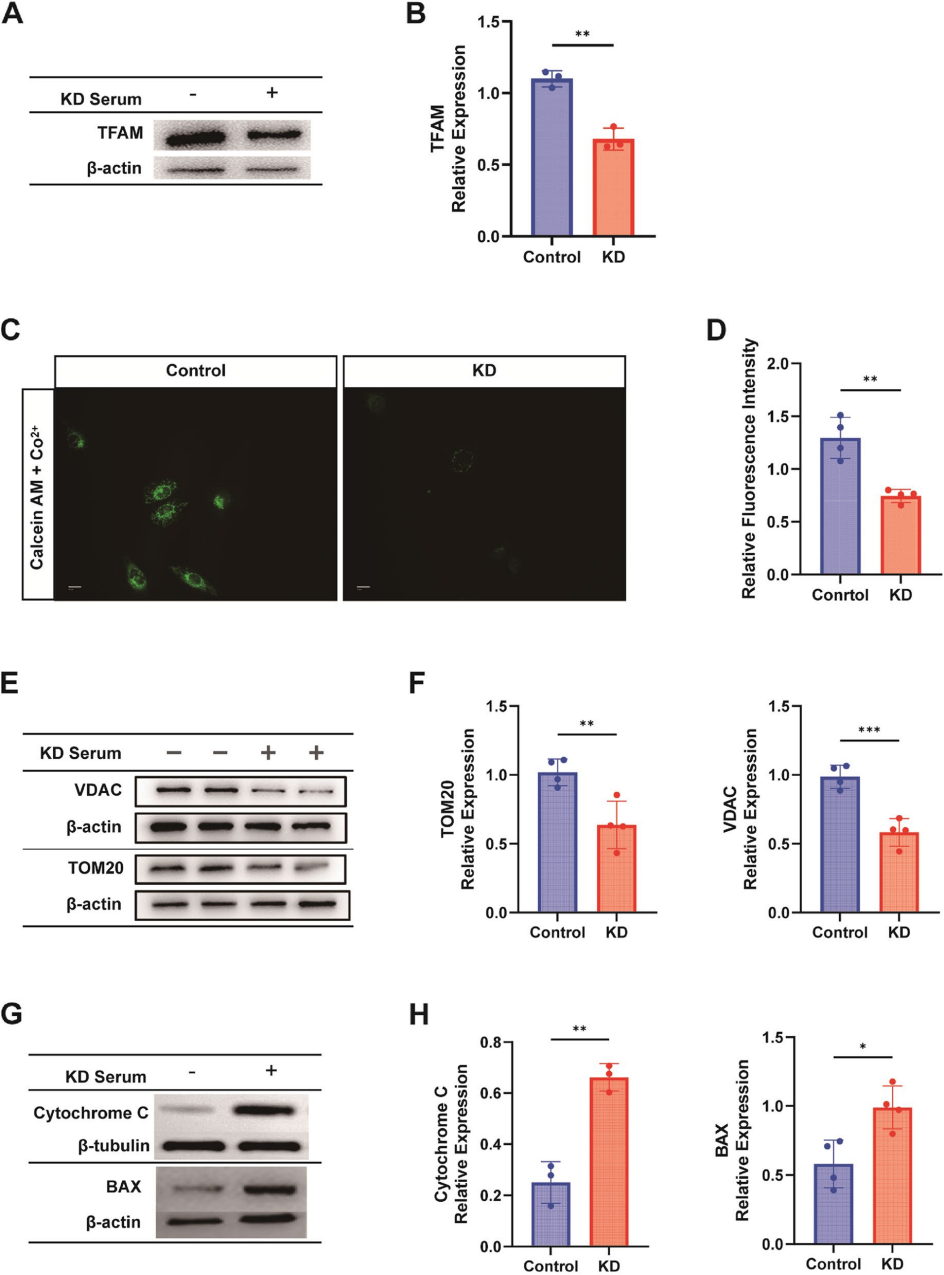
Contribution of mPTP Opening and TFAM Downregulation to Cytoplasmic mtDNA Accumulation. **A** Western blot depicting the protein expression level of TFAM among different groups (*n* = 3). **B** Bar graph illustrating the quantification of grey values of TFAM in different groups (*n* = 3). **C**-**D** mPTP Opening Level Detection: Calciumxanthophyll AM/Co2 + quenching staining represented in fluorescence images for quantitative analysis of fluorescence intensity(*n* = 4). Scale bar = 10 µm. **E**–**F** Western blot assay for VDAC (*n* = 4) and TOMM20 (*n* = 4) expression, along with a histogram for quantification of grey scale values. **G**-**H** Western blot assay for BAX (*n* = 4) and Cytochrome C (*n* = 3) expression, along with a histogram for quantification of grey scale values. T-test was utilized for data analysis between two groups. Data are presented as mean ± SD from at least three independent experiments. *p*-values are denoted by asterisks: *P < 0.05, **P < 0.01, ***P < 0.001, ****P < 0.0001

The accumulation of mtDNA in the cytoplasm prompted an investigation into the underlying causes. Western blot analysis showed decreased expression of TFAM, a mitochondrial transcription factor critical for mtDNA stability [27] (Fig. 7A-B).

In addition to decreased mtDNA stability, another critical factor for mtDNA release into the cytoplasm is the opening of the mitochondrial permeability transition pore (mPTP). The mPTP, a large channel between the inner and outer mitochondrial membranes, regulates the influx and efflux of substances, including mtDNA [28]. OS promotes the opening of mPTP [29]. Therefore, we have assessed the opening of the mitochondrial permeability transition pore (mPTP), revealing increased mPTP opening in KD cells (Fig. 7C-D).

MtDNA not only leaks through the mitochondrial permeability transition pore (mPTP) but also needs to pass through the mitochondrial outer membrane. Therefore, we verified the downregulation of VDAC [30] and TOM20, which as mitochondrial outer membrane proteins (Fig. 7E-F). Additionally, BAX pores refer to the channels formed by the BAX protein during the process of cell apoptosis. There, the BAX protein or mPTP changes the permeability of the mitochondrial membrane by forming pores, leading to the release of some proteins from the cell interior, such as cytochrome C and apoptosis-inducing factor. Therefore, we extracted cytosolic fractions from cells lacking mitochondria for validation. The Western Blot results showed an increased release of cytochrome C. And the expression of BAX also increases after co-culturing with KD-inactivated serum (Fig. 7G-H). These results suggest that in the Kawasaki disease group, the opening of mPTP and the degradation of the mitochondrial outer membrane create conditions for the subsequent release of mtDNA into the cytosol.

### Pharmacological inhibition of mPTP reduces cytoplasmic mtDNA accumulation and improves mitochondrial function

**Fig. 8:**
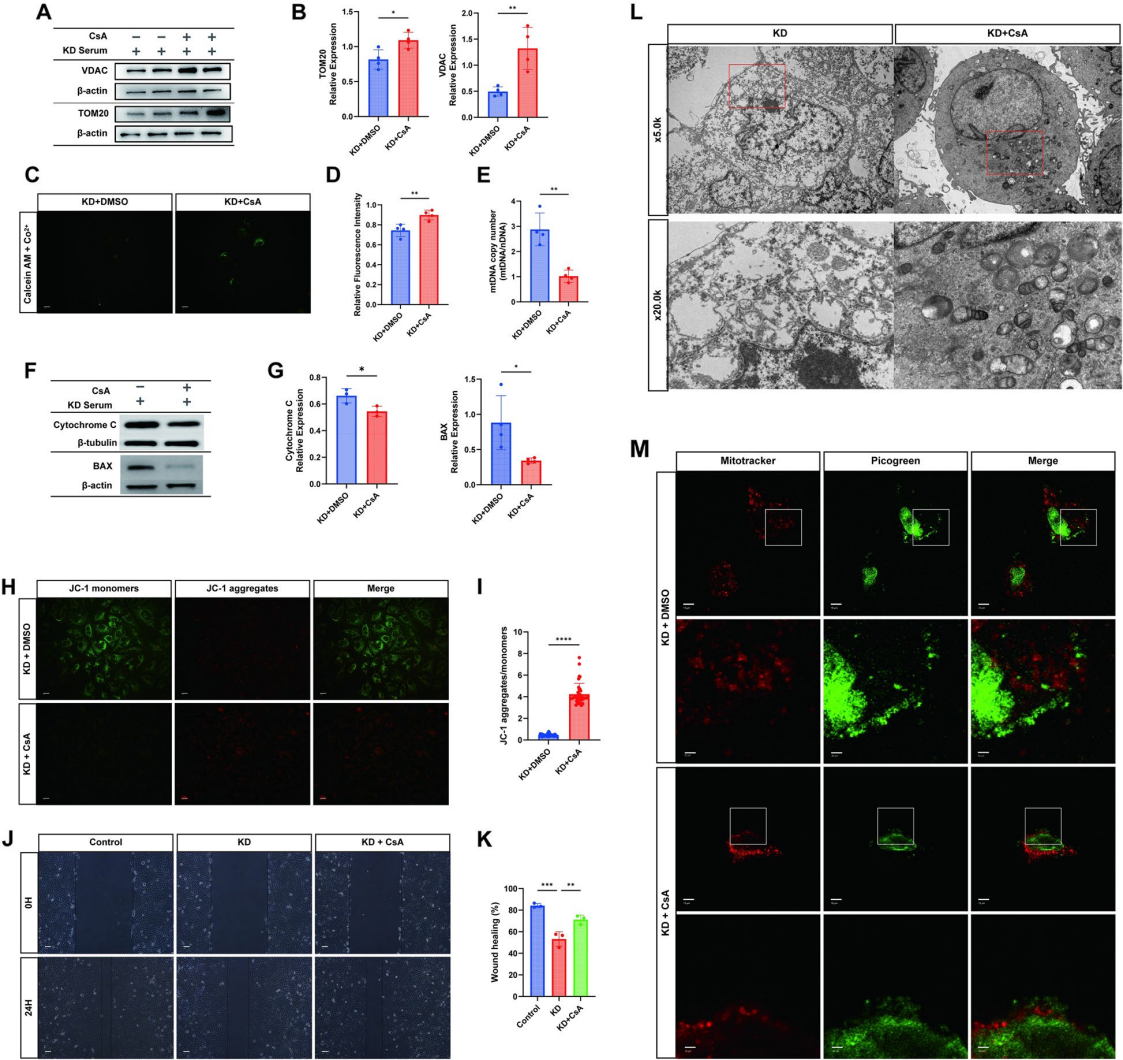
Pharmacological Inhibition of mPTP Reduces Cytoplasmic Mitochondrial DNA Accumulation and Improves Mitochondrial Function. Control Group: No special treatment, KD Group: HUVECs co-cultivated with KD inactivated serum for 24 h, CsA Group: HUVECs pre-treated with CsA (10 µM) for 1 h, followed by co-cultivation with KD inactivated serum for 24 h. **A** Western blot assay for VDAC (*n* = 4) and TOMM20 (*n* = 4) expression. **B** Quantification of grey values for TOM20 and VDAC (*n* = 4). **C** Representative fluorescence images of calcium xanthophyll AM/Co2 + quenching staining in Control and KD groups (*n* = 4). Scale bar = 10 µm. **D** Quantification of fluorescence intensity of the open level of mPTP in different treatments (*n* = 4). **E** Cytoplasmic DNA extracts without mitochondria used for calculating mitochondrial copy number in different treatment groups by qRT-PCR (*n* = 4). **F**-**G** Western blot assay for BAX (*n* = 4) and Cytochrome C (*n* = 3) expression, along with a histogram for quantification of grey scale values. **H**-**I** JC-1 kit staining of mitochondrial membrane potential and observation of mitochondrial monomers under a fluorescence microscope in different groups. Bar graphs represent the ratio of JC-1 monomers to multimers (*n* = 37). Scale bar = 10 µm. **J**-**K** Cellular scratch assay to detect cell migration ability between different groups. Bar graphs represent the rate of healing (*n* = 3). Scale bar = 10 µm. **L** Representative images of cellular mitochondrial morphology and structure under a transmission electron microscope (*n* = 3). Scale bar = 1.0/5.0 µm. **M** Confocal microscopy images of Mitotracker-labeled mitochondria, picogreen-labeled DNA, and DNA not colocalized with nuclei and mitochondria in different groups (*n* = 3). (Red: mitotracker, Green: mtDNA). Scale bar = 10 µm. Data analysis: T test for comparisons between two groups. One-way analysis of variance for comparisons of more than two groups. Data shown as mean ± SD of at least three independent experiments. *p* values indicated by asterisks: *P < 0.05, **P < 0.01, ***P < 0.001, ****P < 0.0001

HUVECs were pretreated with cyclosporine (CsA), a drug known to inhibit the opening of the mitochondrial permeability transition pore (mPTP), for 1 h prior to experimentation [31]. Through CCK-8 assays, the optimal concentration of CsA was determined to be 10 μM. Compared to the KD group, HUVECs pretreated with 10 μM CsA showed reduced entry of cobalt chloride into the cytoplasm, decreased calreticulin quenching, and lowered mPTP opening (Fig. 8C, D). Additionally, the expression mitochondrial outer membrane protein TOM20 and VDAC was restored in CsA-pretreated cells (Fig. 8A, B) and the release of cytochrome C and BAX are also decreased (Fig. 8F-G).

Mitochondrial structure and cellular function were further assessed through JC-1 staining of the mitochondrial membrane potential (MMP), wound healing assays, and transmission electron microscopy (TEM) for observing mitochondrial structure. CsA treatment led to restoration of MMP (Fig. 8H-I) and improved cellular function, as evidenced by enhanced wound healing compared to the untreated group (Fig. 8J-K). Although mitochondrial structure remained impaired in the CsA group, it showed amelioration compared to the untreated group (Fig. 8L).

Moreover, to confirm the closure of the mPTP release channel and the reduction in mtDNA release due to CsA, double fluorescence staining was performed on CsA-pretreated and untreated cells. Confocal microscopy revealed reduced cytoplasmic mtDNA accumulation in CsA-treated cells after coculture with KD patient serum, in contrast to untreated cells (Fig. 8M). These findings were supported by cytoplasmic mtDNA quantification, confirming a decrease in mtDNA copy number following CsA intervention (Fig. 8E).

### Reduced mtDNA release attenuates inflammatory responses by inhibiting cGAS-STING activation

**Fig. 9:**
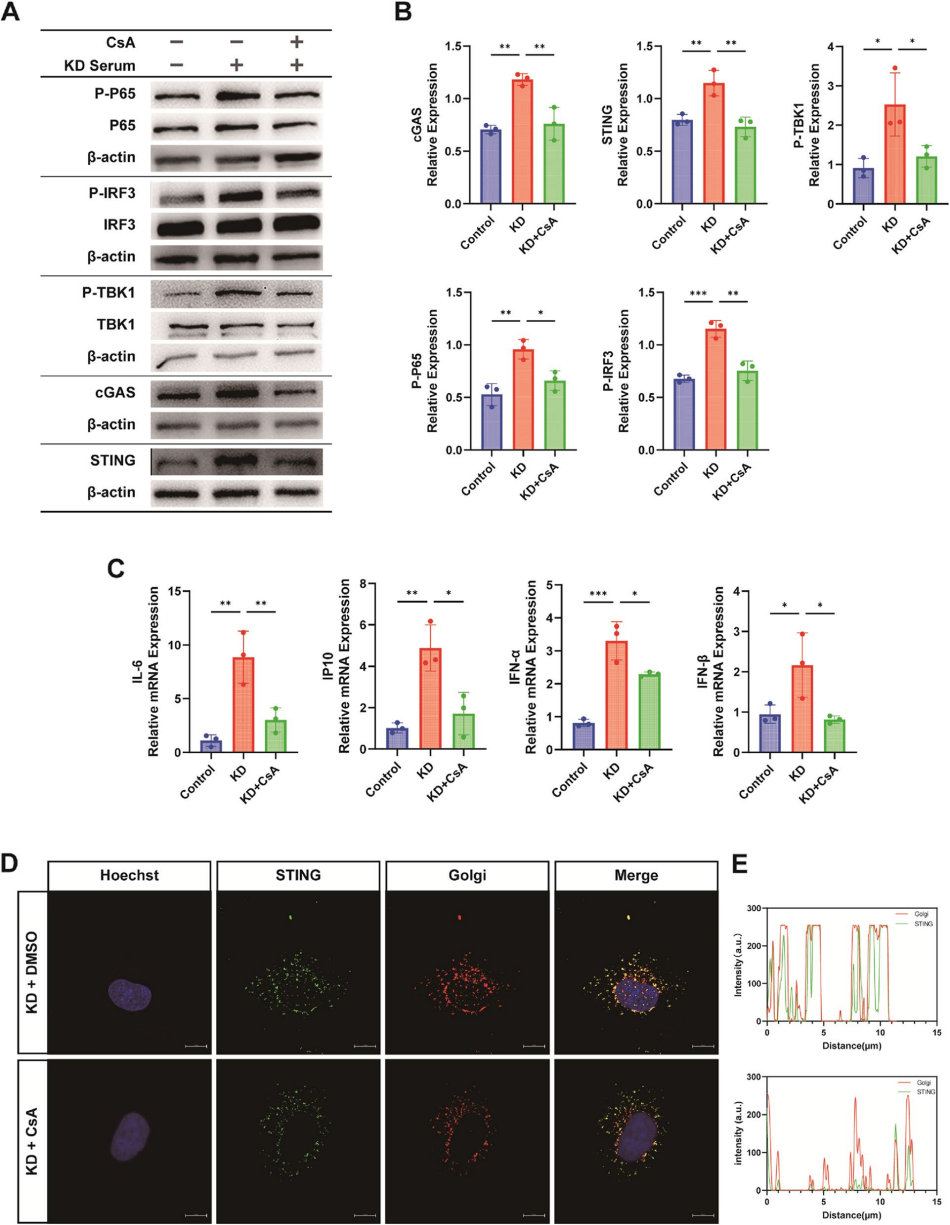
Reduced mtDNA Release Attenuates Inflammatory Responses by Inhibiting cGAS-STING Activation. **A**-**B** Western blot analysis of cGAS-STING pathway-related proteins: cGAS, STING, P-TBK1, P-IRF3, and P-P65 (*n* = 3). **C** RT-qPCR quantification of downstream inflammatory factors IL-6, IP10, IFNα, IFNβ mRNA expression among the three groups (*n* = 3). **D**-**E** Representative images and visual co-localization analysis of STING colocalization with the Golgi apparatus observed under confocal microscopy. Scale bar = 10 µm. Data analysis: One-way analysis of variance used for data analysis of more than two groups. Data presented as mean ± SD of at least three independent experiments. *p*-values indicated by asterisks: *P < 0.05, **P < 0.01, ***P < 0.001, ****P < 0.0001

Previous experiments have demonstrated that inhibiting mPTP opening leads to reduced mtDNA release, thereby enhancing mitochondrial function. We further investigated whether this reduction in mtDNA release could inhibit the activation of its ligand cGAS, consequently mitigating the inflammatory response. The expression of cGAS-STING pathway-related proteins and downstream inflammatory factors was decreased with CsA treatment, as shown by Western blot and RT-qPCR analyses (Fig. 9A-C). Confocal microscopy revealed reduced colocalization of STING with the Golgi apparatus following CsA treatment, indicating suppressed pathway activation (Fig. 9D-E).

## Discussion

In this study, we have observed that the mitochondrial structures of myocardial tissues and endothelial cells are impaired in both in vivo and in vitro KD models, resulting in cytoplasmic DNA leakage and the activation of the cGAS-STING signaling pathway. In endothelial cells co-cultured with serum from Kawasaki disease patients, the formation of a channel for mtDNA cytoplasmic escape occurred through the opening of mPTP. By inhibiting the opening of mPTP, the release of mtDNA into the cytoplasm can be reduced, thereby diminishing the activation of the cGAS-STING pathway and ameliorating the inflammatory state within the internal environment.

In the pathological mechanisms of Kawasaki disease, mitochondrial damage and the activation of the cGAS-STING signaling pathway may play pivotal roles. Notably, the release of ND1 and COX1, components of mtDNA, into peripheral blood may reflect the extent of mitochondrial damage. Additionally, fluctuations in the expression levels of 2’3’-cGAMP, a direct product of the cGAS-STING pathway, may indicate the activation state of this signaling pathway. These biomarker changes could not only aid in the early diagnosis of Kawasaki disease but also deepen our understanding of its pathophysiological processes [32–35]. Our research demonstrates that the expression of mitochondrial DNA-specific genes ND1 and COX1, as well as the pathway product 2’3’-cGAMP, is significantly elevated in Kawasaki disease (Fig. 1), providing substantial theoretical support for studies on mitochondrial damage in Kawasaki disease. This aligns with the proposed critical mechanisms of the prospective mitochondrial double-hit model in Kawasaki disease: the susceptibility of endothelial cell mitochondria to mtDNA “leakage” due to impaired mitochondrial structure or functional mutations. These mutated mitochondria are prone to influence by infectious agents and are further impaired during the infection process, resulting in a dysfunctional state. This includes the overactivation of the NLRP3 inflammasome and the release of pro-inflammatory cytokines, thereby affecting the expression of circulating immune cells, including macrophages/monocytes and neutrophils, and further amplifying the inflammatory response [36]. Hence, our subsequent research focused on exploring new mechanisms in which mtDNA plays a pro-inflammatory role in KD.

The release of mtDNA is considered one of the triggers for intracellular inflammatory responses, with free mtDNA being cleared by mechanisms such as autophagy. When not cleared, mtDNA can activate the immune response via the cGAS-STING signaling pathway. Studies show that autophagy is impaired in Kawasaki disease [37], leading to an obstruction in the clearing mechanism and an increase in the release of contents such as mtDNA. Our observations in the KD in vitro model using confocal microscopy vividly capture the cytoplasmic escape of mtDNA (Fig. 4G), confirming the activation and suppression of the downstream cGAS-STING pathway, which can regulate the production and alleviation of inflammatory responses (Figs. 3D and 5D). The cGAS-STING signaling pathway is a crucial intracellular immune surveillance mechanism and plays a key role in the development of various diseases. When mitochondrial DNA escapes to the cytoplasm following damage, cGAS recognizes these non-nuclear DNAs, thereby activating STING. The activation of STING leads to the phosphorylation of transcription factors IRF3 and NF-κB, subsequently inducing the expression of interferons and other inflammatory factors [38]. This process has been reported in the inflammatory responses and hypertrophy of cardiac myocytes, myocardial infarction, and atherosclerosis [39–41], and was also observed in our study. Additionally, the activation of the cGAS-STING signal pathway is also associated with cell pyroptosis, a form of programmed cell death accompanied by inflammatory response [42].

The release of mtDNA involves various cellular processes, most crucially the opening of release channels. mPTP is a common outcome under various pathological conditions, such as oxidative stress [43]. Its opening provides a pathway for mtDNA to escape to the cytoplasm. In our study, an increase in mtROS production in KD induced the opening of mPTP (Figs. 4A and 7C). However, ROS produced by one mitochondrion may interact with adjacent mitochondria, briefly reopening mPTP and causing changes in MMP (Fig. 4B). This cascading effect has catastrophic consequences for the organelle, causing rapid mitochondrial swelling and the accumulation of mitochondrial contents like mtDNA and CytC in the cytoplasm, inducing cell death. The length of mtDNA is approximately 16.3 kb, and it can be compacted by TFAM into non-mPTP or VDAC-penetrable matrix-attached nucleoids [36]. In our KD in vitro model, we also discovered a reduction in TFAM expression in KD, leading to unstable mtDNA structure and ease of release (Fig. 7A). Besides the mPTP located between the mitochondrial inner and outer membranes, mtDNA must also pass through two additional barriers to reach the cytoplasmic sol—the mitochondrial inner and outer membranes. Key factors include outer membrane protein VDAC/TOM20 and key regulators mediating OMM permeability, BAX, and BAK [44]. In our constructed KD in vitro model, we found reduced expression of mitochondrial membrane protein VDAC and TOM20 (Fig. 7E), with the specific mechanism potentially related to oligomerization under disease conditions. Simultaneously, we also discovered increased expression of the critical apoptotic factor BAX mediating OMM permeability, and cytoplasmic accumulation of CytC (Fig. 7G), revealing that BAX/BAK accumulation in the mitochondrial outer membrane forms a channel that mediates permeability during the KD disease process. However, a limitation of our experiment is the lack of direct observation of this process under confocal microscopy. Subsequently, after incorporating the mPTP inhibitor CsA into our experiments, these channels closed, mitochondrial content release decreased, pathways were inhibited, and inflammation was alleviated. These results all suggest that targeting mitochondrial stress and mtDNA release channels might mitigate inflammation by regulating the cGAS-STING signaling pathway. Nevertheless, a more comprehensive understanding of the complex relationship between mtDNA and this signaling pathway requires further exploration. For instance, when exposed to certain intensities of damage or stimuli, cells may opt for necroptotic death rather than classic apoptosis. At this time, the phosphorylation of mixed lineage kinase domain-like protein (MLKL) occurs, adopting an active conformation that forms pores or directly damages cell membrane integrity, ultimately leading to cell death, releasing mtDNA to activate cGAS-STING, which has been confirmed in the disease process of aortic dissection [41], and whether MLKL phosphorylation mediated mtDNA release also exists in Kawasaki disease requires further study. Moreover, interestingly, recent studies have found that oxidative mtDNA produced by mitochondrial internal stress can be cleaved, and these fragments leave the mitochondria through mPTP and VDAC-dependent channels to initiate cytoplasmic NLRP3 inflammasome activation. Oxidized mtDNA fragments also activate cGAS-STING signal transduction and produce pro-inflammatory extracellular DNA [28]. This is generally consistent with our experimental results, but our experiments focused on the consequences caused by mtDNA, without fully delving into the process. Including the formation of neutrophil extracellular traps by mtDNA could also have significant implications for the development of KD disease.

In conclusion, this study reveals that the cytoplasmic release of mtDNA and the activation of cGAS-STING play crucial roles in the inflammatory response in Kawasaki disease. We believe that targeting the release of mtDNA to cGAS-STING could potentially offer new therapeutic targets for the treatment of KD.

## Conclusions

Our present study concluded that mitochondrial DNA activates the cGAS-STING signalling pathway via mPTP release, which is involved in the inflammatory response in Kawasaki disease. This response can be improved by inhibiting the release of mitochondrial DNA, which may be a new target for our future treatment of Kawasaki disease.

### Supplementary Information

Supplementary Material 1.

Supplementary Material 2.

Supplementary Material 3.

## Data Availability

No datasets were generated or analysed during the current study.

## Abbreviations

mtDNA: Mitochondrial DNA
cGAS: Cyclic GMP-AMP synthase
STING: Stimulator of interferon genes
TBK1: TANK-binding kinase 1
NF-κB: Nuclear factor kappa-B
IRF3: Interferon regulatory factor 3
IL-6: Interleukin-6
IFNα: Interferon alpha
ISGs: Interferon-stimulated genes;
nDNA: Nuclear DNA
mtROS: Mitochondrial reactive oxygen species
mtDAMPs: Mitochondrial danger-associated molecular patterns
TFAM: Mitochondrial transcription factor A
mPTP: Mitochondrial permeability transition pore
CsA: Cyclosporine
IVIG: I.V. immunoglobulin

## References

[1] Gorelik M, Chung SA, Ardalan K, Binstadt BA, Friedman K, Hayward K, Imundo LF, Lapidus SK, Kim S, Son MB, Sule S, Tremoulet AH, Van Mater H, Yildirim-Toruner C, Langford CA, Maz M, Abril A, Guyatt G, Archer AM, Conn DL, Full KA, Grayson PC, Ibarra MF, Merkel PA, Rhee RL, Seo P, Stone JH, Sundel RP, Vitobaldi OI, Warner A, Byram K, Dua AB, Husainat N, James KE, Kalot M, Lin YC, Springer JM, Turgunbaev M, Villa-Forte A, Turner AS, Mustafa RA (2022). 2021 American College of Rheumatology/Vasculitis Foundation Guideline for the Management of Kawasaki Disease. Arthritis Care Res (Hoboken).

[2] Koch RE, Josefson CC, Hill GE (2017). Mitochondrial function, ornamentation, and immunocompetence. Biol Rev Camb Philos Soc.

[3] Yan C, Duanmu X, Zeng L, Liu B, Song Z (2019). Mitochondrial DNA: Distribution, Mutations, and Elimination. Cells.

[4] Boguszewska K, Szewczuk M, Kaźmierczak-Barańska J, Karwowski BT (2020). The Similarities between Human Mitochondria and Bacteria in the Context of Structure, Genome, and Base Excision Repair System. Molecules.

[5] Nogami H, Murachi S (1975). Phalangeal acroosteolysis associated with Down syndrome. Birth Defects Orig Artic Ser.

[6] Chistiakov DA, Shkurat TP, Melnichenko AA, Grechko AV, Orekhov AN (2018). The role of mitochondrial dysfunction in cardiovascular disease: a brief review. Ann Med.

[7] Hara T, Yamamura K, Sakai Y (2021). The up-to-date pathophysiology of Kawasaki disease. Clin Transl Immunology.

[8] Guo MM, Huang YH, Wang FS, Chang LS, Chen KD, Kuo HC (2022). CD36 is Associated With the Development of Coronary Artery Lesions in Patients With Kawasaki Disease. Front Immunol.

[9] De Gaetano A, Solodka K, Zanini G, Selleri V, Mattioli AV, Nasi M, Pinti M (2021). Molecular Mechanisms of mtDNA-Mediated Inflammation Cells.

[10] Wan D, Jiang W, Hao J (2020). Research Advances in How the cGAS-STING Pathway Controls the Cellular Inflammatory Response. Front Immunol.

[11] Decout A, Katz JD, Venkatraman S, Ablasser A (2021). The cGAS-STING pathway as a therapeutic target in inflammatory diseases. Nat Rev Immunol.

[12] Kato Y, Park J, Takamatsu H, Konaka H, Aoki W, Aburaya S, Ueda M, Nishide M, Koyama S, Hayama Y, Kinehara Y, Hirano T, Shima Y, Narazaki M, Kumanogoh A (2018). Apoptosis-derived membrane vesicles drive the cGAS-STING pathway and enhance type I IFN production in systemic lupus erythematosus. Ann Rheum Dis.

[13] Wang J, Li R, Lin H, Qiu Q, Lao M, Zeng S, Wang C, Xu S, Zou Y, Shi M, Liang L, Xu H, Xiao Y (2019). Accumulation of cytosolic dsDNA contributes to fibroblast-like synoviocytes-mediated rheumatoid arthritis synovial inflammation. Int Immunopharmacol.

[14] Correction to: Diagnosis, Treatment, and Long-Term Management of Kawasaki Disease: A Scientific Statement for Health Professionals From the American Heart Association. Circulation. 2019;140(5):e181-e184. 10.1161/CIR.0000000000000703 31356128

[15] Lehman TJ, Walker SM, Mahnovski V, McCurdy D (1985). Coronary arteritis in mice following the systemic injection of group B Lactobacillus casei cell walls in aqueous suspension. Arthritis Rheum.

[16] Murata H (1979). Experimental candida-induced arteritis in mice. Relation to arteritis in the mucocutaneous lymph node syndrome. Microbiol Immunol.

[17] Guo Y, Gu R, Gan D, Hu F, Li G, Xu G (2020). Mitochondrial DNA drives noncanonical inflammation activation via cGAS-STING signaling pathway in retinal microvascular endothelial cells. Cell Commun Signal.

[18] Heilig R, Lee J, Tait SWG (2023). Mitochondrial DNA in cell death and inflammation. Biochem Soc Trans.

[19] Kim J, Kim HS, Chung JH (2023). Molecular mechanisms of mitochondrial DNA release and activation of the cGAS-STING pathway. Exp Mol Med.

[20] Abe M, Rastelli DD, Gomez AC, Cingolani E, Lee Y, Soni PR, Fishbein MC, Lehman TJA, Shimada K, Crother TR, Chen S, Noval Rivas M, Arditi M (2020). IL-1-dependent electrophysiological changes and cardiac neural remodeling in a mouse model of Kawasaki disease vasculitis. Clin Exp Immunol.

[21] Chen T, Xu T, Cheng M, Fang H, Shen X, Tang Z, Zhao J (2021). Human umbilical cord mesenchymal stem cells regulate CD54 and CD105 in vascular endothelial cells and suppress inflammation in Kawasaki disease. Exp Cell Res.

[22] Kozlov AV, Bahrami S, Calzia E, Dungel P, Gille L, Kuznetsov AV, Troppmair J (2011). Mitochondrial dysfunction and biogenesis: do ICU patients die from mitochondrial failure?. Ann Intensive Care.

[23] Suski JM, Lebiedzinska M, Bonora M, Pinton P, Duszynski J, Wieckowski MR (2012). Relation between mitochondrial membrane potential and ROS formation. Methods Mol Biol.

[24] Zhou L, Zhang YF, Yang FH, Mao HQ, Chen Z, Zhang L (2021). Mitochondrial DNA leakage induces odontoblast inflammation via the cGAS-STING pathway. Cell Commun Signal.

[25] Fang R, Jiang Q, Guan Y, Gao P, Zhang R, Zhao Z, Jiang Z (2021). Golgi apparatus-synthesized sulfated glycosaminoglycans mediate polymerization and activation of the cGAMP sensor STING. Immunity.

[26] Lama L, Adura C, Xie W, Tomita D, Kamei T, Kuryavyi V, Gogakos T, Steinberg JI, Miller M, Ramos-Espiritu L, Asano Y, Hashizume S, Aida J, Imaeda T, Okamoto R, Jennings AJ, Michino M, Kuroita T, Stamford A, Gao P, Meinke P, Glickman JF, Patel DJ, Tuschl T (2019). Development of human cGAS-specific small-molecule inhibitors for repression of dsDNA-triggered interferon expression. Nat Commun.

[27] Zhao M, Wang Y, Li L, Liu S, Wang C, Yuan Y, Yang G, Chen Y, Cheng J, Lu Y, Liu J (2021). Mitochondrial ROS promote mitochondrial dysfunction and inflammation in ischemic acute kidney injury by disrupting TFAM-mediated mtDNA maintenance. Theranostics.

[28] Xian H, Watari K, Sanchez-Lopez E, Offenberger J, Onyuru J, Sampath H, Ying W, Hoffman HM, Shadel GS, Karin M (2022). Oxidized DNA fragments exit mitochondria via mPTP- and VDAC-dependent channels to activate NLRP3 inflammasome and interferon signaling. Immunity.

[29] Mao Y, Chen Y, Cai W, Jiang W, Sun X, Zeng J, Wang H, Wang X, Dong W, Ma J, Jaspers RT, Huang S, Wu G (2023). CypD-mediated mitochondrial dysfunction contributes to titanium ion-induced MC3T3-E1 cell injury. Biochem Biophys Res Commun.

[30] Zheng H, Huang S, Wei G, Sun Y, Li C, Si X, Chen Y, Tang Z, Li X, Chen Y, Liao W, Liao Y, Bin J (2022). CircRNA Samd4 induces cardiac repair after myocardial infarction by blocking mitochondria-derived ROS output. Mol Ther.

[31] Hausenloy DJ, Boston-Griffiths EA, Yellon DM (2012). Cyclosporin A and cardioprotection: from investigative tool to therapeutic agent. Br J Pharmacol.

[32] Yan M, Li Y, Luo Q, Zeng W, Shao X, Li L, Wang Q, Wang D, Zhang Y, Diao H, Rong X, Bai Y, Guo J (2022). Mitochondrial damage and activation of the cytosolic DNA sensor cGAS-STING pathway lead to cardiac pyroptosis and hypertrophy in diabetic cardiomyopathy mice. Cell Death Discov.

[33] Li L, Liu F, Feng C, Chen Z, Zhang N, Mao J (2024). Role of mitochondrial dysfunction in kidney disease: Insights from the cGAS-STING signaling pathway. Chin Med J (Engl).

[34] Tan X, Chen Q, Chen Z, Sun Z, Chen W, Wei R (2024). Mitochondrial DNA-Activated cGAS-STING Signaling in Environmental Dry Eye. Invest Ophthalmol Vis Sci.

[35] Chen H, Lu X, Xu B, Cheng G, Li Y, Xie D. Saikosaponin d protects pancreatic acinar cells against cerulein-induced pyroptosis through alleviating mitochondrial damage and inhibiting cGAS-STING pathway. J Appl Toxicol. 2024. 10.1002/jat.4594 38462915

[36] Beckley MA, Shrestha S, Singh KK, Portman MA (2022). The role of mitochondria in the pathogenesis of Kawasaki disease. Front Immunol.

[37] Marek-Iannucci S, Ozdemir AB, Moreira D, Gomez AC, Lane M, Porritt RA, Lee Y, Shimada K, Abe M, Stotland A, Zemmour D, Parker S, Sanchez-Lopez E, Van Eyk J, Gottlieb RA, Fishbein MC, Karin M, Crother TR, Rivas MN, Arditi M (2021). Autophagy-mitophagy induction attenuates cardiovascular inflammation in a murine model of Kawasaki disease vasculitis. JCI Insight.

[38] Luo W, Zou X, Wang Y, Dong Z, Weng X, Pei Z, Song S, Zhao Y, Wei Z, Gao R, Zhang B, Liu L, Bai P, Liu J, Wang X, Gao T, Zhang Y, Sun X, Chen H, Hu K, Du S, Sun A, Ge J (2023). Critical Role of the cGAS-STING Pathway in Doxorubicin-Induced Cardiotoxicity. Circ Res.

[39] Zhang Y, Chen W, Wang Y (2020). STING is an essential regulator of heart inflammation and fibrosis in mice with pathological cardiac hypertrophy via endoplasmic reticulum (ER) stress. Biomed Pharmacother.

[40] Cao DJ, Schiattarella GG, Villalobos E, Jiang N, May HI, Li T, Chen ZJ, Gillette TG, Hill JA (2018). Cytosolic DNA Sensing Promotes Macrophage Transformation and Governs Myocardial Ischemic Injury. Circulation.

[41] Luo W, Wang Y, Zhang L, Ren P, Zhang C, Li Y, Azares AR, Zhang M, Guo J, Ghaghada KB, Starosolski ZA, Rajapakshe K, Coarfa C, Li Y, Chen R, Fujiwara K, Abe JI, Coselli JS, Milewicz DM, LeMaire SA, Shen YH (2020). Critical Role of Cytosolic DNA and Its Sensing Adaptor STING in Aortic Degeneration, Dissection, and Rupture. Circulation.

[42] Zhang W, Li G, Luo R, Lei J, Song Y, Wang B, Ma L, Liao Z, Ke W, Liu H, Hua W, Zhao K, Feng X, Wu X, Zhang Y, Wang K, Yang C (2022). Cytosolic escape of mitochondrial DNA triggers cGAS-STING-NLRP3 axis-dependent nucleus pulposus cell pyroptosis. Exp Mol Med.

[43] Halestrap AP (2009). What is the mitochondrial permeability transition pore?. J Mol Cell Cardiol.

[44] Cosentino K, Hertlein V, Jenner A, Dellmann T, Gojkovic M, Peña-Blanco A, Dadsena S, Wajngarten N, Danial JSH, Thevathasan JV, Mund M, Ries J, Garcia-Saez AJ (2022). The interplay between BAX and BAK tunes apoptotic pore growth to control mitochondrial-DNA-mediated inflammation. Mol Cell.

