# Supplementary figures and images for: Mitochondrial DNA release via the mitochondrial permeability transition pore activates the cGAS-STING pathway, exacerbating inflammation in acute Kawasaki disease
Source: Cell Commun Signal. 2024 Jun 13;22:328. doi: 10.1186/s12964-024-01677-9 (PMC11177463; doi:10.1186/s12964-024-01677-9)

Fig.7G fig8F


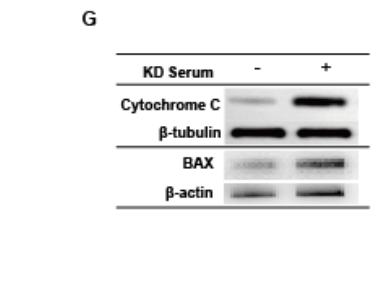

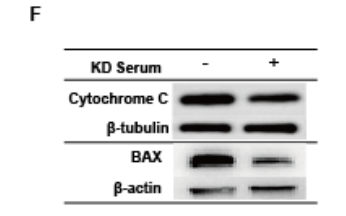


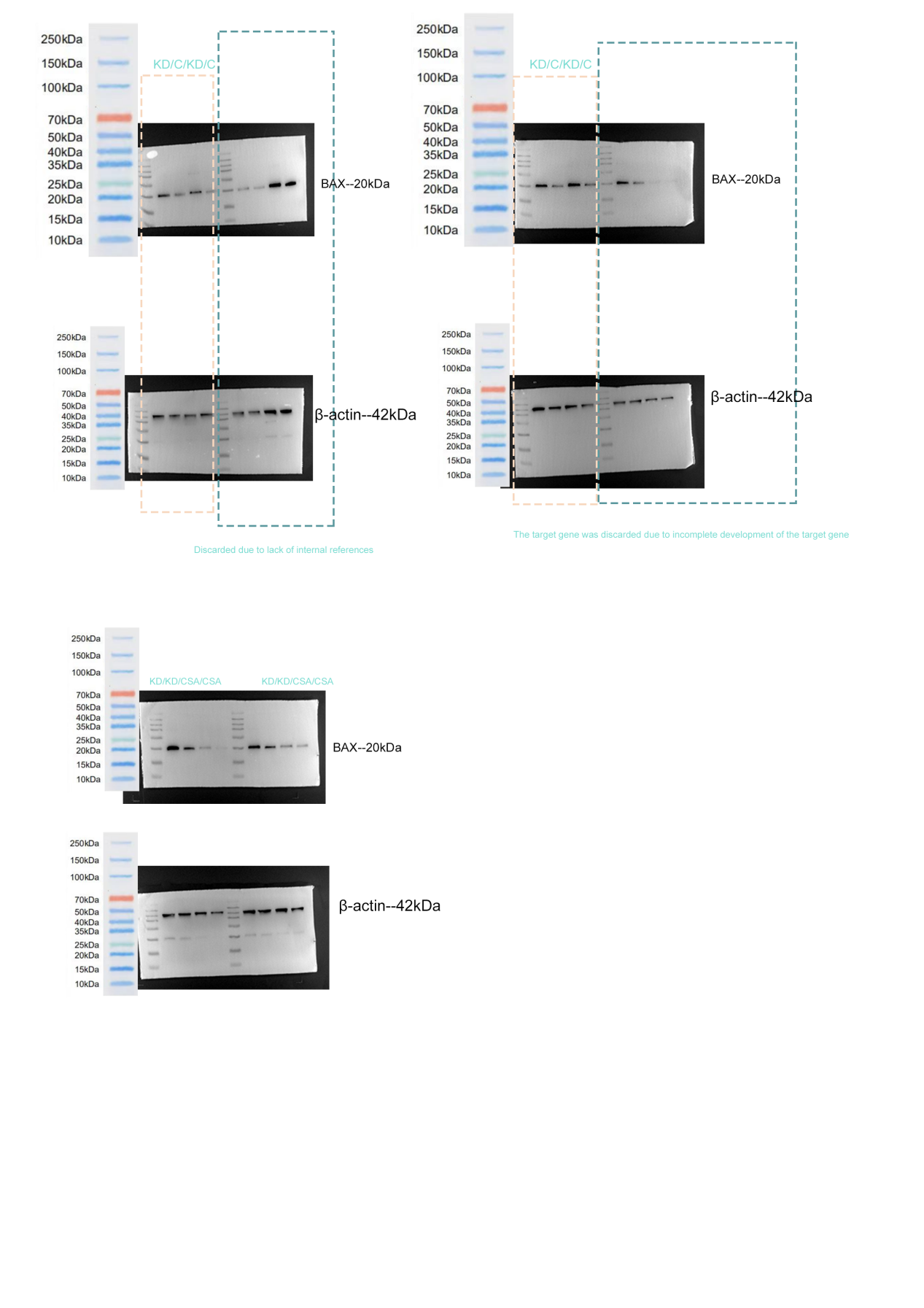


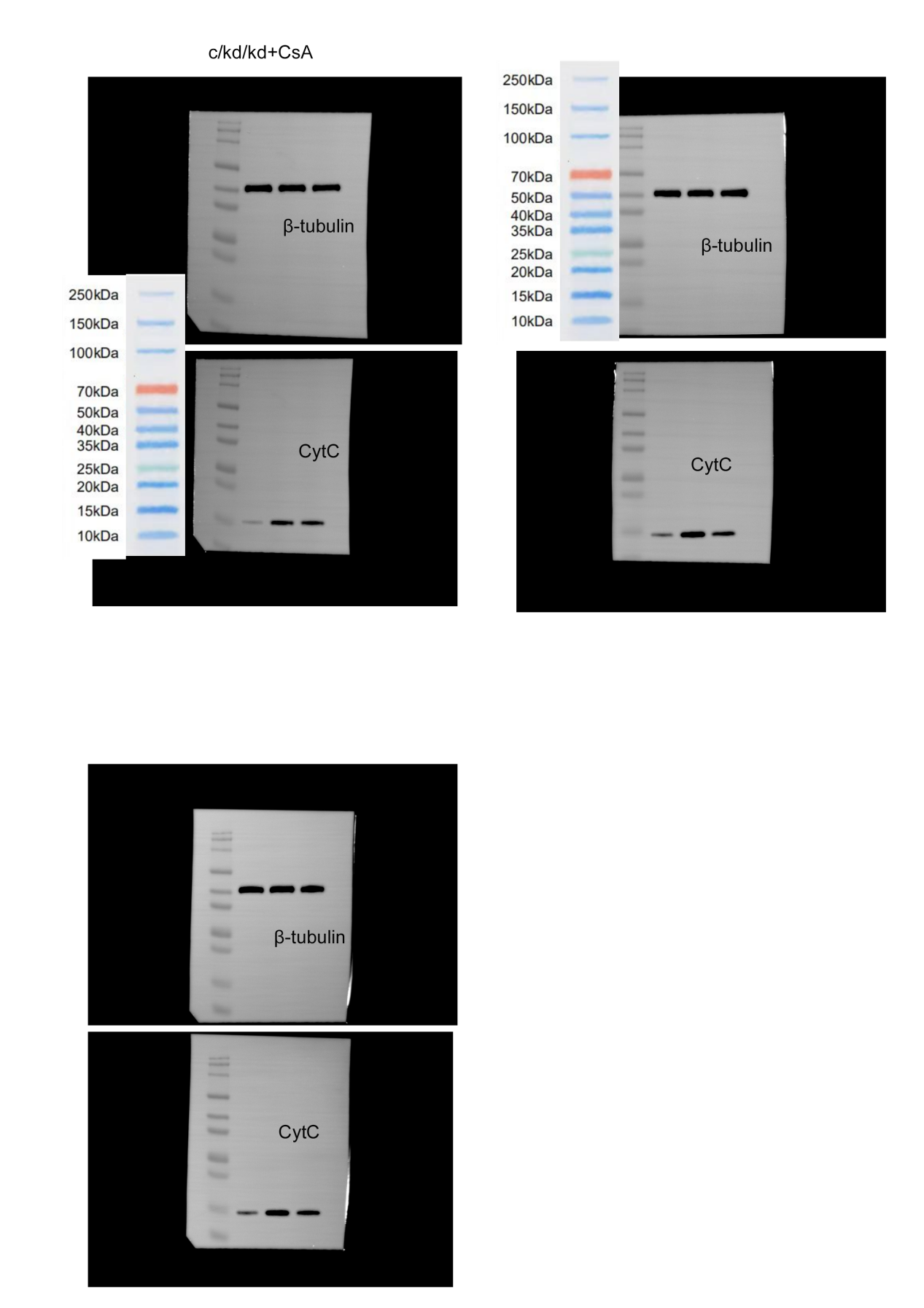


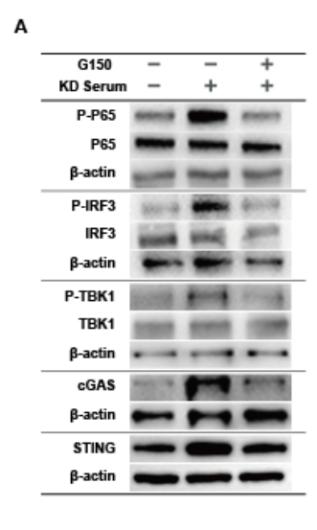
Fig6A


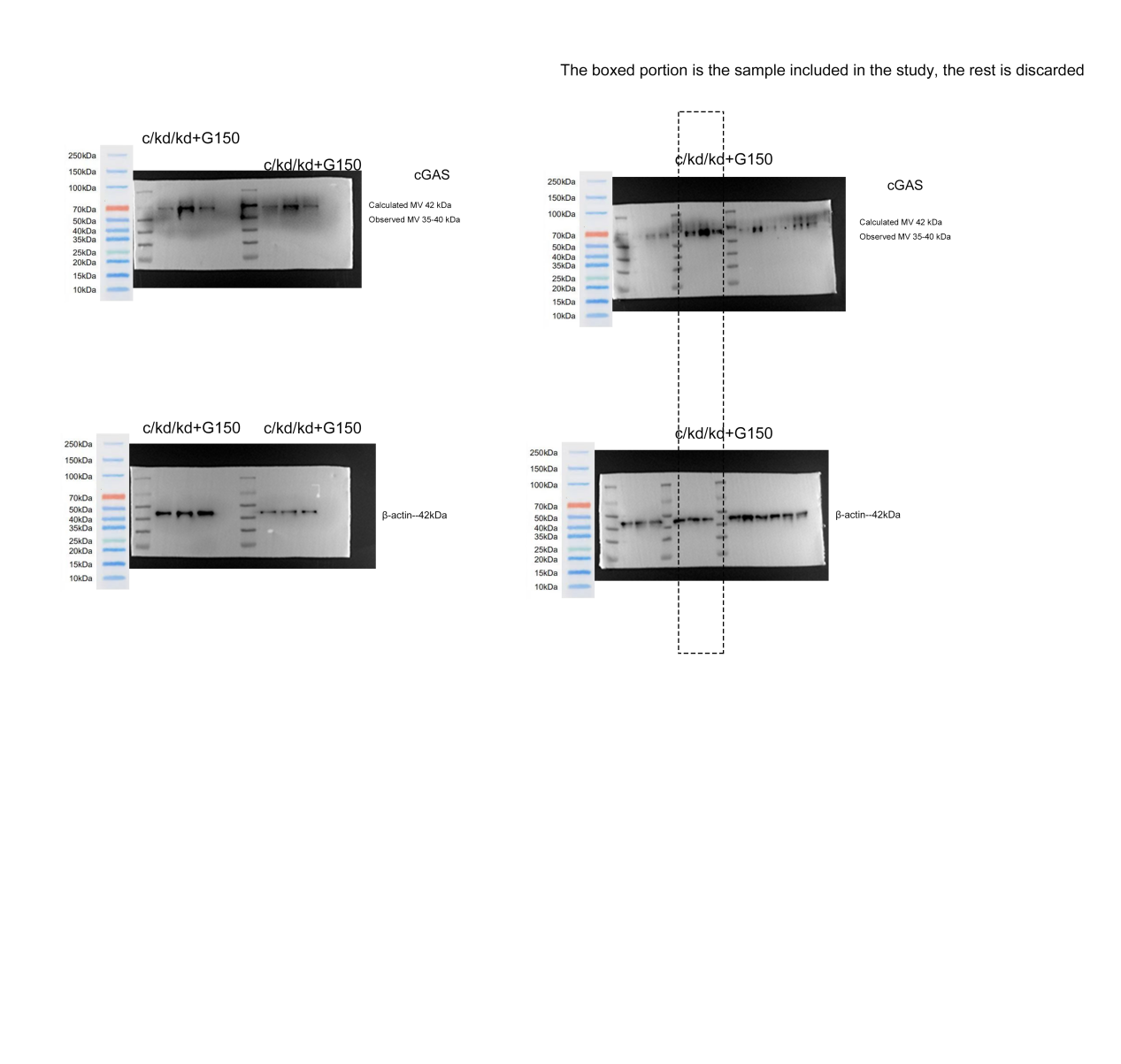


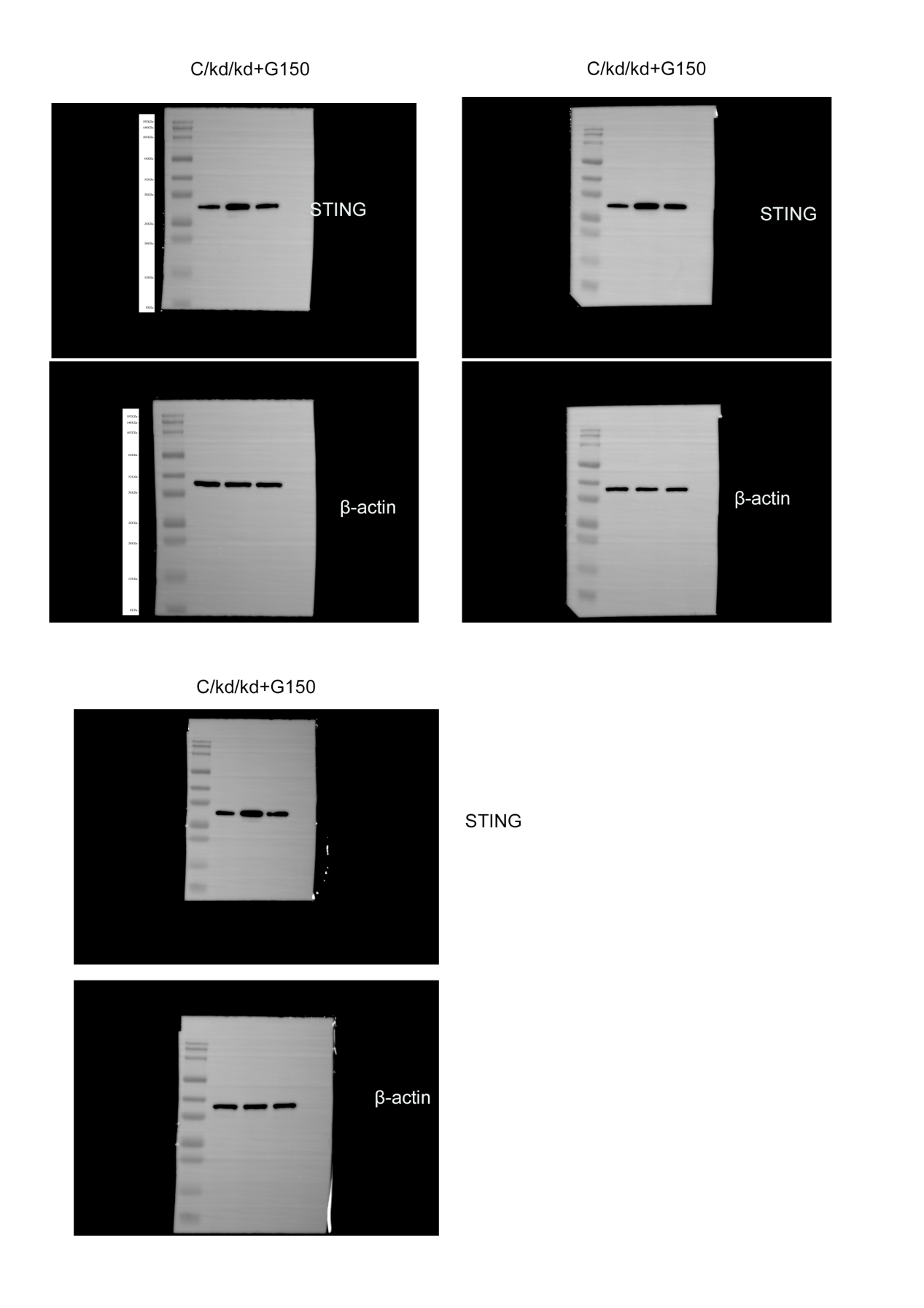


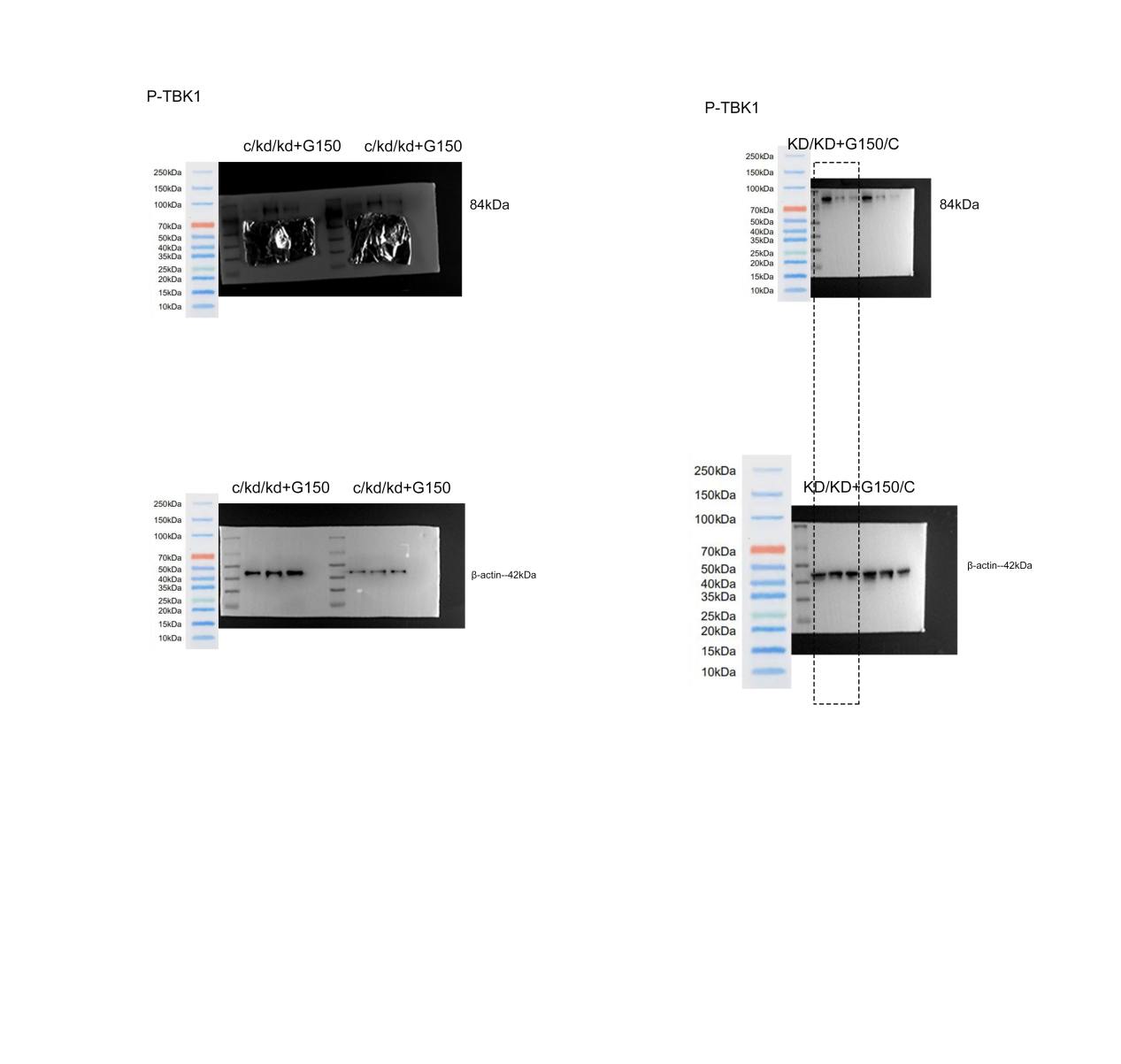


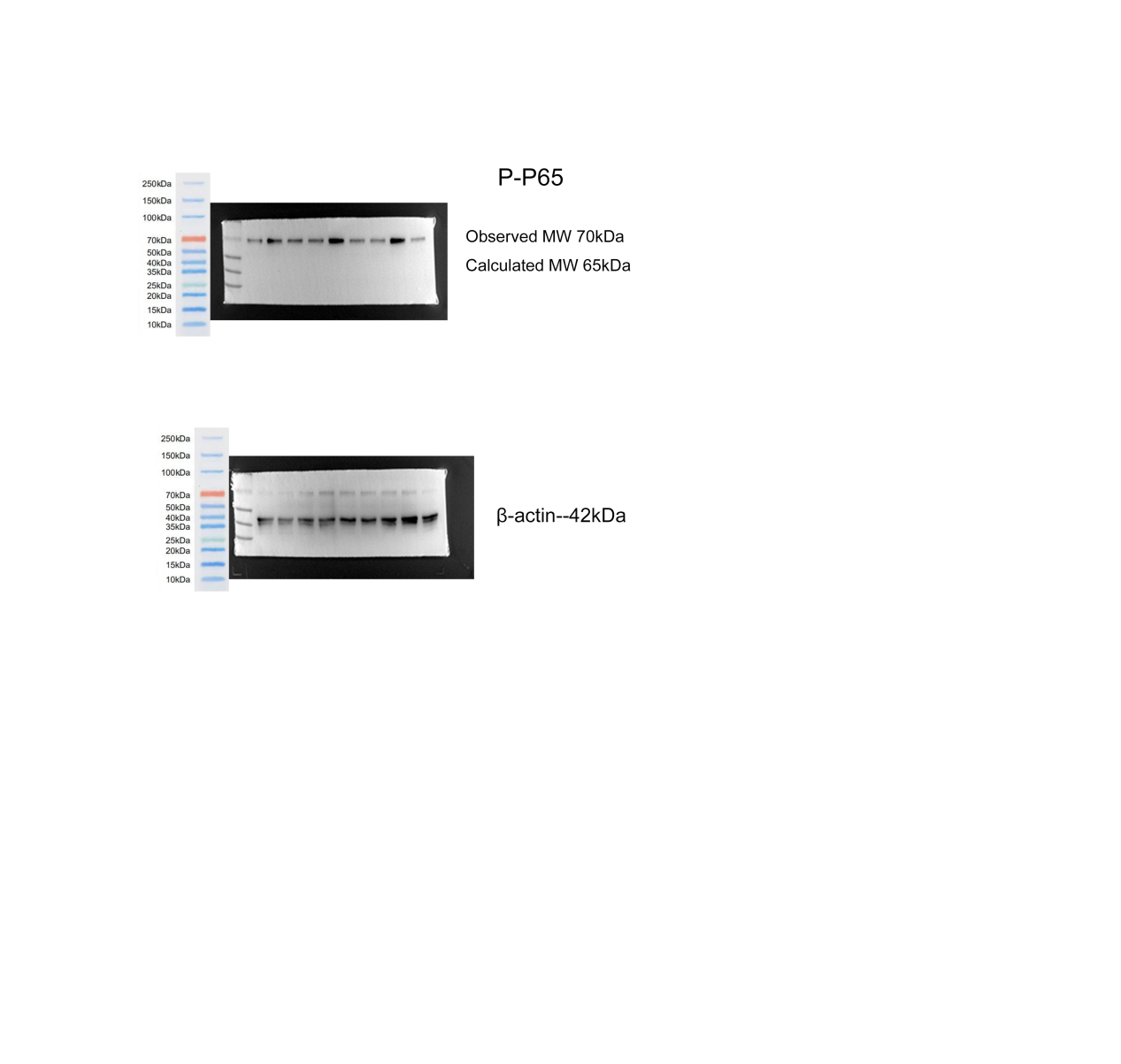


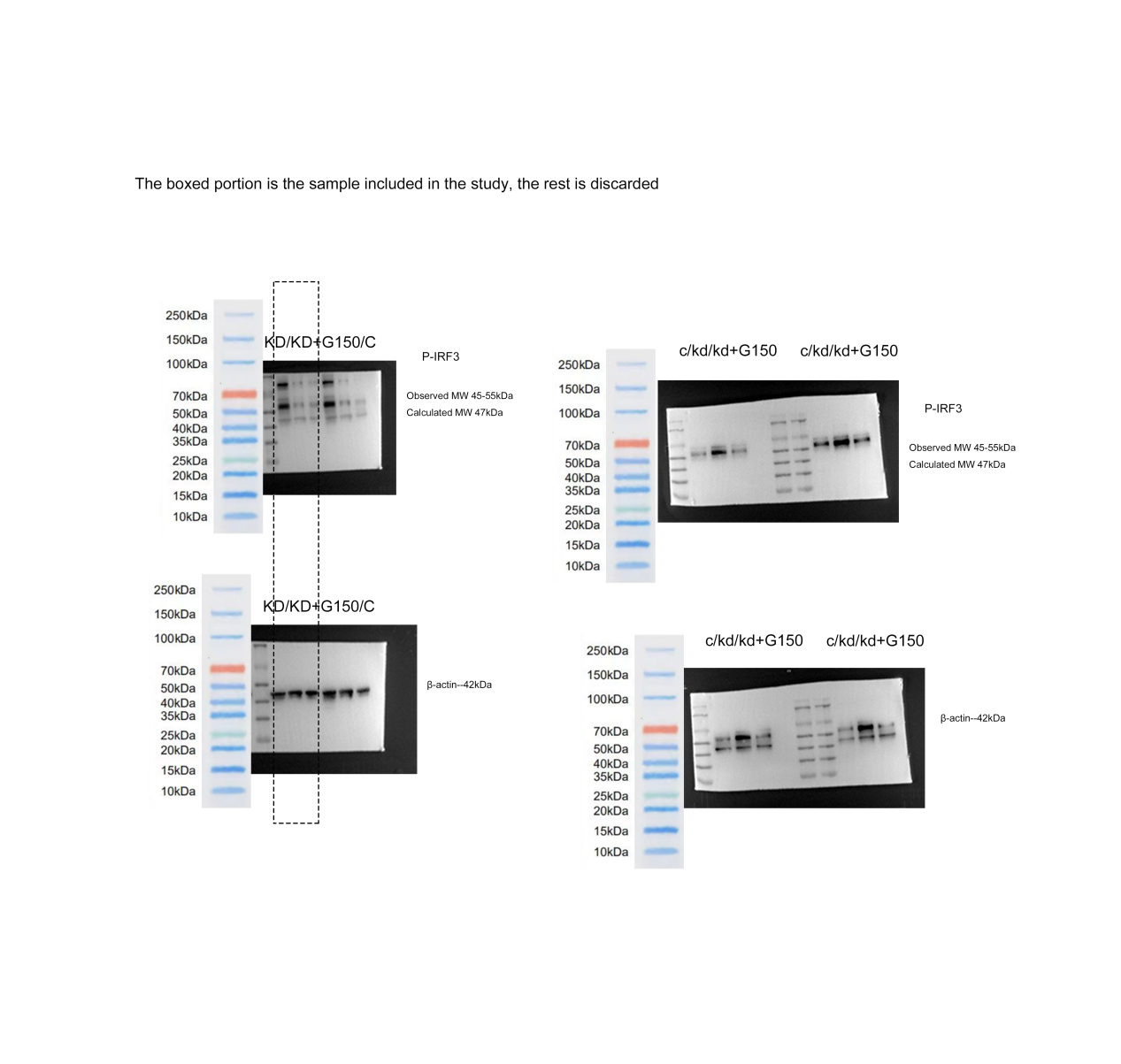


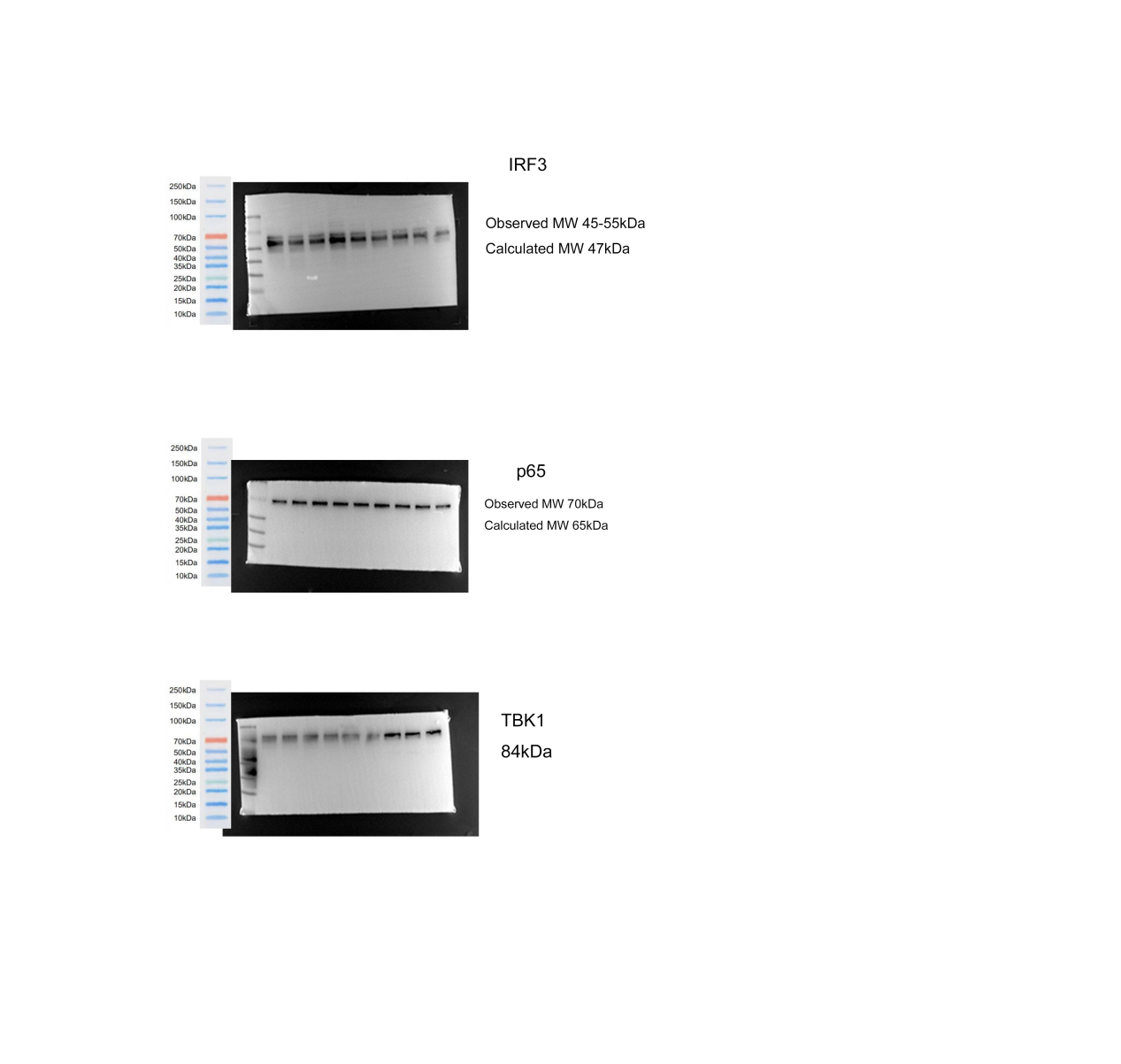

Supplement: Supplementary file 1 — Supplementary Material 1. [file 12964_2024_1677_MOESM1_ESM.docx]

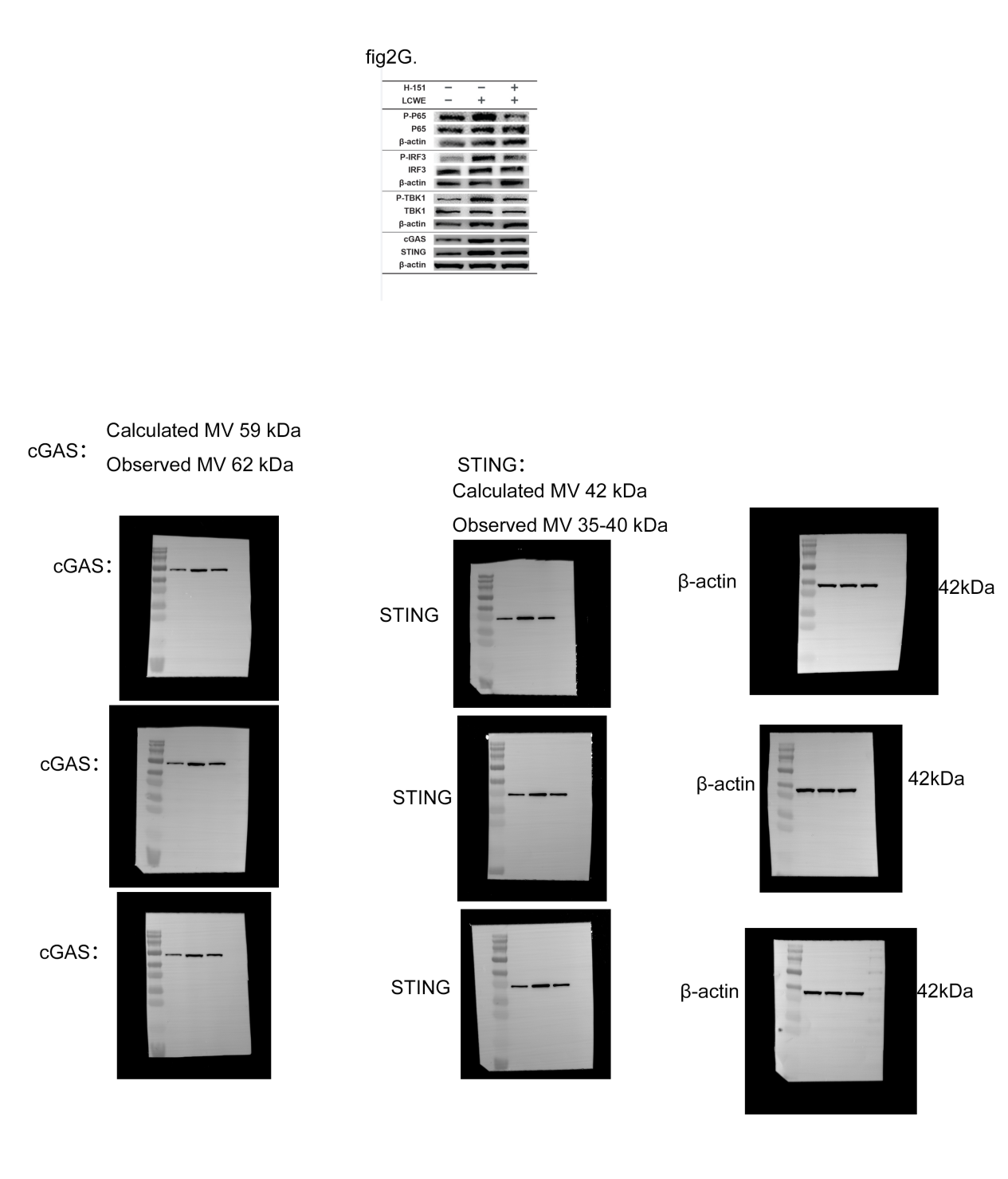


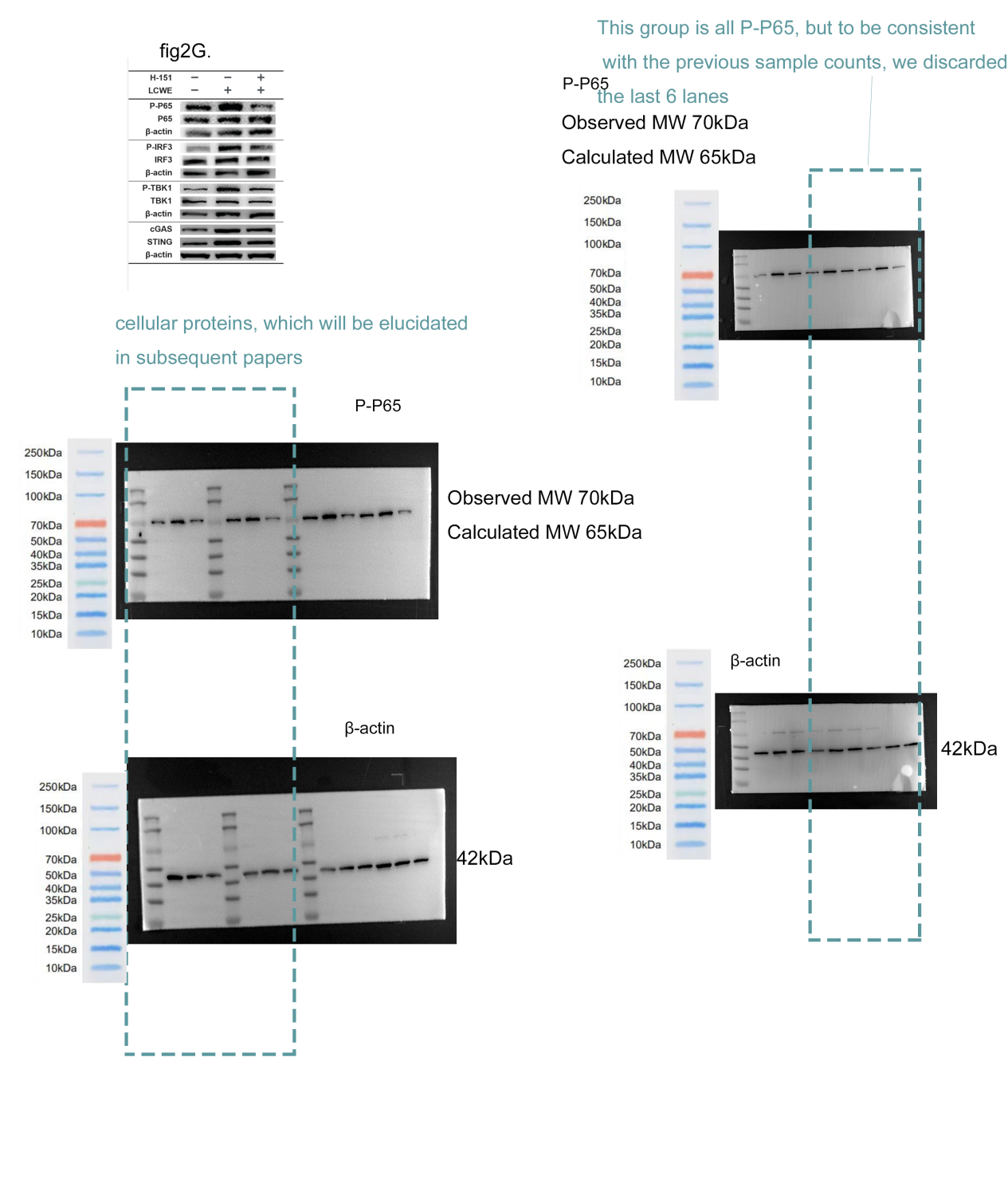


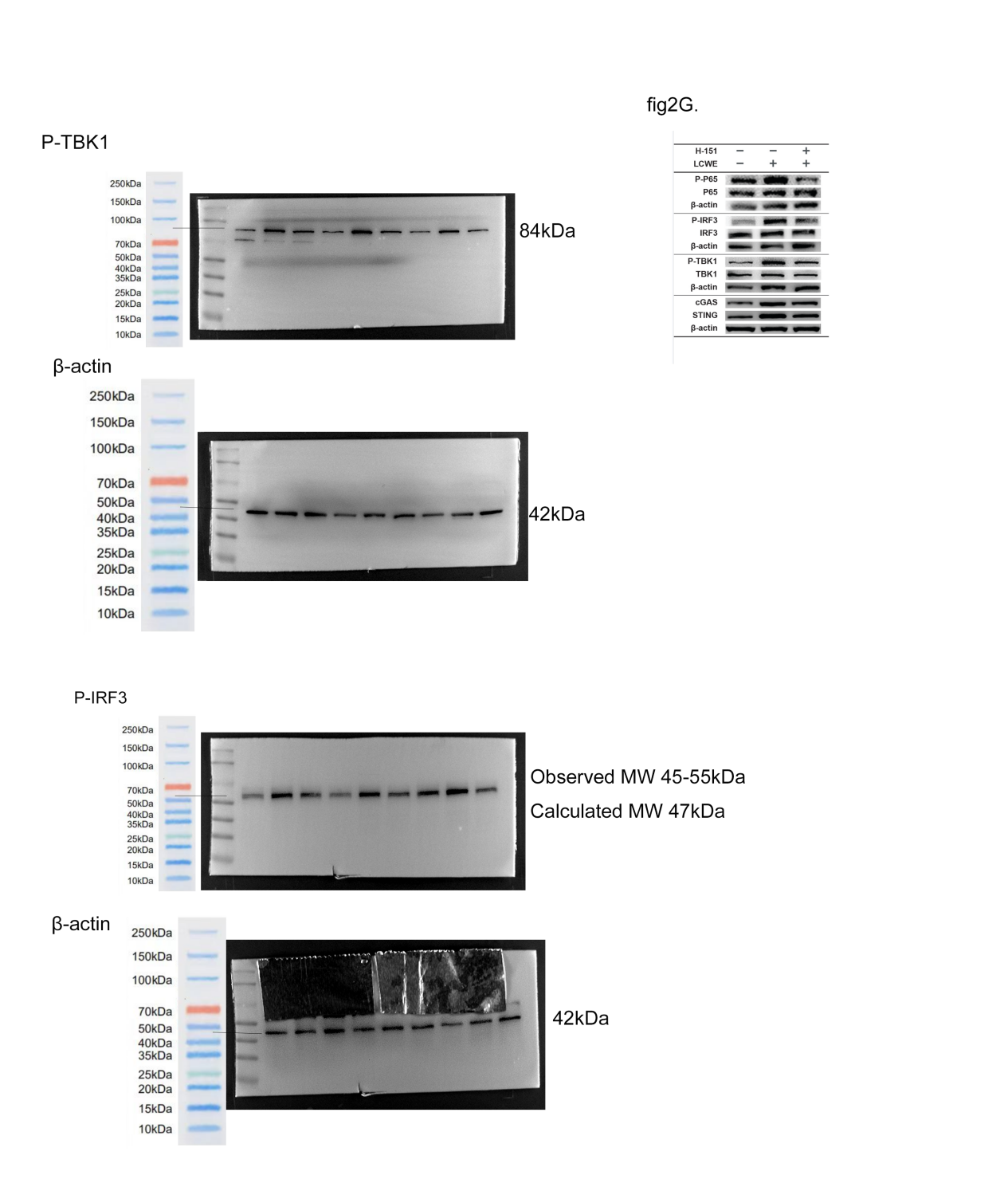


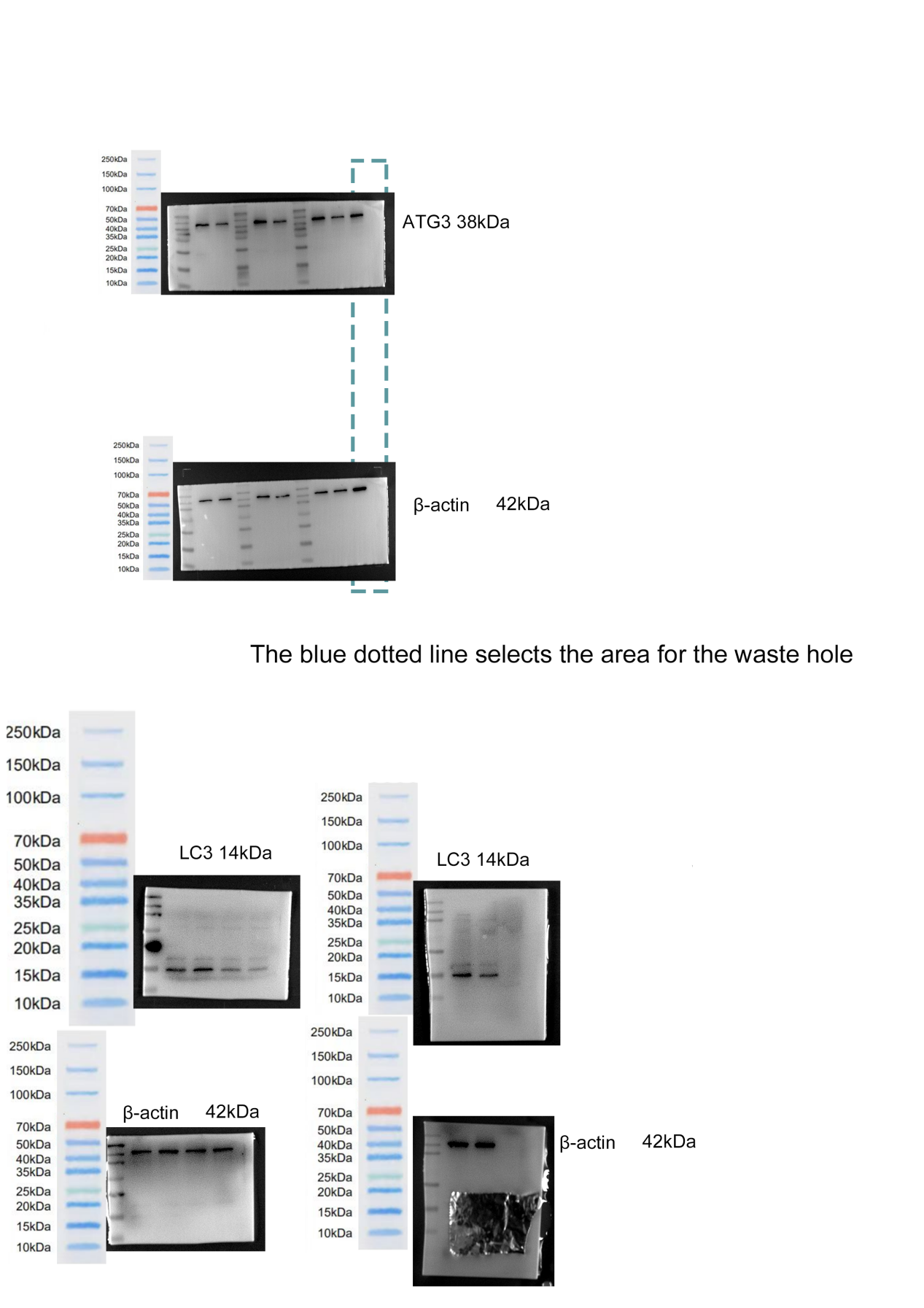


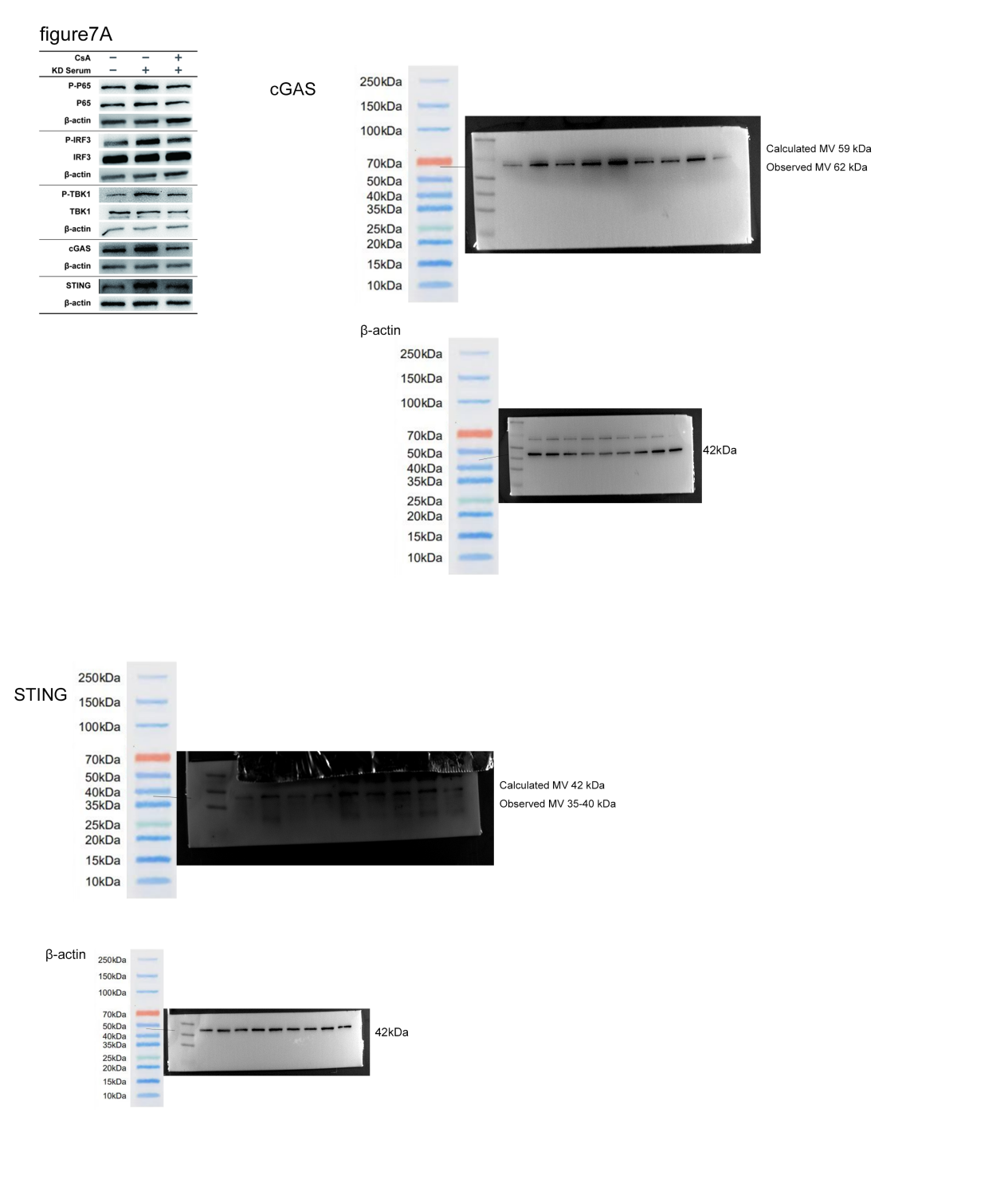


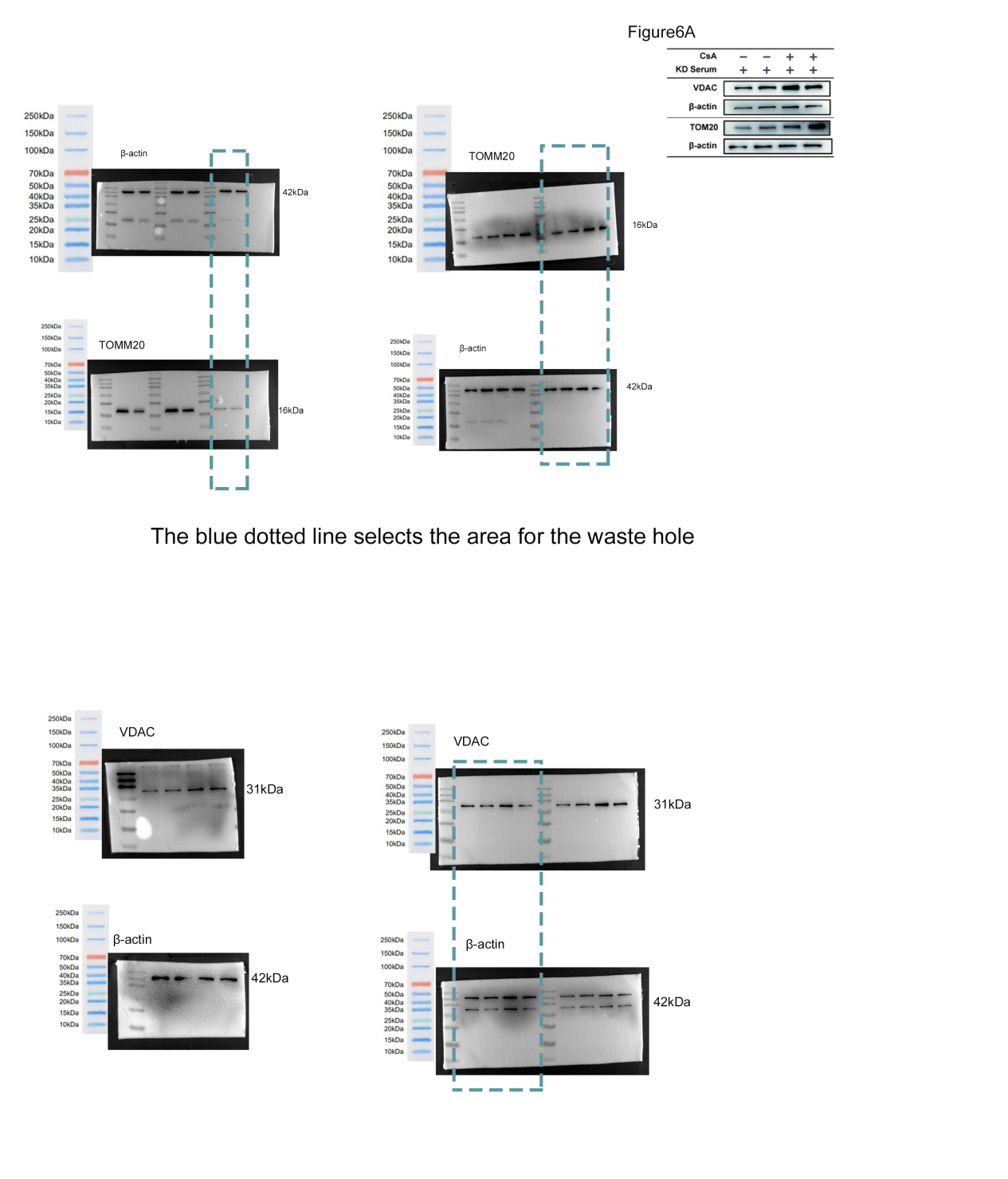


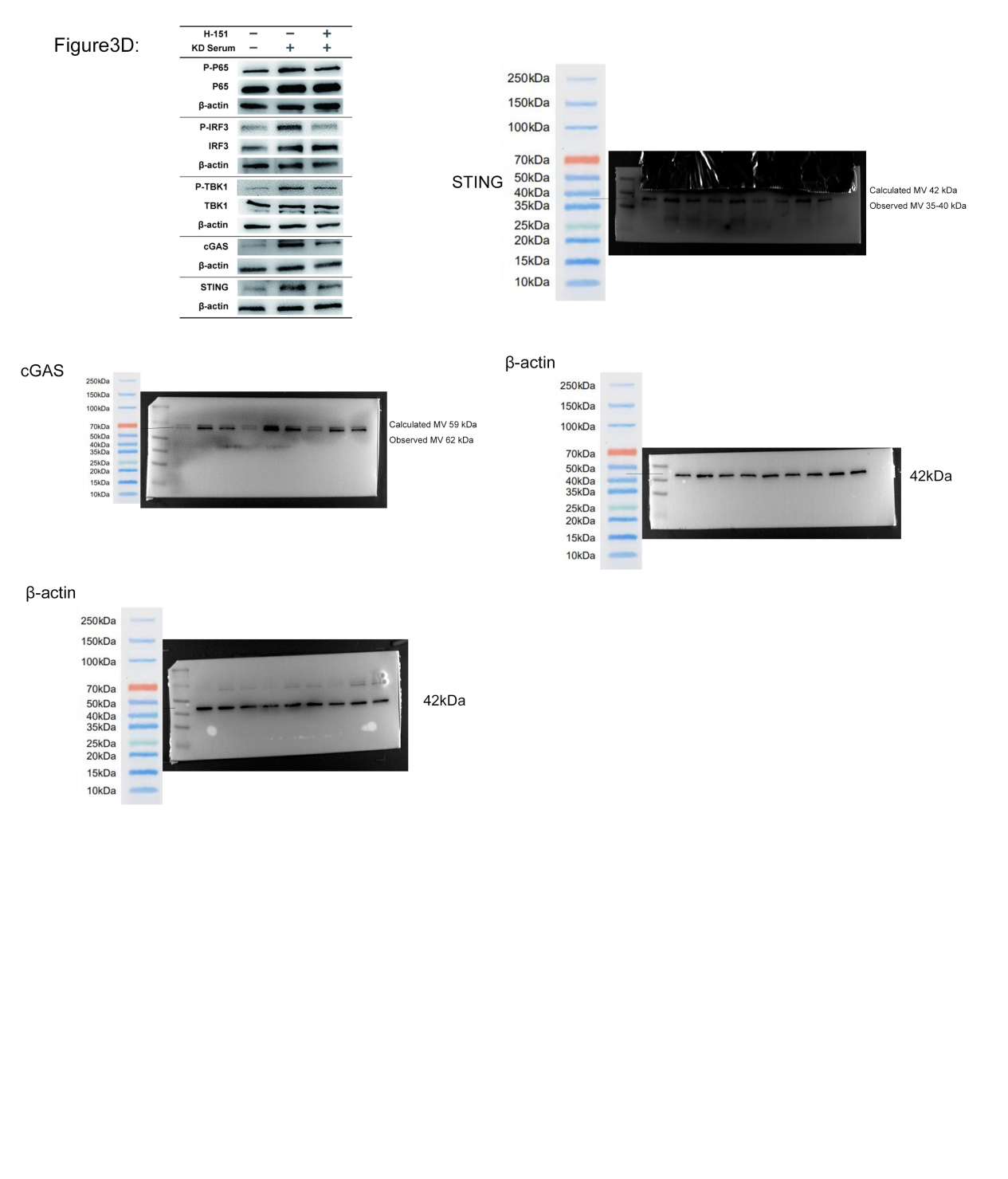


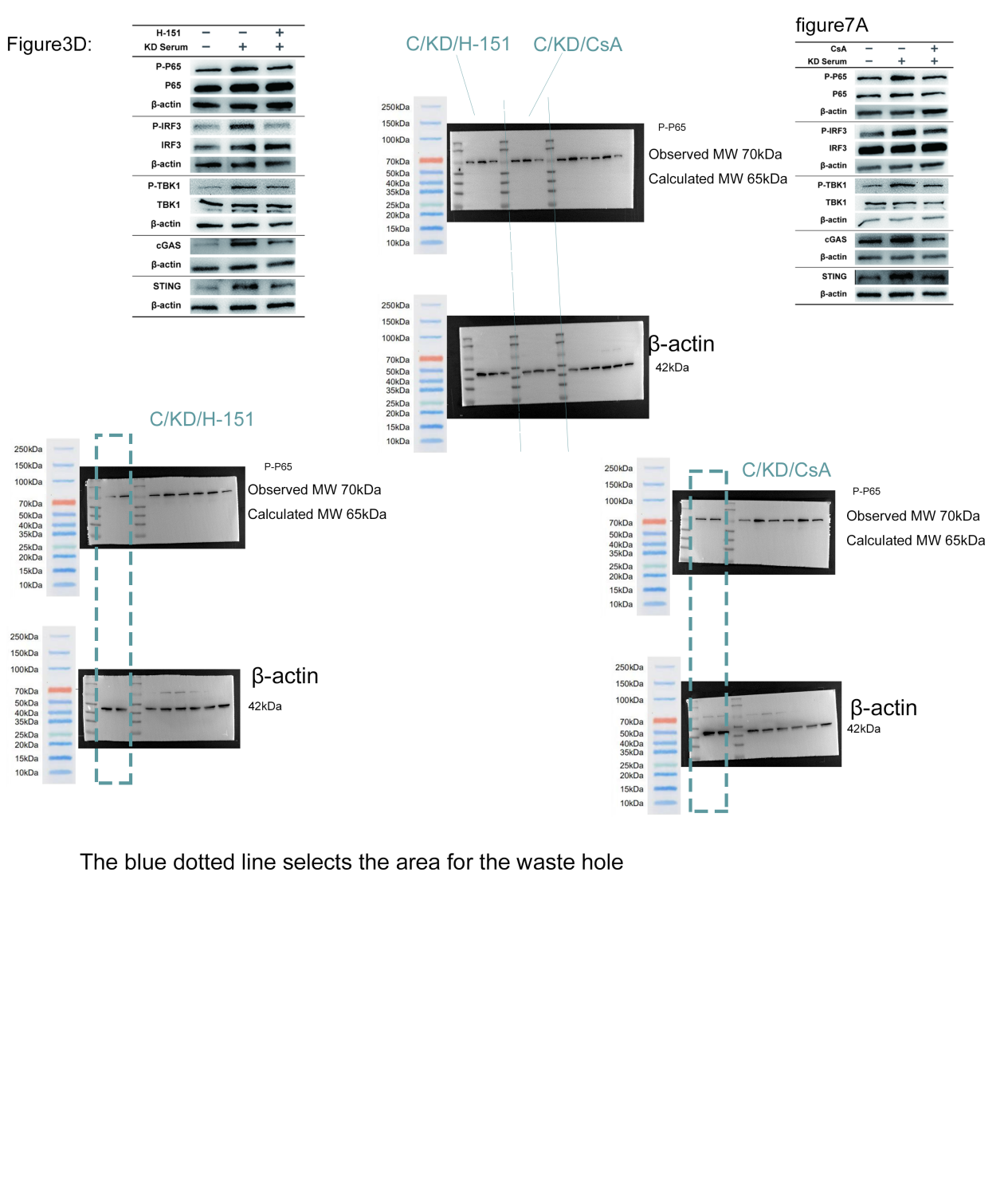


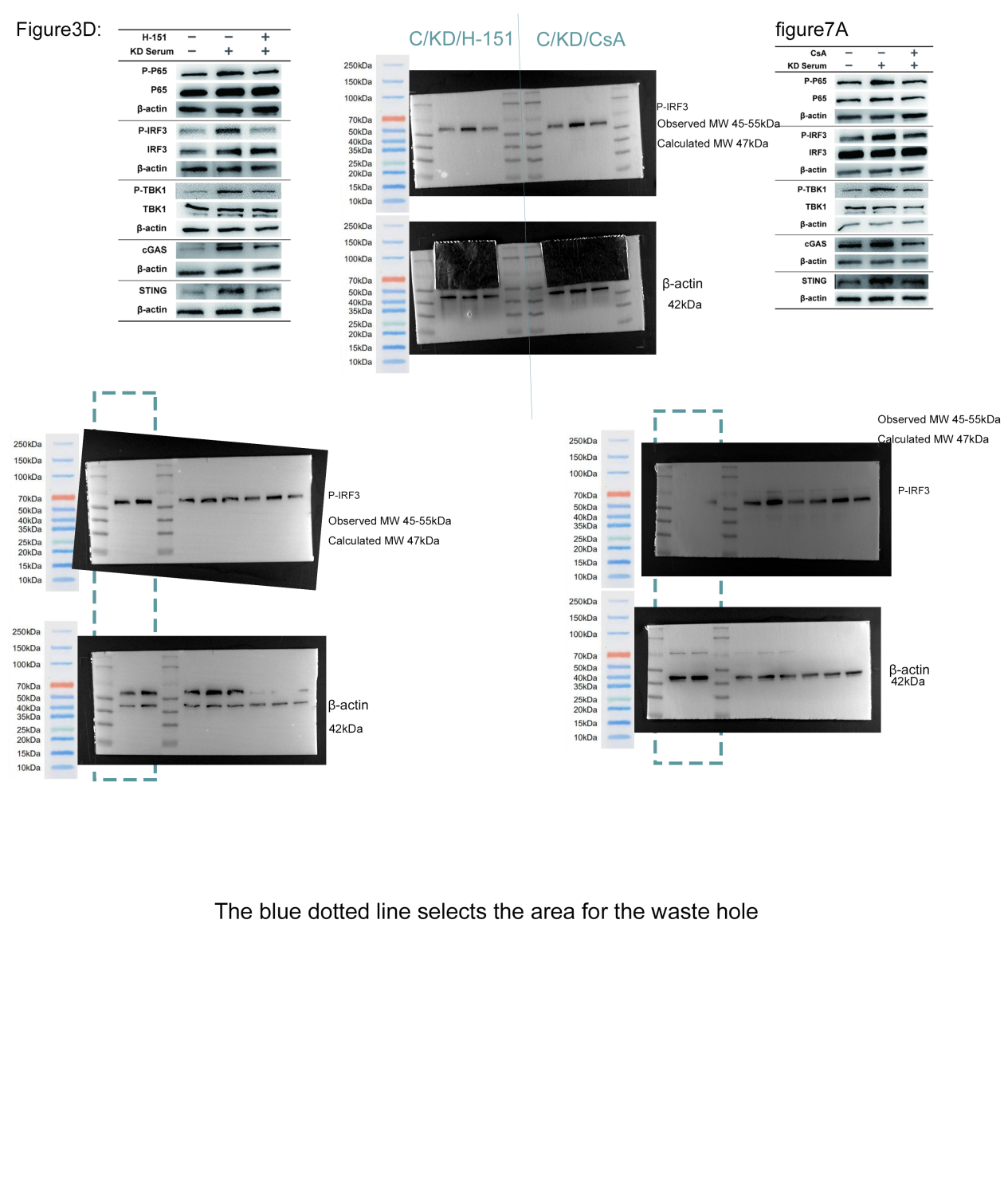


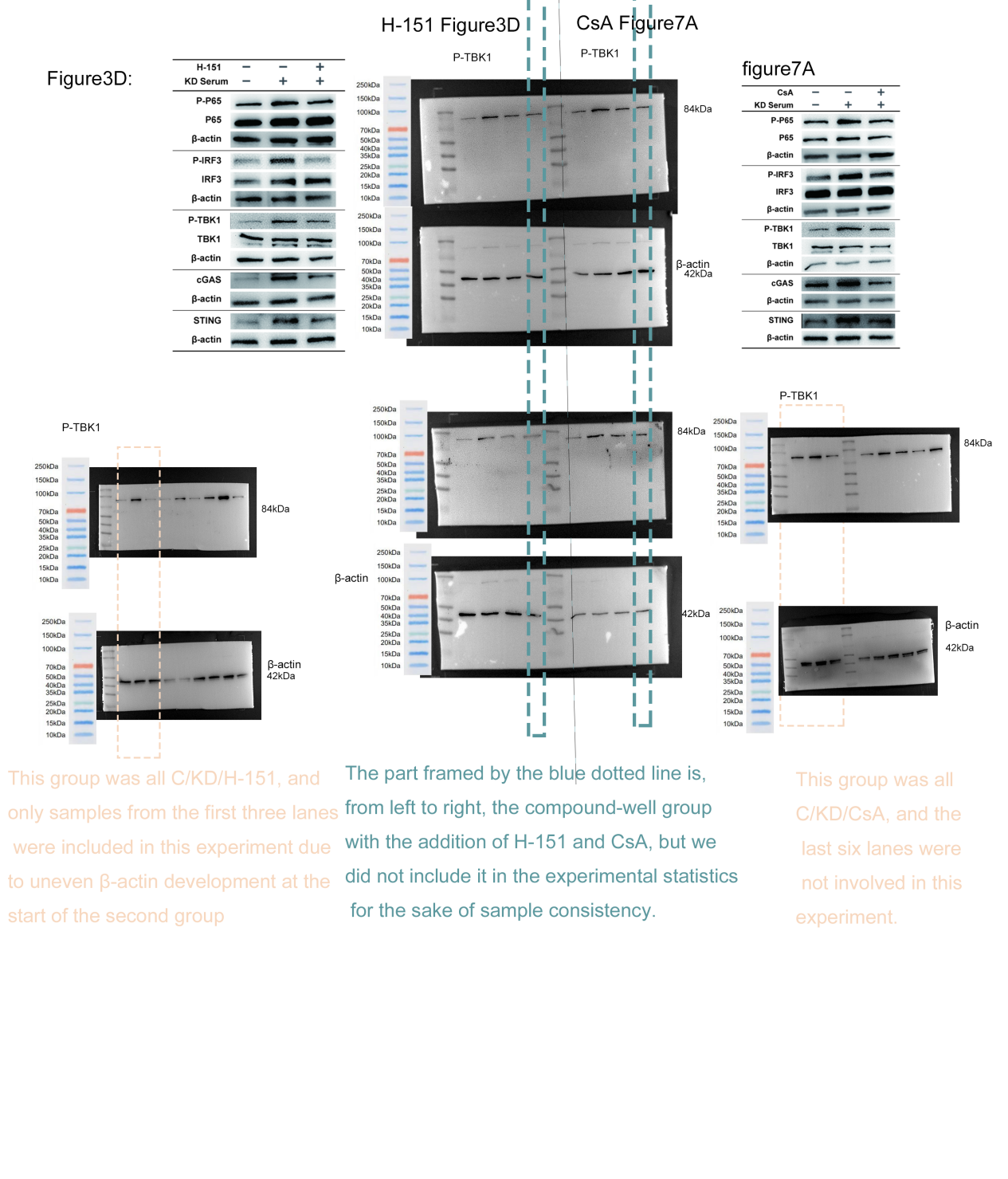


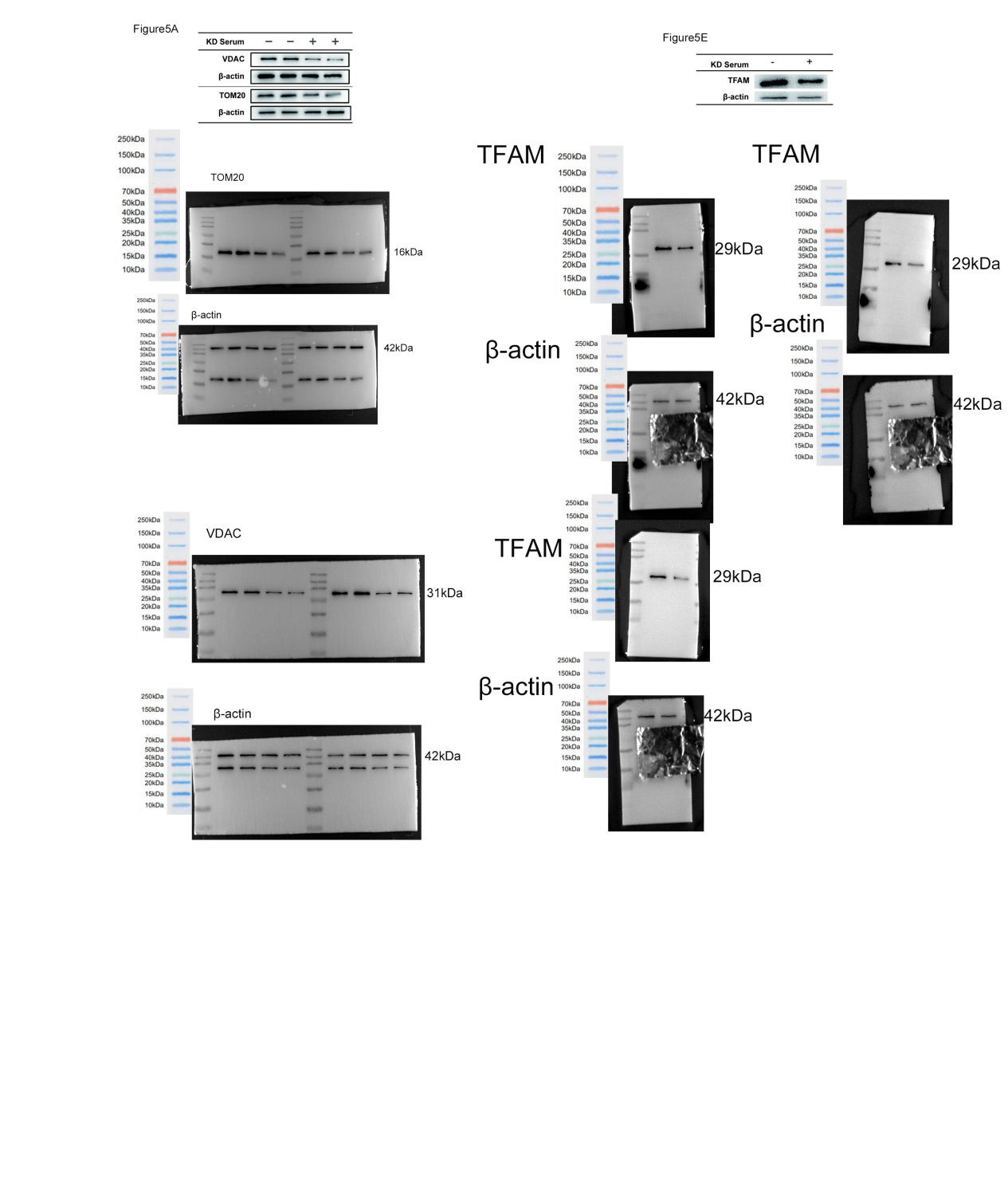


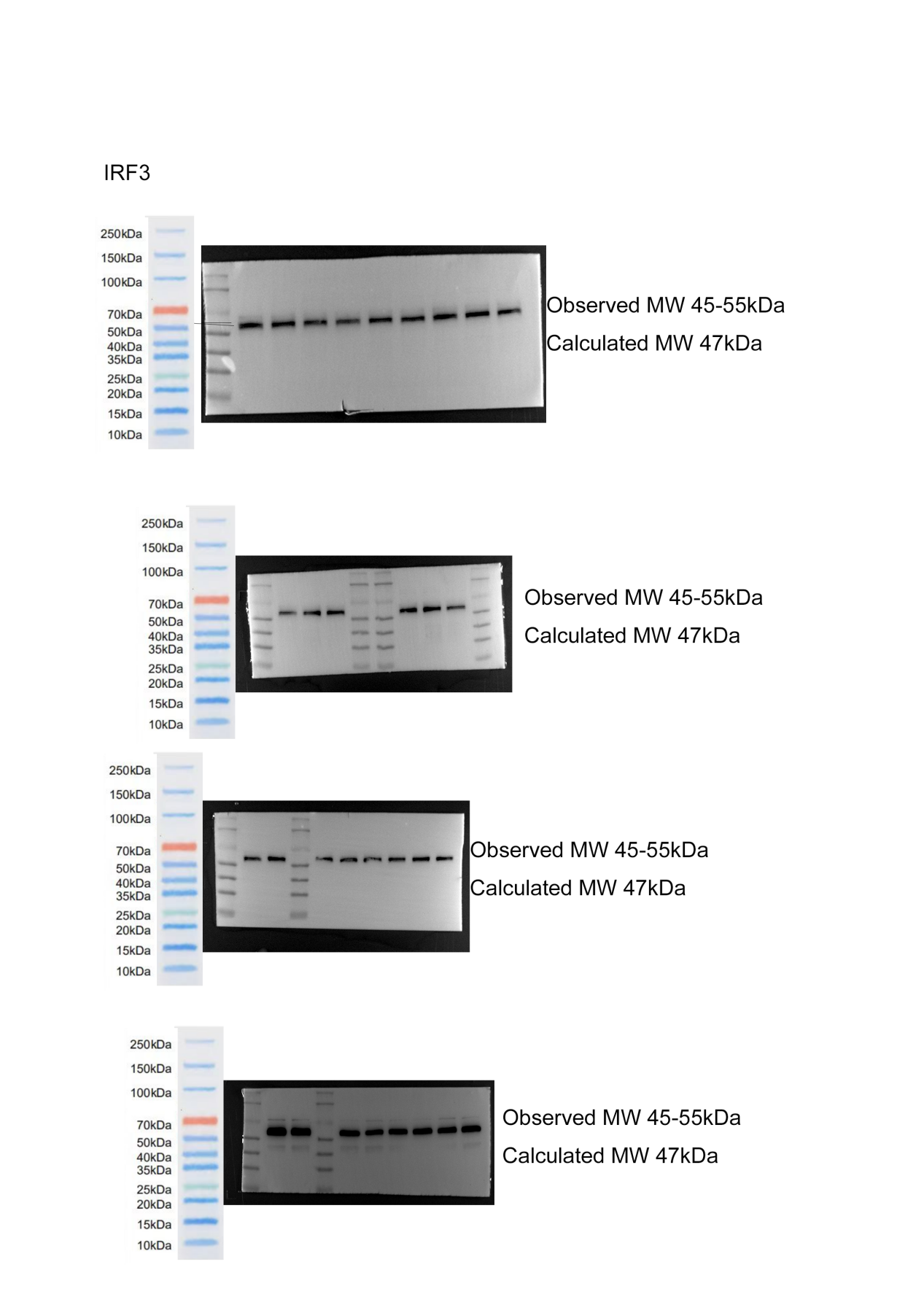


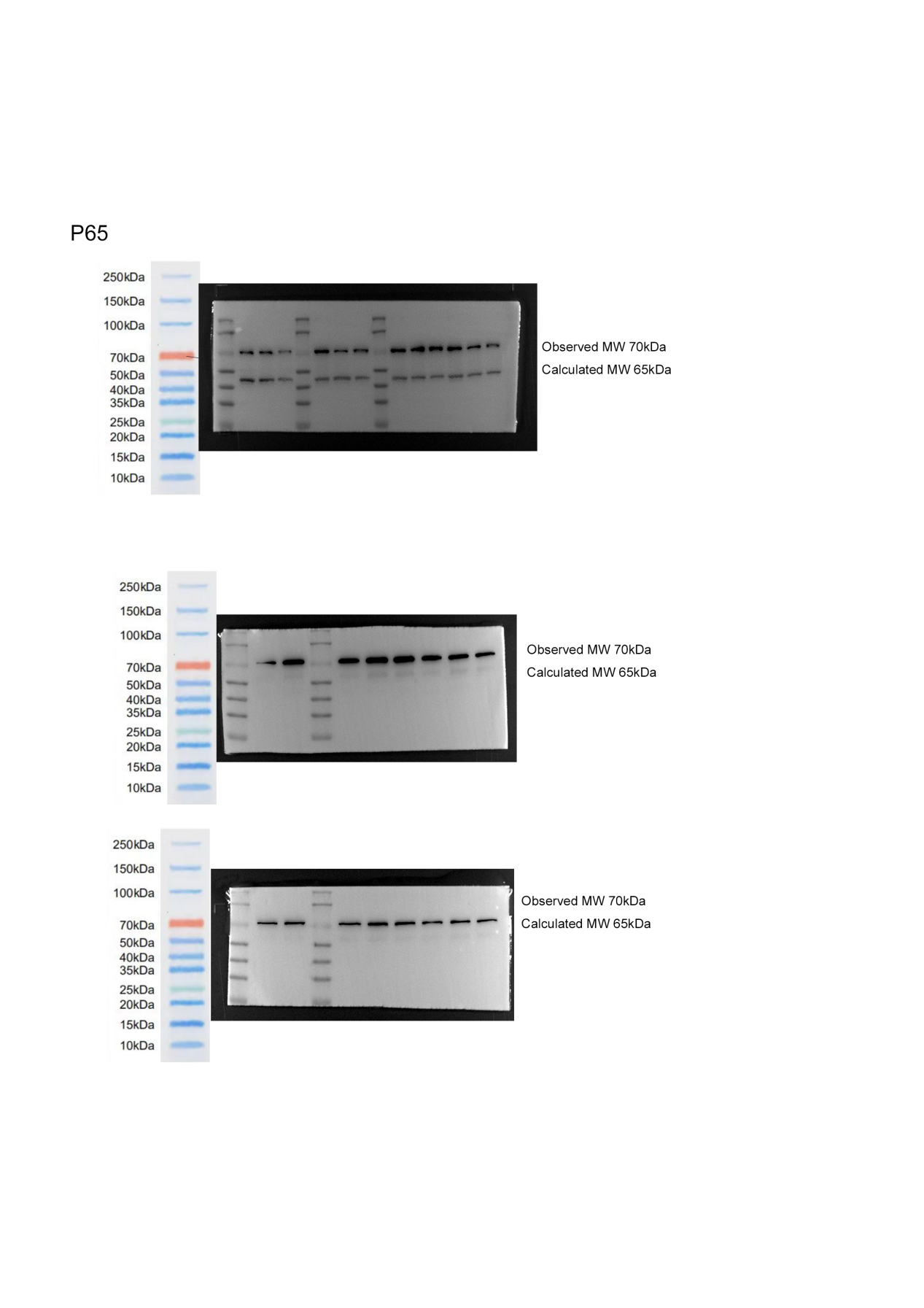


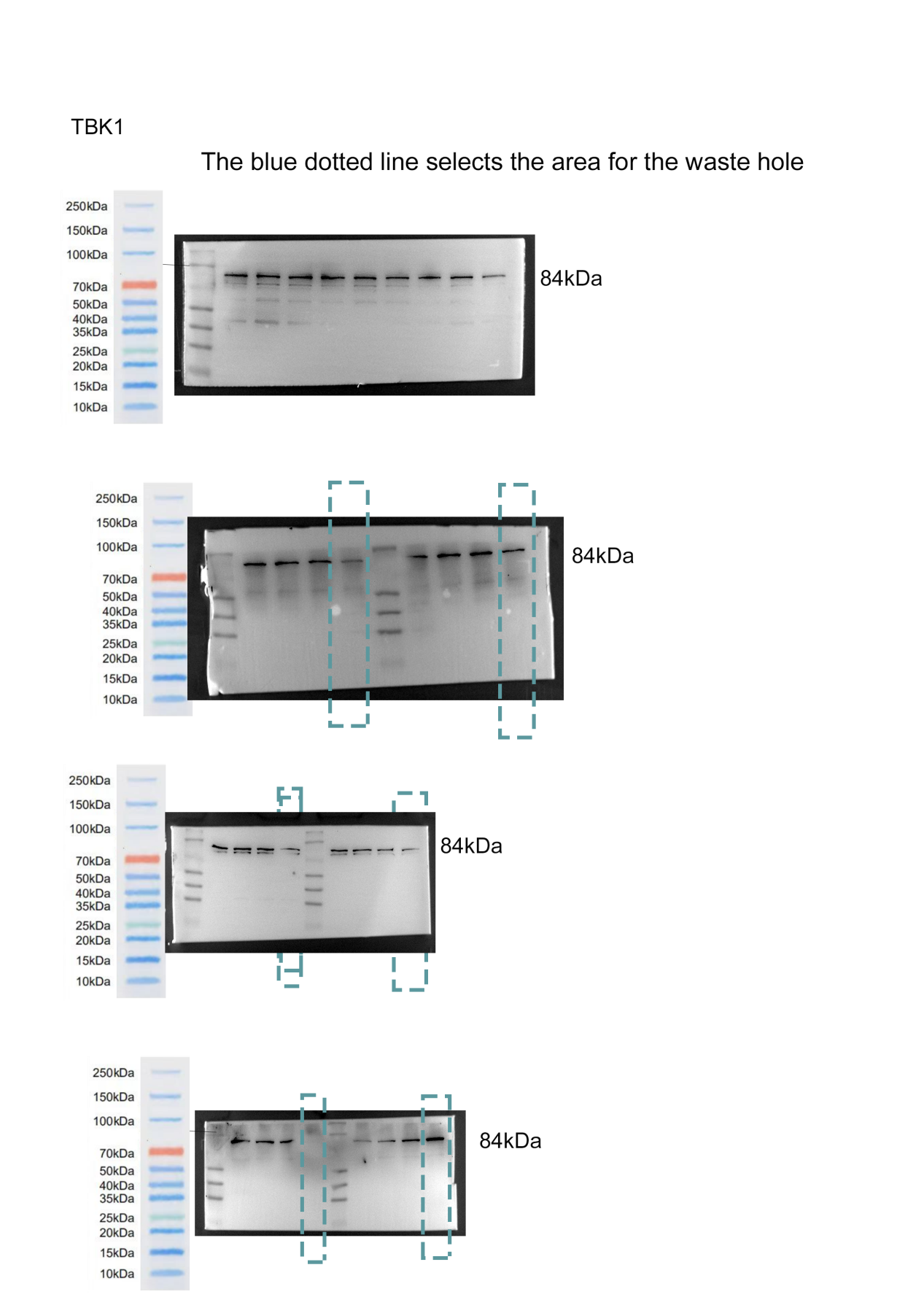

Supplement: Supplementary file 3 — Supplementary Material 3. [file 12964_2024_1677_MOESM3_ESM.docx]
