# Supplementary material for: Mitochondrial DNA release via the mitochondrial permeability transition pore activates the cGAS-STING pathway, exacerbating inflammation in acute Kawasaki disease
Source: Cell Commun Signal. 2024 Jun 13;22:328. doi: 10.1186/s12964-024-01677-9 (PMC11177463; doi:10.1186/s12964-024-01677-9)

| clinical data | HC  （n=60） | febrile  （n=60） | KD  （n=60） | P value |
| --- | --- | --- | --- | --- |
| Genders（M/F） | 40/20 | 32/28 | 32/28 | 0.45 |
| Age（y） | 4.53±2.62 | 3.61±2.07 | 4.59±11.42 | 0.69 |
| Height（cm） | 98.49±15.17 | 99.187±16.50 | 96.01±19.31 | 0.56 |
| Weight（Kg） | 15.62±4.08 | 15.20±4.87 | 15.69±6.91 | 0.87 |

Baseline characteristics of clinical data for each group

**Table 1** Primer Sequences of the Study

| **Genes（Human）** | **Forward primer 5ʹ→3ʹ** | **Reverse primer 5ʹ→3ʹ** |
| --- | --- | --- |
| GAPDH | CACGGCAAATTCCACGGCACAGT | GGGGGCATCAGCAGAAGGAGCAG |
| 18S | GTAACCCGTTGAACCCCATT | CCATCCAATCGGTAGTAGCG |
| ND1 | CCCTAAAACCCGCCACATCT | GAGCGATGGTGAGAGCTAAGGT |
| COX1 | TCATCTGTAGGCTCATTC | GGCATCCATATAGTCACT |
| IL-6 | GTCAACTCCATCTGCCCTTCAG | GGTCTGTTGTGGGTGGTATCCT |
| IP10 | AGAACTGTACGCTGTACCTG | GTAGCAATGATCTCAACACG |
| IFNα | GGAGGAGTTTGATGGCAACC | ATCCCAAGCAGCAGATGAAT |
| IFNβ | CTTGGATTCCTACAAAGAAGCAGC | TCCTCCTTCTGGAACTGCTGCA |

**Table 2** Primer Sequences of the Study

| **Genes（Mouse）** | **Forward primer 5ʹ→3ʹ** | **Reverse primer 5ʹ→3ʹ** |
| --- | --- | --- |
| GAPDH | GGTTGTCTCCTGCGACTTCA | TGGTCCAGGTTTCTTACTCC |
| 18S | TCCCCATGAACGAGGAATTC | CCGAGGGCCTCACTAAACC |
| ND1 | TTGCACCTACCCTATCACTCA | CGGCTCGTAAAGCTCCGAAT |
| COX1 | GACCGCAACCTAAACACAAC | GGTGCCCAAAGAATCAGAA |
| IL-6 | ACAGAAGGAGTGGCTAAGGACC | TAGGCATAACGCACTAGGTTT |
| IP10 | GTCATTTTCTGCCTCATCCT | GCCCTTTTAGACCTTTTTTG |
| IFNα  IFNβ | GCTAGGCTCTGTGCTTTCCT  AGCACTGGGTGGAATGAGAC | TCCTGCGGGAATCCAAAGTC  GAGTCCGCCTCTGATGCTTA |


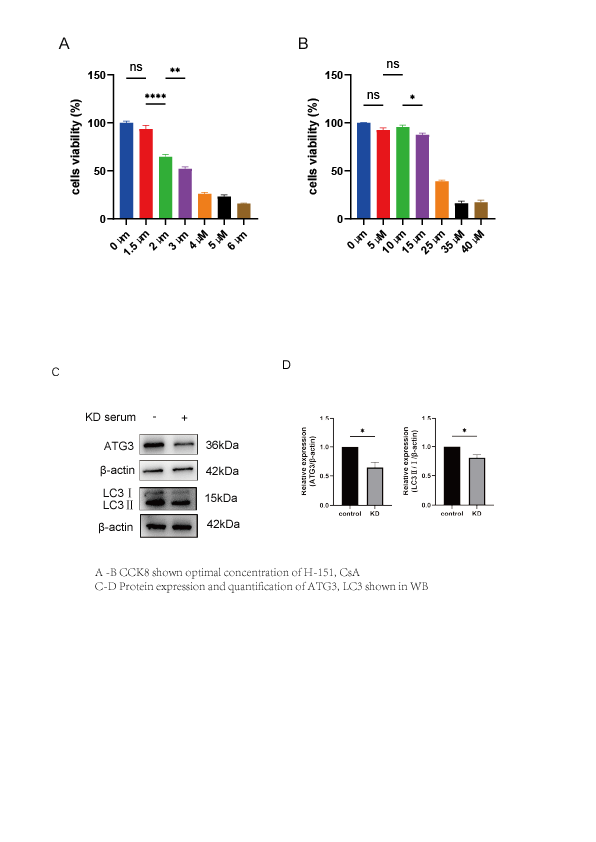


**The WB results analyses involved in this experiment were all *target gene/β-actin***

**Animal model ：**


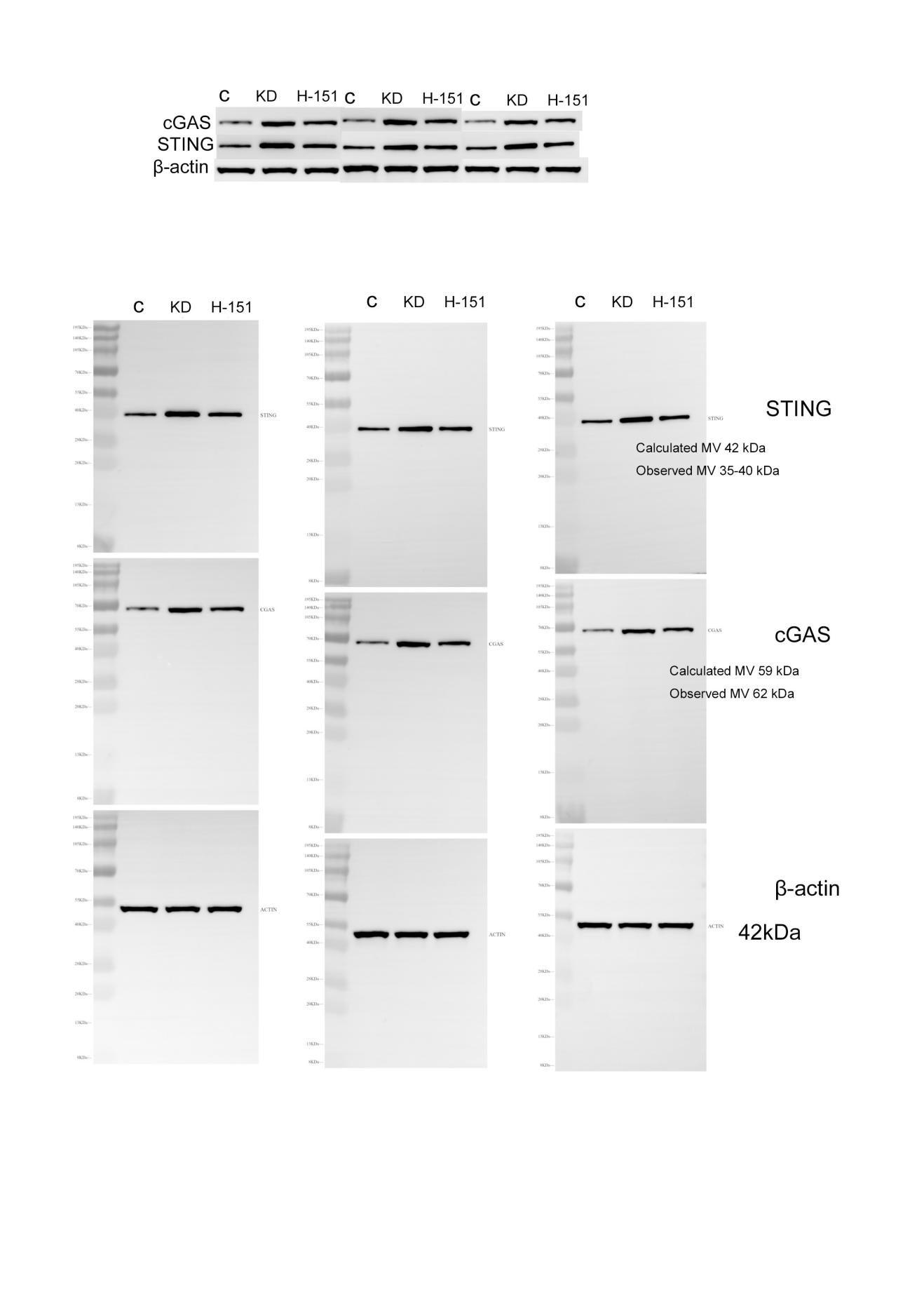


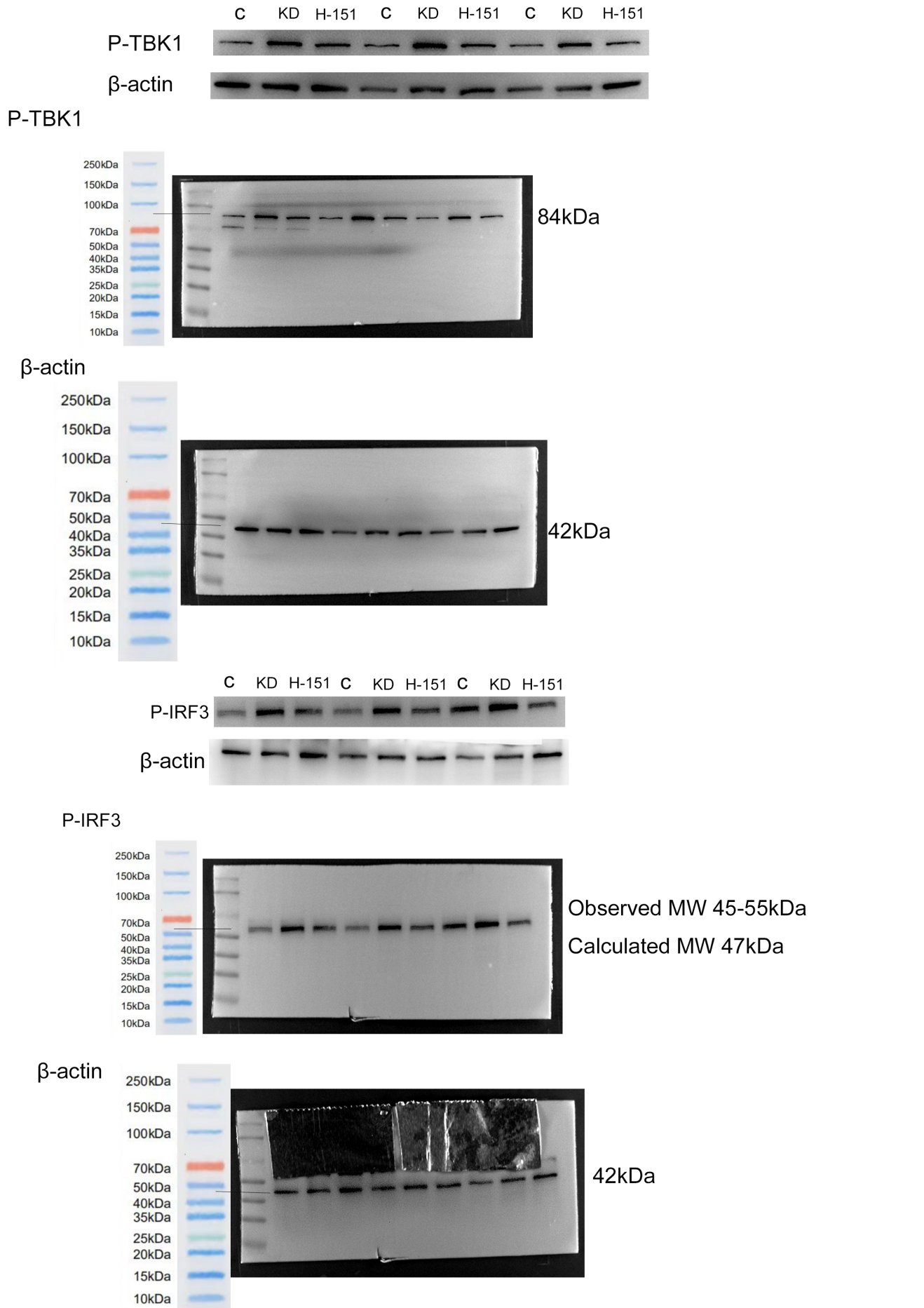


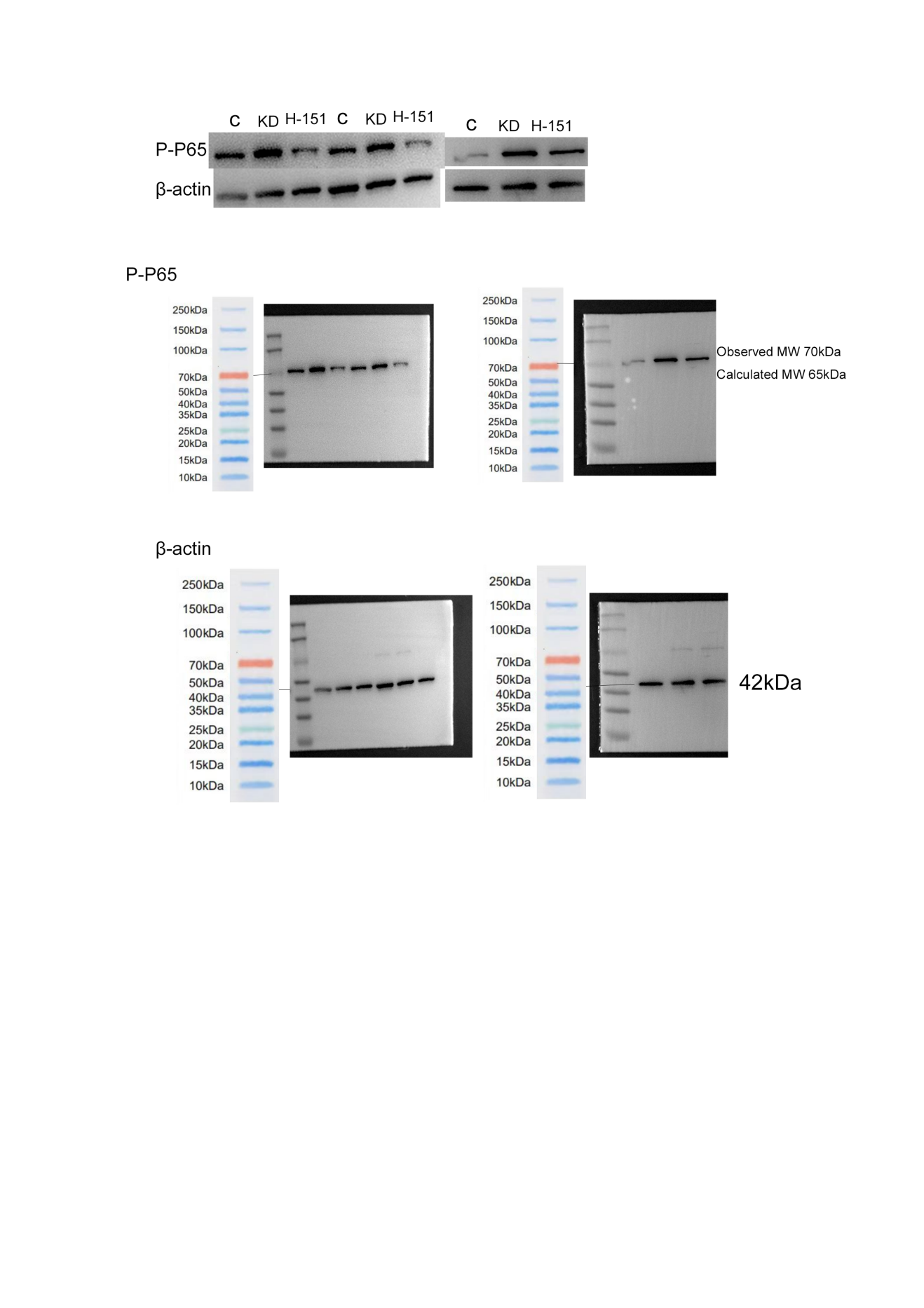


**Cell model:**


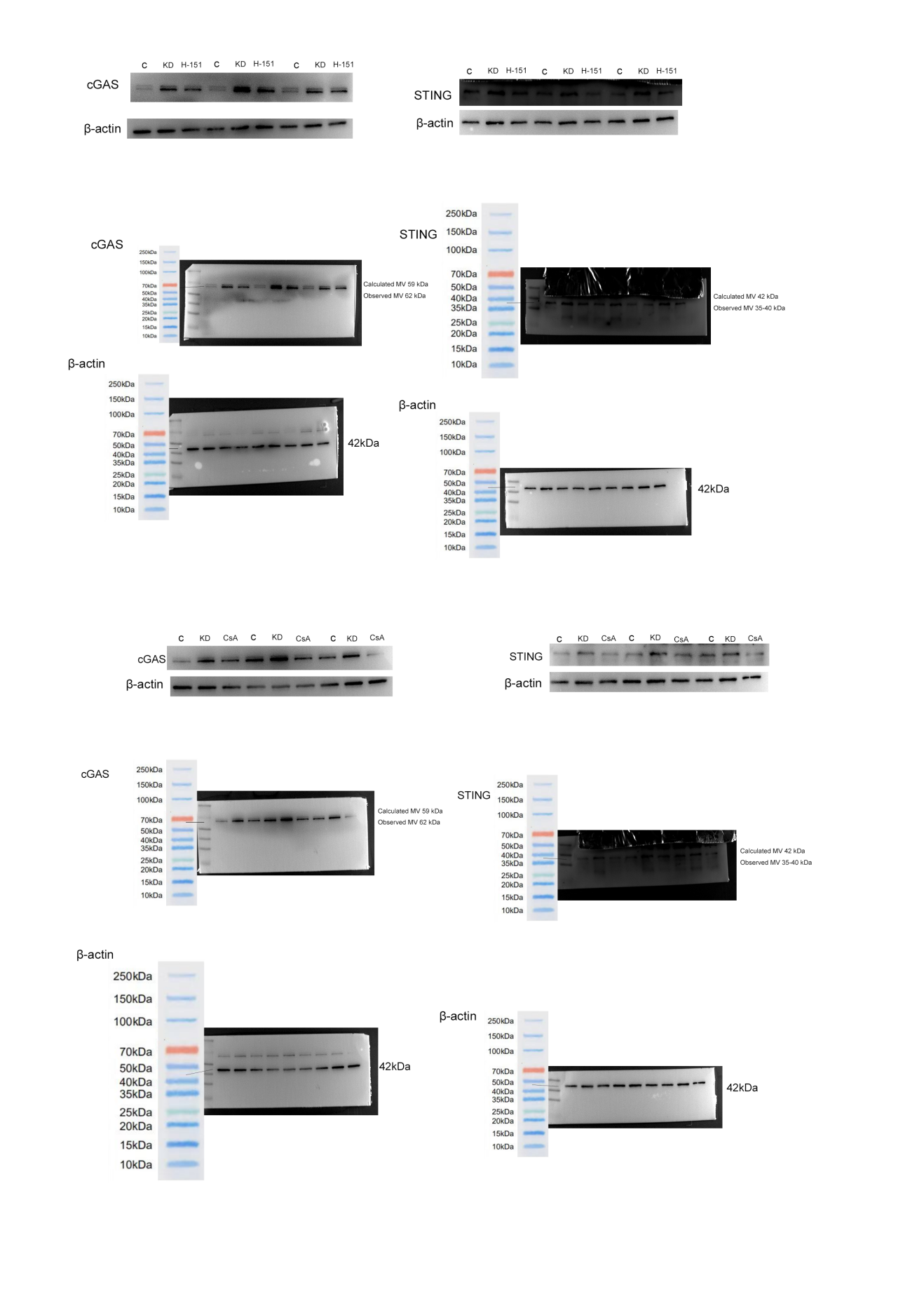


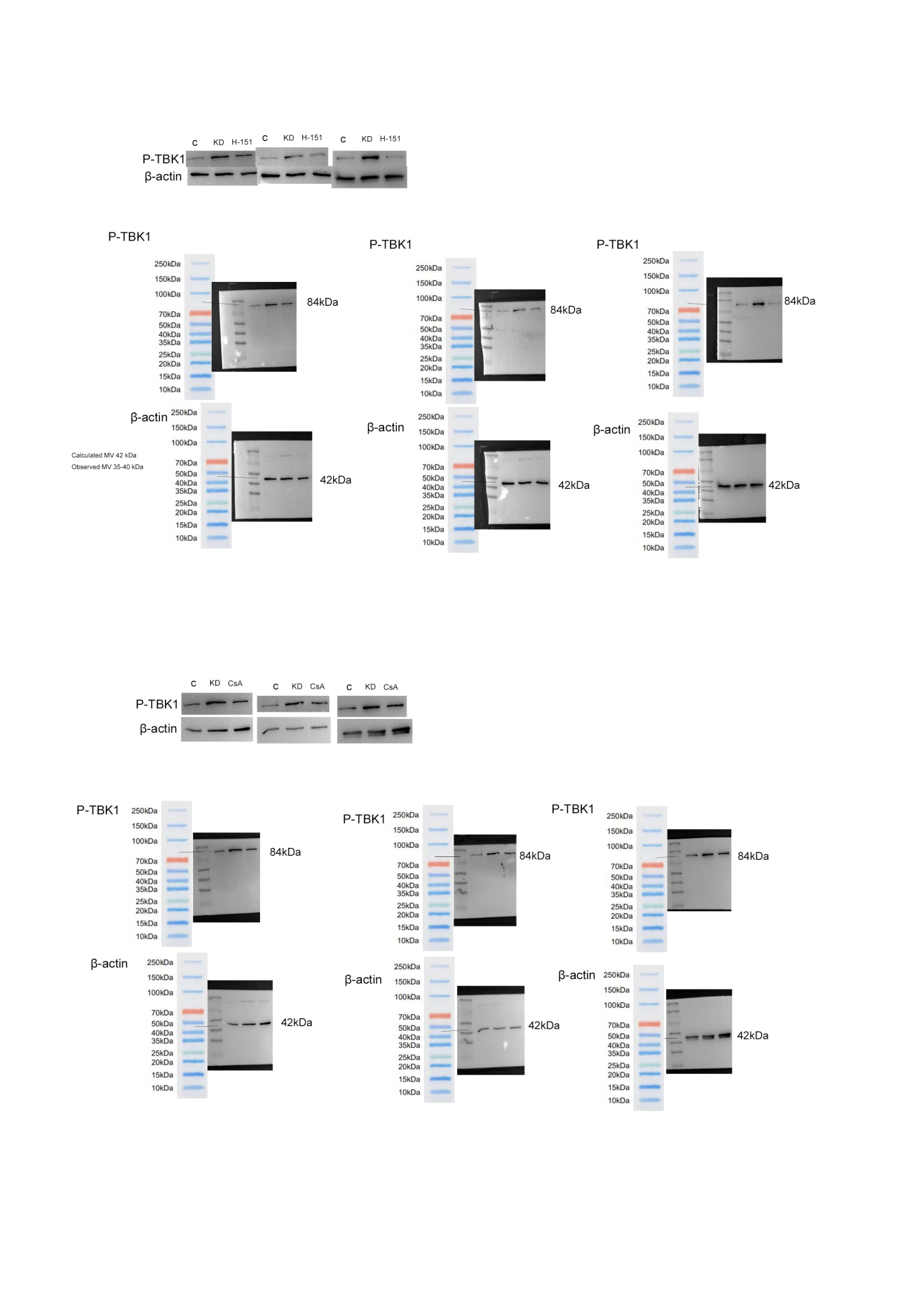


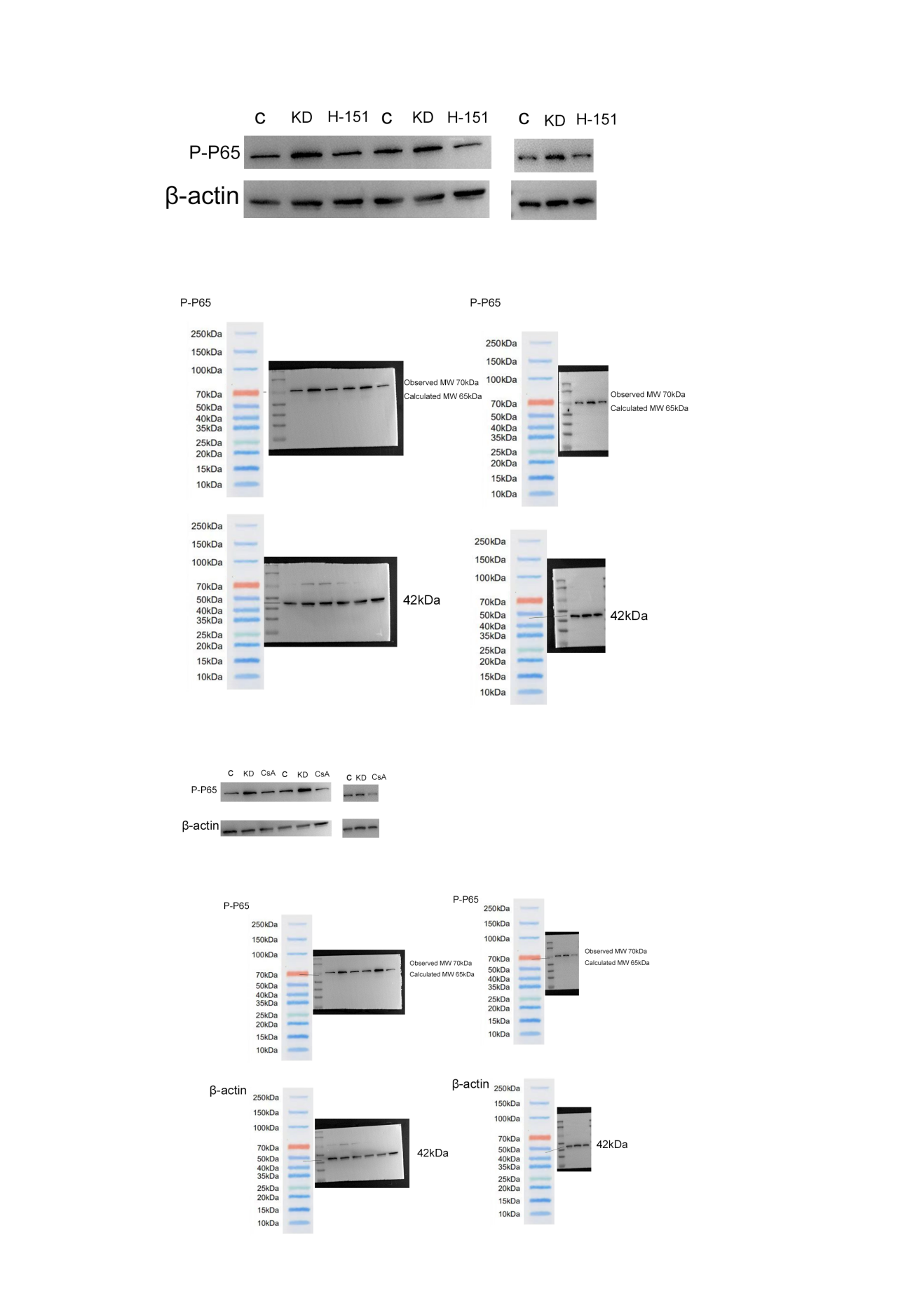


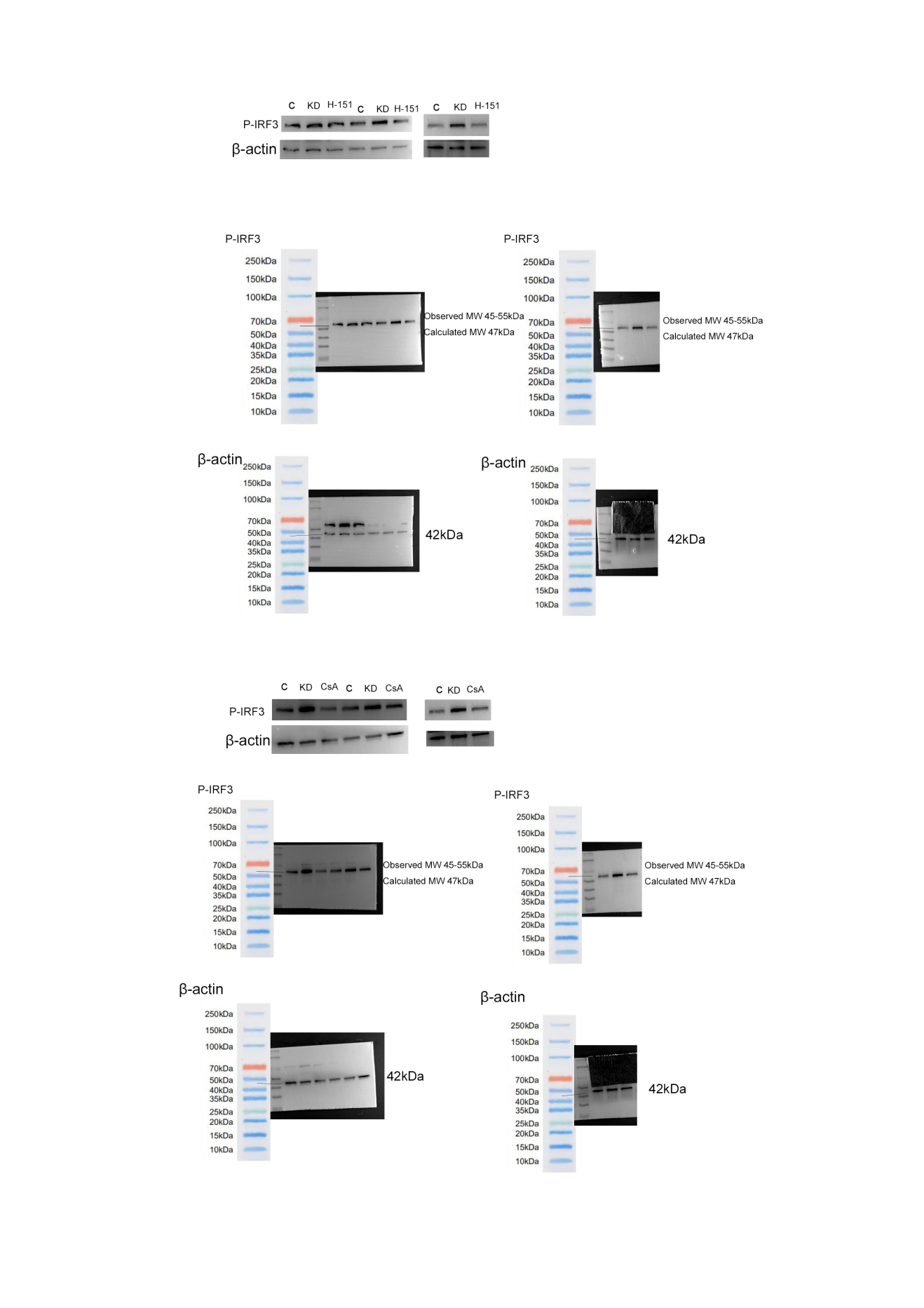


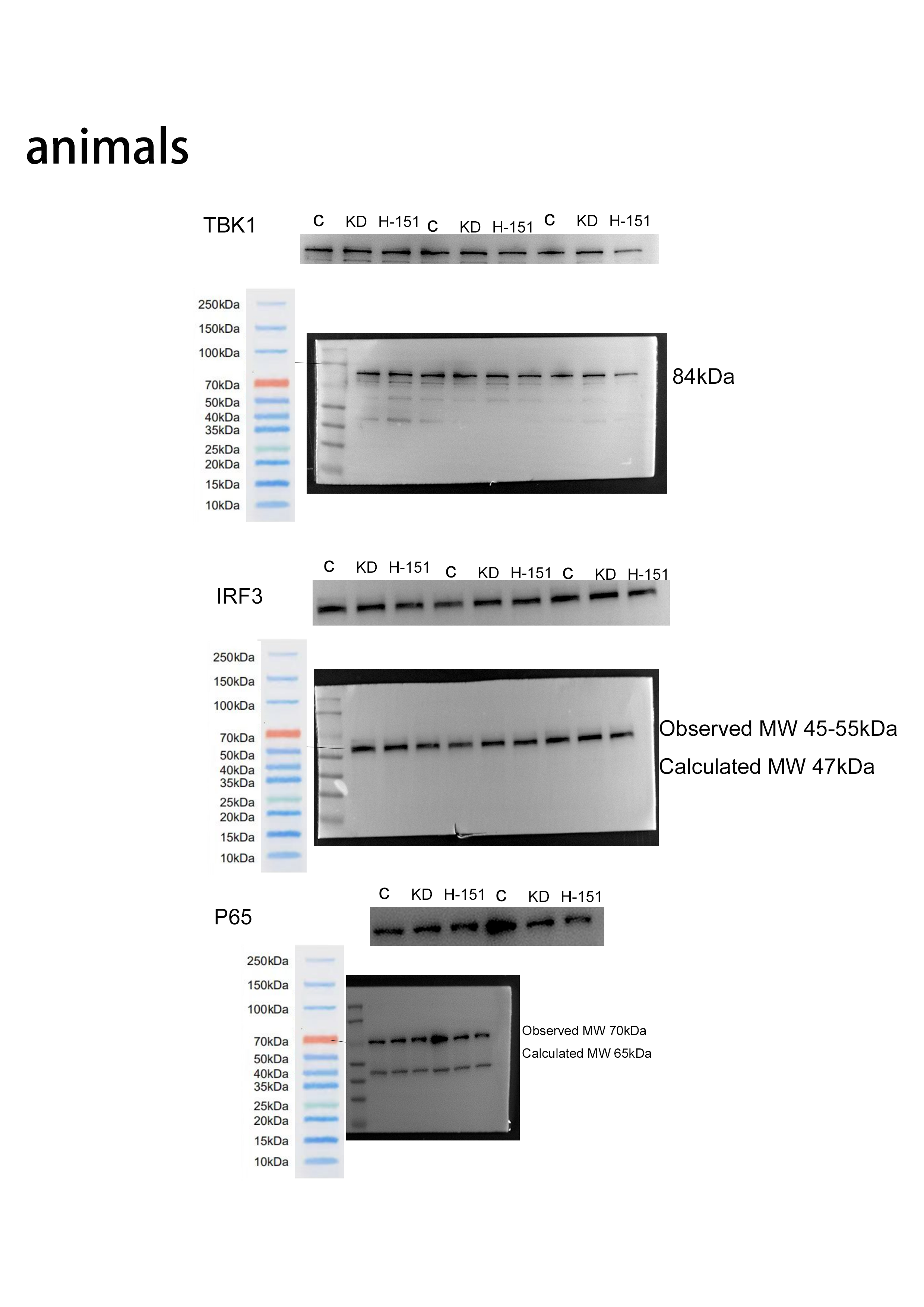


Total phosphorylated protein：


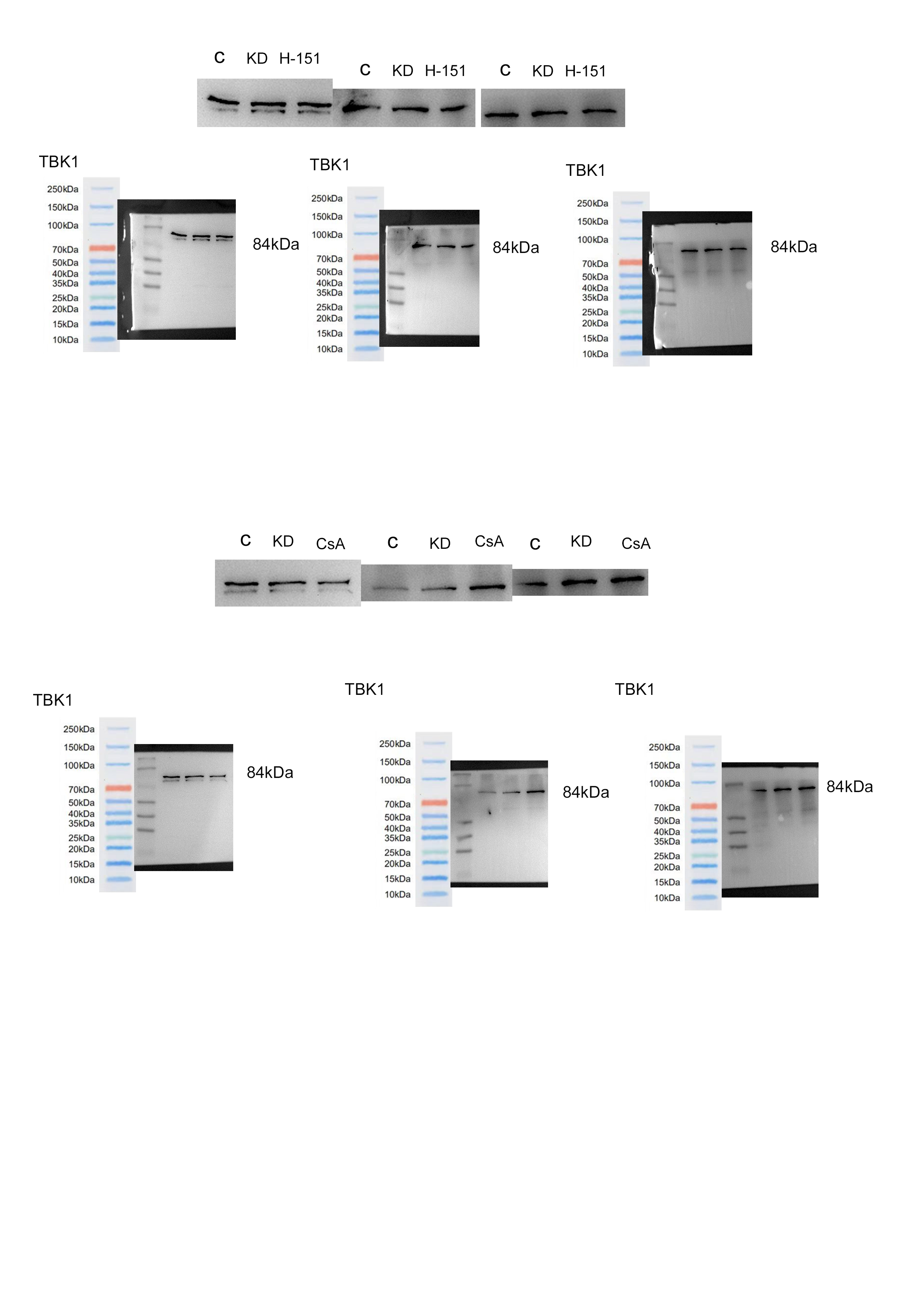


Total phosphorylated protein：

Cell:


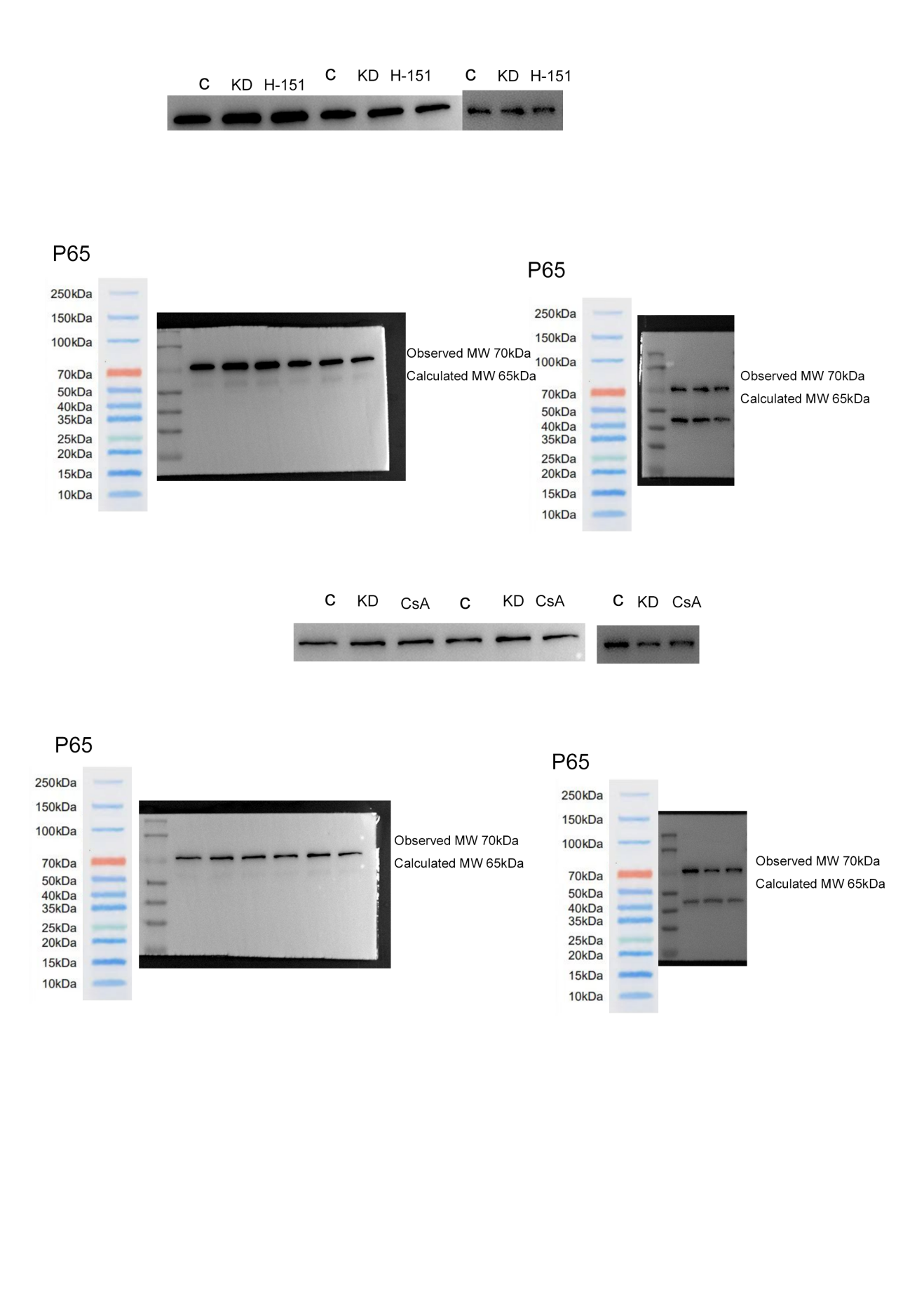


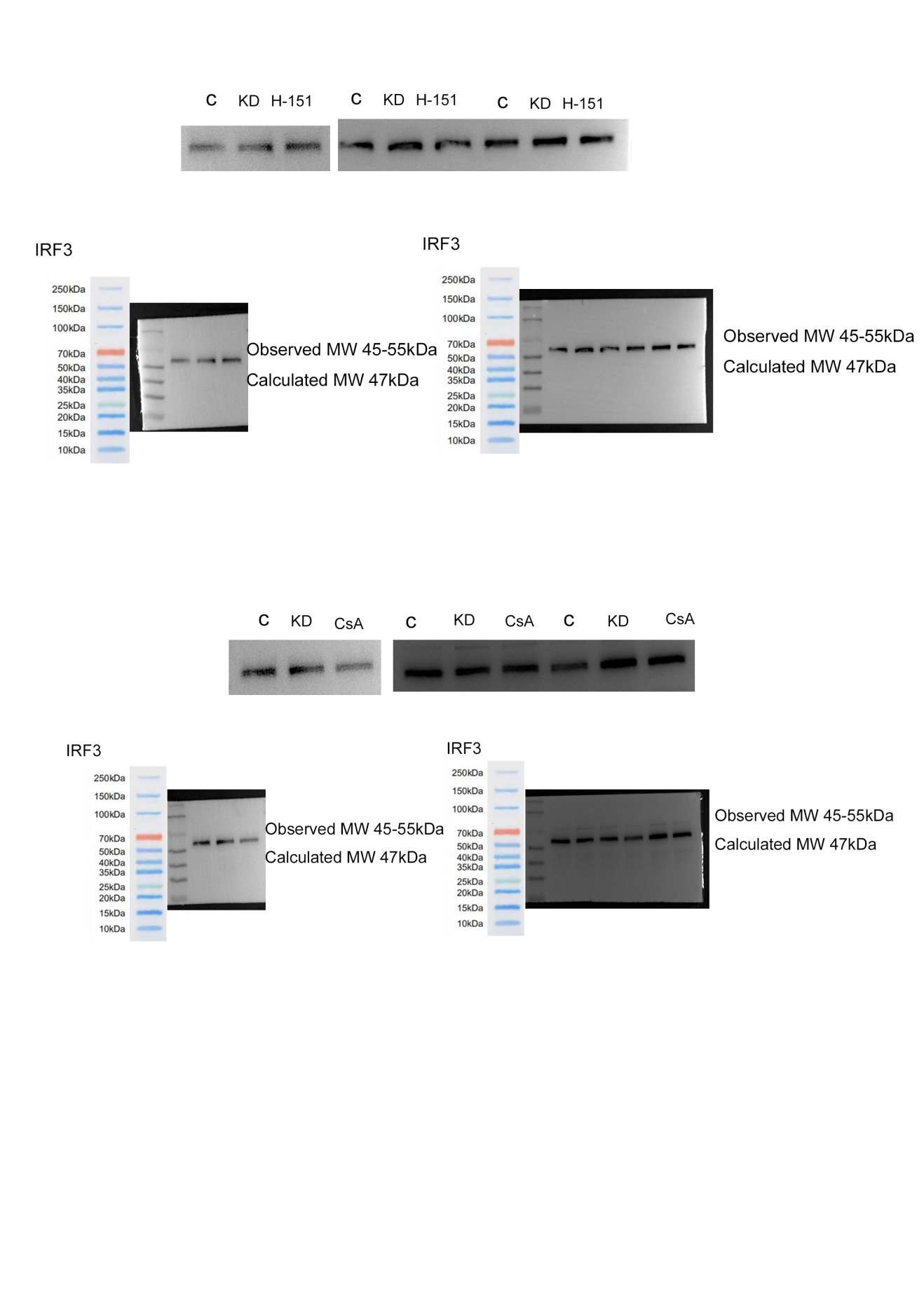


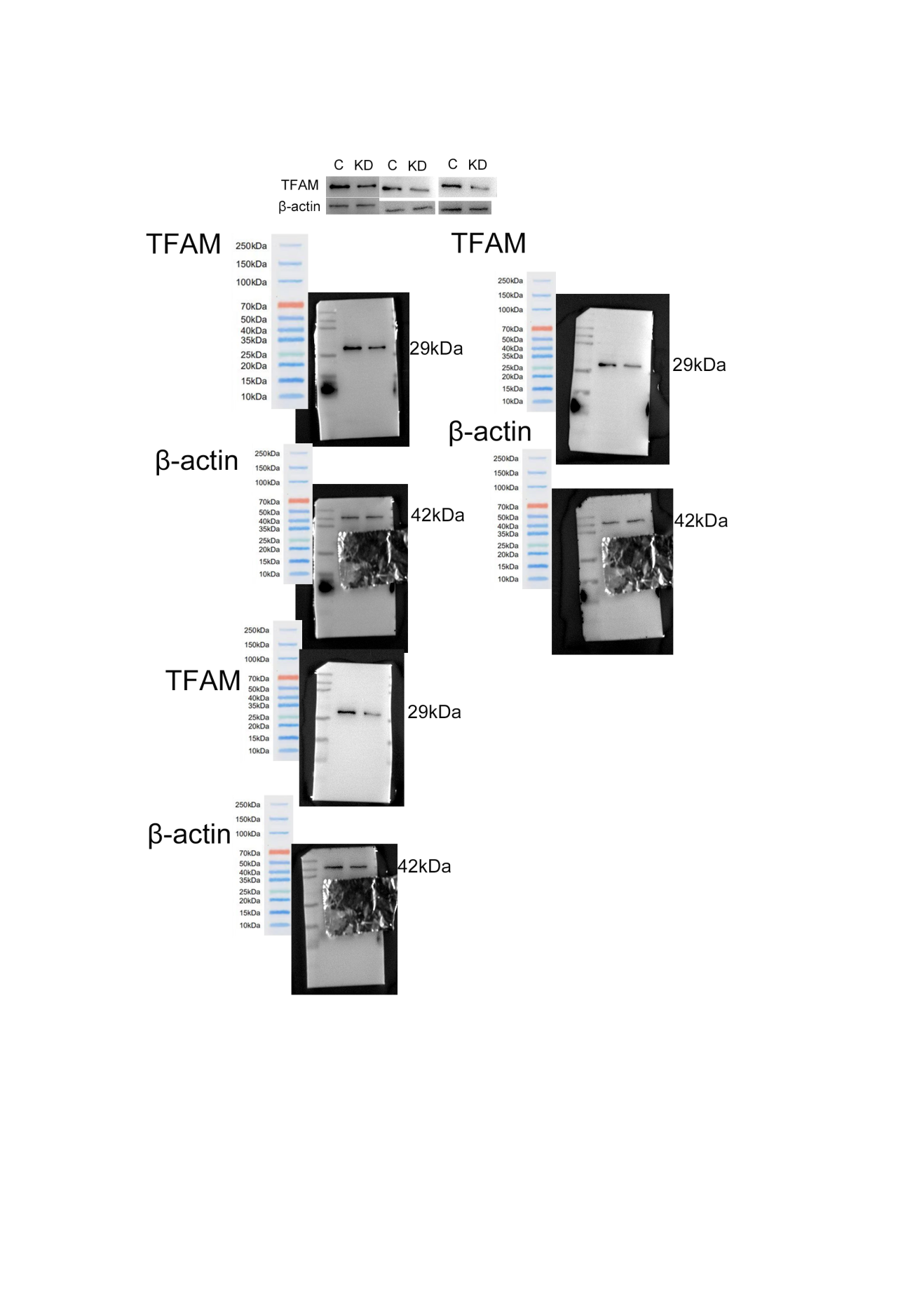


Cell:


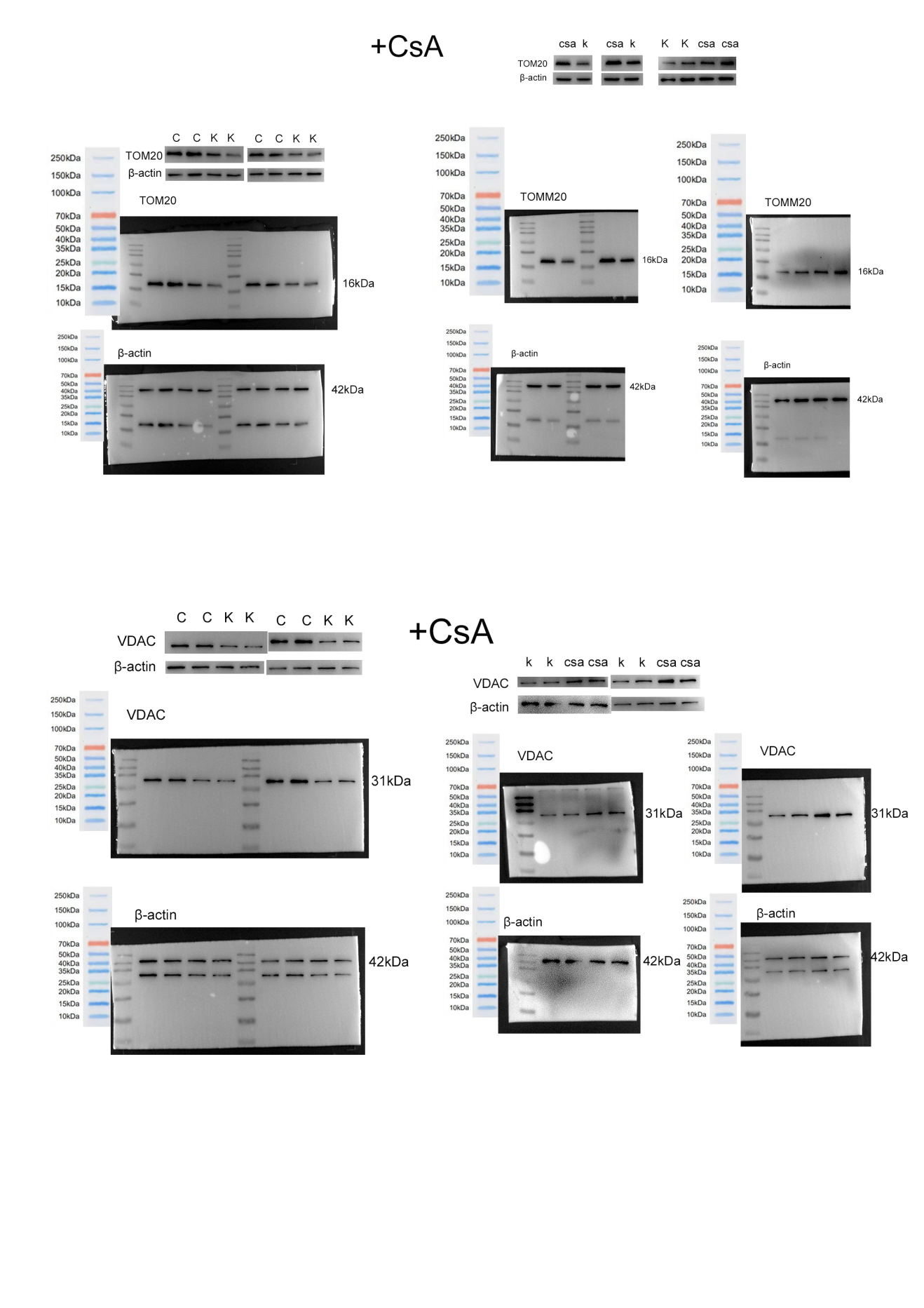


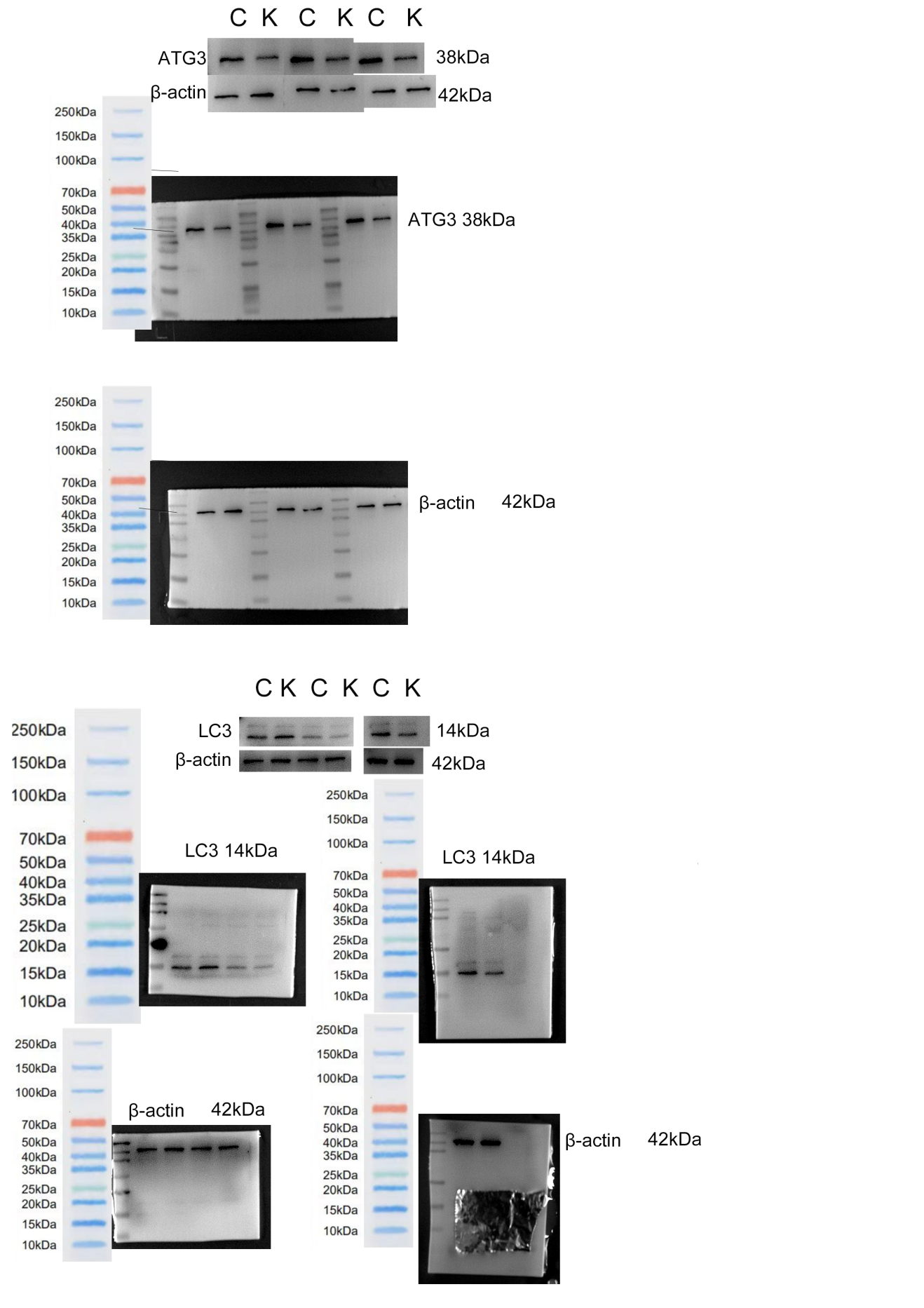

Supplement: Supplementary file 2 — Supplementary Material 2. [file 12964_2024_1677_MOESM2_ESM.docx]
